# Revaluation of biomass-derived furfuryl alcohol derivatives for the synthesis of carbocyclic nucleoside phosphonate analogues

**DOI:** 10.3762/bjoc.13.28

**Published:** 2017-02-09

**Authors:** Bemba Sidi Mohamed, Christian Périgaud, Christophe Mathé

**Affiliations:** 1 Institut des Biomolécules Max Mousseron (IBMM), UMR 5247, Université de Montpellier, CNRS, ENSCM, cc 1705, Site Triolet, Place Eugène Bataillon, 34095 Montpellier cedex 5, France

**Keywords:** analogue, antiviral, carbocyclic, nucleoside, phosphonate

## Abstract

The racemic synthesis of new carbocyclic nucleoside methylphosphonate analogues bearing purine bases (adenine and guanine) was accomplished using bio-sourced furfuryl alcohol derivatives. All compounds were prepared using a Mitsunobu coupling between the heterocyclic base and an appropriate carbocyclic precursor. After deprotection, the compounds were evaluated for their activity against a large number of viruses. However, none of them showed significant antiviral activity or cytotoxicity.

## Introduction

**Figure 1:**
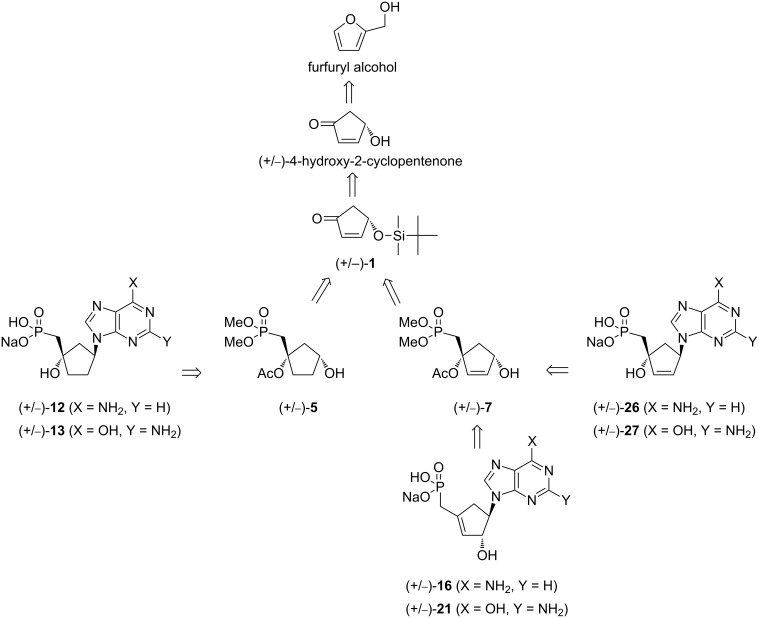
Retrosynthetic pathway for the synthesis of the target carbocyclic nucleoside methylphosphonates.

Biomass is a valuable resource in search of renewable organic carbon sources for the future and can be used to produce a range of chemical building blocks. The latter products can further be transformed to value-added compounds that are suitable for supplement or replacement to oil-derived chemicals. One such building block is furfuryl alcohol which is obtained through the catalytic hydrogenation of furfural; the latter is obtained from the dehydration of xylose, a 5-carbon sugar derived from vegetal biomass. Furfuryl alcohol finds widespread application in the chemical industries and for example is employed for the production of synthetic fibers, fine chemicals, etc. In fine organic chemicals synthesis, furfuryl alcohol is a raw material for the production of tetrahydrofurfuryl alcohol, which is an intermediate for the synthesis of 1,2- and 2,5-pentanediols and their derivatives and an agent for the manufacture of fragrance, vitamin C and lysine [1–2]. Furfuryl alcohol is also the source of 4-hydroxy-2-cyclopentenone, which, in enantiopure form, has been used as an intermediate for the synthesis of natural products and pharmaceutical drugs [3]. Recently, racemic (+/−)-4-hydroxy-2-cyclopentenone has found application in the synthesis of nucleoside analogues [4–5] and some of the products have shown interesting antiviral activities. As part of our studies on carbocyclic nucleoside phosphonates [6] as potential anti-HIV agents [7–8], we envisioned to use bio-sourced racemic (+/−)-4-*O*-protected 2-cyclopentenone for the synthesis of hitherto unknown carbocyclic nucleoside methylphosphonates (Figure 1) bearing purine bases (adenine and guanine) in order to evaluate their antiviral properties.

## Results and Discussion

### Synthesis of precursors (+/−)-**5** and (+/−)-**7**

**Scheme 1:**
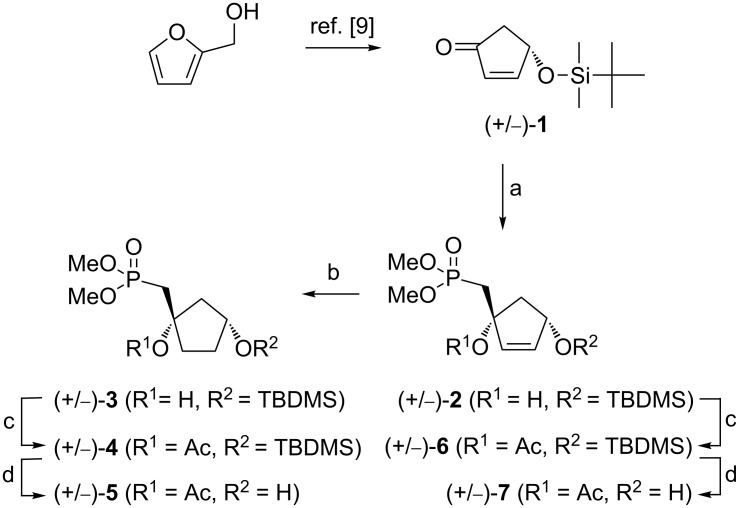
Reagents and conditions: (a) (CH_3_O)_2_P(O)CH_3_, *n*-BuLi, THF, −78 °C/rt, 2 h, 63%; (b) H_2_, Pd/C, MeOH, rt, 96%; (c) Ac_2_O, Et_3_N, DMAP, Et_2_O, rt, 88% (**4**), 88% (**6**); (d) TBAF (1 M), THF, 0 °C, 2 h, 85% (**5**), 76% (**7**).

The synthesis began with the preparation of racemic 4-*O*-TBDMS-2-cyclopentenone (**1**) which was obtained in two steps from commercially available furfuryl alcohol following a reported procedure (Scheme 1) [9]. Addition of the carbanion, generated in situ from dimethyl methylphosphonate and *n*-butyllithium in dry THF at −78 °C, to compound (+/−)-**1** gave cyclopentenyl alcohol (+/−)-**2** stereoselectively through a 1,2-addition mechanism. The stereochemistry of (+/−)-**2** may be ascribed to a nucleophilic attack of the incoming nucleophile from the less-hindered face due to the presence of the silyl protective group. ^1^H and ^13^C NMR spectra were in accordance with the presence of a sole diastereoisomer.

The reduction of compound (+/−)-**2** readily provided the saturated derivative (+/−)-**3** and the free tertiary hydroxy groups in (+/−)-**2** and (+/−)-**3** were then protected using acetic anhydride. Finally, removal of the TBDMS groups afforded the appropriate carbocyclic precursors, namely, (+/−)-1-((dimethoxyphosphoryl)methyl)-3-hydroxycyclopentyl acetate (**5**) and (+/−)-1-((dimethoxyphosphoryl)methyl)-4-hydroxycyclopent-2-en-1-yl acetate (**7**). The protection of the tertiary hydroxy groups was necessary in order to avoid competing side reactions during the coupling reaction under Mitsunobu conditions [10]. Compounds (+/−)-**5** and (+/−)-**7** were used as suitable precursors for the synthesis of the target carbocyclic methylphosphonates.

### Synthesis of cyclopentyl carbocyclic methylphosphonates (+/−)-**12** and (+/−)-**13**

**Scheme 2:**
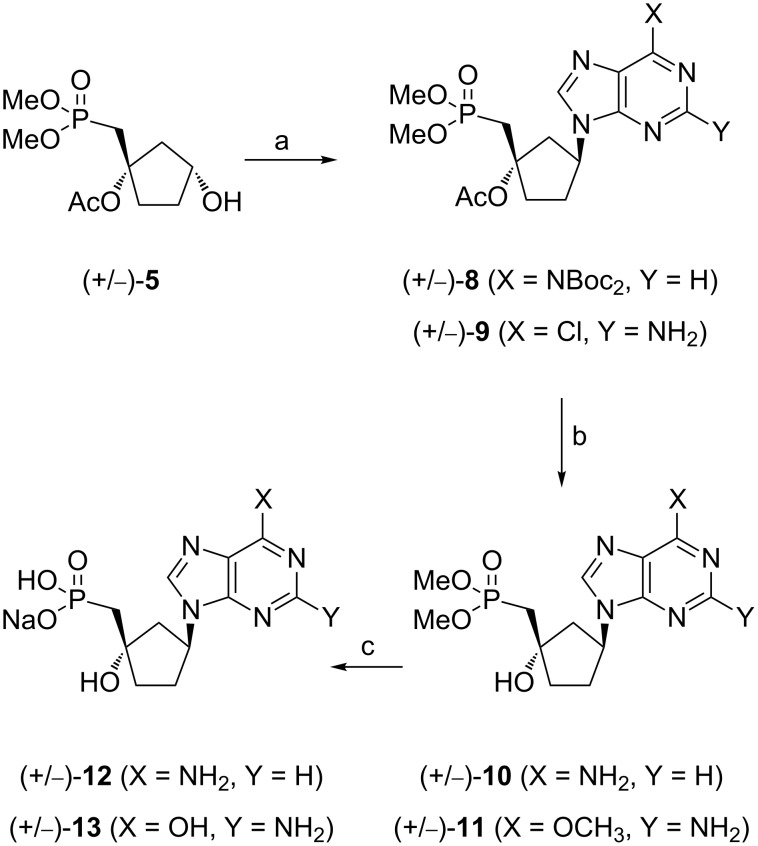
Reagents and conditions: (a) *N*^6^-bis-Boc-adenine or 2-amino-6-chloropurine, PPh_3_, DIAD, THF, 0 °C to rt, 42% (**8**), 54% (**9**); (b) for **10**: i) **8**, TFA, Cl(CH_2_)_2_Cl, rt; ii) K_2_CO_3_, MeOH, rt, 60%; for **11**: **9**, K_2_CO_3_, MeOH, rt, 68%; (c) for **12**: **10**, TMSCl, NaI, CH_3_CN, DMF, 40 °C to rt, 61%; for **13**: **11**, TMSBr, DMF, 0 °C to rt, 35%.

The synthesis of the target compounds was accomplished using a Mitsunobu reaction (Scheme 2) [11]. The coupling of (+/−)-**5** with bis-Boc-adenine or 2-amino-6-chloropurine in the presence of diisopropyl azodicarboxylate (DIAD) and PPh_3_, provided the N9 carbocyclic nucleosides (+/−)-**8** and (+/−)-**9** as racemic mixtures. No concomitant formation of the N7 regioisomer was observed.

The removal of Boc and acetyl groups from (+/−)-**8** afforded the carbocyclic phosphonate (+/−)-**10**. Subsequently, the phosphonoester protecting groups were cleaved in the presence of TMSCl and NaI in a CH_3_CN/DMF mixture to give (+/−)-**12** in 61% yield. The treatment of (+/−)-**9** with K_2_CO_3_ in methanol at room temperature gave compound (+/−)-**11** which upon reaction with TMSBr in DMF led to a deprotection of the diester groups as well as a concomitant hydrolysis of the methoxy group. Both compounds, (+/−)-**12** and (+/−)-**13**, were obtained as their sodium salts, after reversed phase column chromatography and ion exchange chromatography.

### Synthesis of cyclopentenyl carbocyclic methylphosphonates

#### Compounds (+/−)-**16** and (+/−)-**21**

**Scheme 3:**
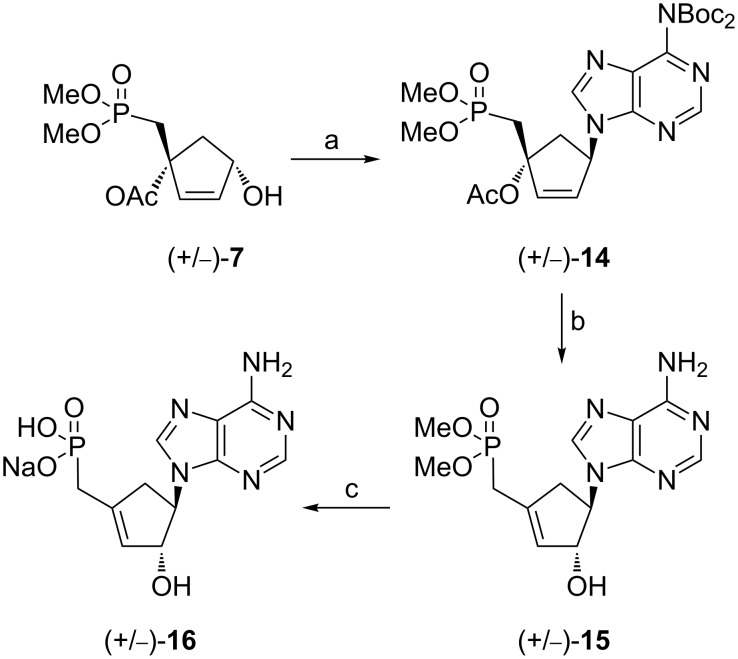
Reagents and conditions: (a) *N*^6^-bis-Boc-adenine, PPh_3_, DIAD, THF, rt, 56%; (b) TFA, Cl(CH_2_)_2_Cl, rt, 85%; (c) TMSCl, NaI, CH_3_CN, 40 °C to rt, 60%.

The synthesis of the cyclopentenyl carbocyclic derivatives was envisioned from the precursor (+/−)-**7** (Scheme 3) using Mitsunobu conditions. A coupling reaction of (+/−)-**7** with bis-Boc-adenine [12] afforded the desired adduct (+/−)-**14** with 56% yield. The removal of the Boc groups was achieved following a similar protocol as developed for compound **8**.

**Figure 2:**
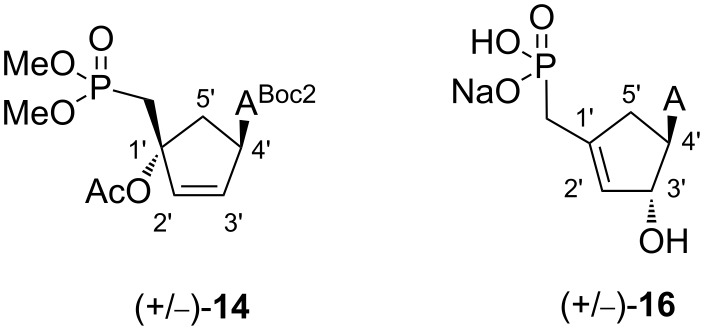
Numbering for **14** and **16**.

Surprisingly, the treatment of compound (+/−)-**14** under acidic conditions lead to the removal of the Boc group accompanied with an unexpected transposition of the allyl moiety and a concomitant loss of AcOH leading to compound (+/−)-**15** with 85% yield. The 1,3-allylic transposition of the hindered tertiary alcohol group under acidic conditions has not been reported yet for such compounds. It may conceivably that such a transposition occurs through the formation of an allylic carbocation which upon reaction with water as an incoming nucleophile, afforded compound (+/−)-**15** with a higher substituted double bond. After deprotection of the phosphonate diester groups, structural assignments of (+/−)-**16** were based upon ^1^H and ^13^C NMR spectra and correlation experiments, which showed some characteristic features compared to the parent derivative (+/−)-**14**. In particular, the chemical shifts of C2’, C3’ and C1’ carbon atoms showed differences (Table 1). In case of compound (+/−)-**14**, the chemical shifts of C2’ and C3’ are consistent with sp^2^-hybridized carbons while for compound (+/−)-**16**, chemical shifts corresponding to sp^2^ carbons were detected for C2’ and C1’ (Figure 2)_._ Furthermore an upfield shift of the signals for C2’ was also observed for compounds (+/−)-**14** and (+/−)-**16**, respectively. Inversely, a downfield shift for C1’ was observed for compound (+/−)-**14** compared to (+/−)-**16**. These observations are in agreement with a 1,3-allylic transposition under acidic conditions.

**Table 1:** Selected chemical shifts in ^13^C NMR.^a^

	(+/−)-**14**	(+/−)-**16**

C3’	134.5	81.5
C2’	138.2	125.6
C1’	88.7	143.0

^a^δ in ppm of C2’, C3’ and C1’_._

**Figure 3:**
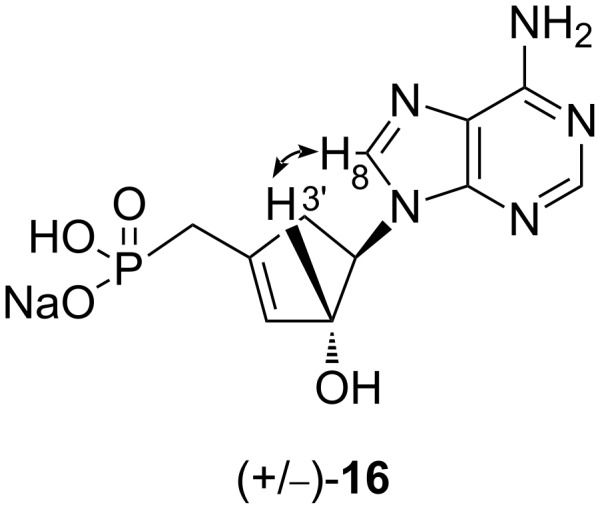
Selected NOESY correlations for compound (+/−)-**16**.

Additionally, in order to confirm the stereochemistry of the allylic alcohol in position 3’, a NOESY correlation experiment was accomplished with compound (+/−)-**16** (Figure 3). We have observed a correlation between proton H8 and H3’ confirming the orientation of the 3’-OH.

**Scheme 4:**
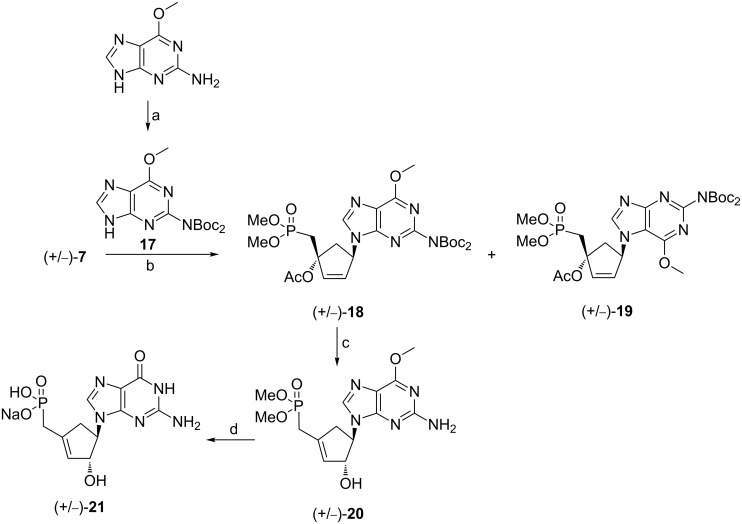
Reagents and conditions: (a) i) Boc_2_O, DMAP, THF, rt; ii) K_2_CO_3_, MeOH, 75%; (b) PPh_3_, DIAD, THF, rt, 55% for **18**; 5% for **19**; (c) TFA, Cl(CH_2_)_2_Cl, rt, 51%; (d) TMSBr, DMF, 0 °C to rt, 59%.

From the results obtained with adenine, we envisioned to synthesize the parent nucleoside of (+/−)-**16** bearing guanine as the base. We chose as a precursor of the heterocyclic base the commercially available 2-amino-6-methoxypurine which upon treatment with Boc_2_O afforded the suitable heterocyclic precursor **17** (Scheme 4). The coupling reaction of (+/−)-**7** and **17** using the Mitsunobu reaction gave a separable mixture of N9/N7 regioisomers (+/−)-**18** and (+/−)-**19** with 55% and 5% yield, respectively. After purification, compound (+/−)-**18** was treated under acidic conditions to remove the Boc group as well as to induce a transposition of the allyl moiety in a similar manner to the one previously observed with compound (+/−)-**14**. Finally, treatment of (+/−)-**20** with TMSBr in DMF led to the cleavage of the phosphonoester groups and concomitant hydrolysis of the methoxy group. Compound (+/−)-**21** was obtained as sodium salt after reversed phase column chromatography and ion exchange chromatography and its structural assignments were based upon ^1^H and ^13^C NMR spectra and correlation experiments. It is noteworthy that the carbocyclic methylphosphonates (+/−)-**16** and (+/−)-**21** have structural similarity with carbonucleosides belonging to the neplanocin family, in particular, with neplanocin F [13].

#### Compounds (+/−)-**26** and (+/−)-**27**

**Scheme 5:**
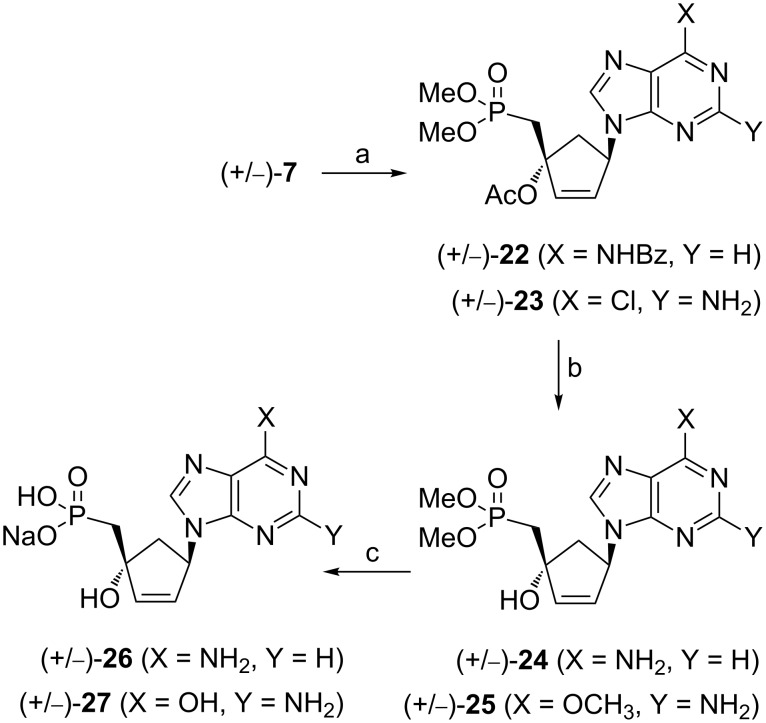
Reagents and conditions: (a) *N*^6^-Bz-adenine or 2-amino-6-chloropurine, PPh_3_, DIAD, THF, 0 °C to rt, 17% for **22**, 53% for **23**; (b) K_2_CO_3_, MeOH, rt, 34% for **24**, 72% for **25**; (c) TMSBr, DMF, 0 °C to rt, 12% for **26**, 17% for **27**.

Based on the observation that carbocyclic methylphosphonates derived from the Mitsunobu coupling reaction are sensitive to treatment in acidic medium, the synthesis of the target compounds (+/−)-**26** and (+/−)-**27** was envisaged through the use of precursors without acid-labile protecting groups. Thus, reaction of (+/−)-**7** with *N*^6^-Bz-adenine [14] or 2-amino-6-chloropurine in the presence of PPh_3_ and DIAD in THF provided the Mitsunobu adducts (+/−)-**22** and (+/−)-**23** with 17% and 53% yield, respectively (Scheme 5). A lower yield was observed for the coupling reaction of *N*^6^-Bz-adenine with (+/−)-**7** compared the reaction with of *N*^6^-bis-Boc-adenine (17% versus 56%, Scheme 3). In both cases, no formation of the N7 alkylation product was observed.

The treatment of (+/−)-**22** and (+/−)-**23** with K_2_CO_3_ in methanol at room temperature afforded compounds (+/−)-**24** and (+/−)-**25**. Cleavage of the ester groups and in case of (+/−)-**25,** hydrolysis of the methoxy group, was achieved by reaction with TMSBr in DMF. Compounds (+/−)-**26** and (+/−)-**27** were obtained as their sodium salts after reversed phase column chromatography and ion exchange chromatography.

## Conclusion

In summary, we have developed a methodology for the synthesis of carbocyclic nucleoside phosphonate analogues through the use of bio-sourced furfuryl alcohol derivatives. The methodology involved the preparation of the proper carbocyclic phosphonate precursors which upon Mitsunobu reaction with the appropriate heterocyclic bases afforded the protected target intermediates. Some unsaturated derivatives have shown instability in acidic medium and underwent an unexpected 1,3-allylic transposition giving rise to carbocyclic nucleoside phosphonates having structural similarity with carbonucleosides belonging to the neplanocin family. All the newly synthesized compounds were evaluated for their antiviral properties against HIV-1, Zika virus, Dengue-2 virus, HSV-1, HSV-2 and Chikungunya virus. However, none of them showed significant antiviral or cytotoxic activities. The absence of biological activity may be attributed to various factors, such as inability to enter cells or to behave as substrates for intracellular enzymes catalyzing phosphorylation, as well as a lack of inhibition of viral polymerases by their diphospho–phosphonate forms.

## Supporting Information

File 1 Synthetic details and characterization data of new compounds.

File 2 Copies of NMR spectra for the synthesized compounds.

## References

[1] Corma A, Iborra S, Velty A (2007). Chem Rev.

[2] Halilu A, Ali T H, Atta A Y, Sudarsanam P, Bhargava S K, Hamid S B A (2016). Energy Fuels.

[3] Roche S P, Aitken D J (2010). Eur J Org Chem.

[4] Mantione D, Aizpuru O O, Memeo M G, Bovio B, Quadrelli P (2016). Eur J Org Chem.

[5] Ulbrich K, Kreitmeier P, Vilaivan T, Reiser O (2013). J Org Chem.

[6] Uttaro J-P, Broussous S, Mathé C, Périgaud C (2013). Tetrahedron.

[7] Boojamra C G, Parrish J P, Sperandio D, Gao Y, Petrakovsky O V, Lee S K, Markevitch D Y, Vela J E, Laflamme G, Chen J M (2009). Bioorg Med Chem.

[8] Macchi B, Romeo G, Chiacchio U, Frezza G, Giofrè S V, Marino-Merlo F, Mastino A (2015). Top Med Chem.

[9] Curran T T, Hay D A, Koegel C P, Evans J C (1997). Tetrahedron.

[10] Mitsunobu O (1981). Synthesis.

[11] Hughes D L (1996). Org Prep Proced Int.

[12] Michel B Y, Strazewski P (2007). Tetrahedron.

[13] Rodriguez S, Edmont D, Mathé C, Périgaud C (2007). Tetrahedron.

[14] Milecki J, Foldesi A, Fischer A, Adamiak W R, Chatopadhyaya J (2001). J Labelled Compd Radiopharm.

