# Supplementary material for: Revaluation of biomass-derived furfuryl alcohol derivatives for the synthesis of carbocyclic nucleoside phosphonate analogues
Source: Beilstein J Org Chem. 2017 Feb 9;13:251–6. doi: 10.3762/bjoc.13.28 (PMC5331271; doi:10.3762/bjoc.13.28)
Supplement: File 1 — Synthetic details and characterization data of new compounds. [file Beilstein_J_Org_Chem-13-251-s001.pdf]

Supporting Information File 1

for

Revaluation of biomass-derived furfuryl alcohol derivatives for the synthesis of carbocyclic nucleoside phosphonate analogues

Bemba Sidi Mohamed, Christian Périgaud and Christophe Mathé*

Address: Institut des Biomolécules Max Mousseron (IBMM), UMR 5247, Université de Montpellier, CNRS, ENSCM, cc 1705, Site Triolet, Place Eugène Bataillon

34095 Montpellier cedex 5, France

Email: Christophe Mathé - christophe.mathe@umontpellier.fr

*Corresponding author

Synthetic details and characterization data of new compounds

General

^{1}H, ^{31}P and ^{13}C NMR spectra were recorded at ambient temperature on a Bruker Avance III HD 400 MHz. Chemical shifts (δ) are quoted in parts per million (ppm) referenced to the residual solvent peak, ($CDCl_3$ fixed at 7.26 ppm and 77.16 ppm) relative to tetramethylsilane (TMS). Coupling constants, *J*, are reported in hertz. ESI mass and high resolution mass spectra (HRMS) were recorded in the positive or negative ion mode on a Micromass Q-TOF. Thin layer chromatography was performed on precoated aluminum sheets of Silica 60 F254 (Merck, Art. 5554), visualization of products being accomplished by UV absorbance and by charring with anisaldehyde solution, Dittmer reagent or $KMnO_4$ (solution in ethanol) and heating. Chromatography was performed on Merck Silica gel 60 (230–400 mesh ASTM).

(+/−)-4-*tert*-Butyldimethylsilyloxy-2-cyclopentenone ((+/−)-1)

Compound (+/−)-**1** was obtained in two steps from commercially available furfuryl alcohol following a reported procedure.

Rf (CH_2Cl_2): 0.63. ^{1}H NMR ($CDCl_3$, 300 MHz): δ = 7.44 (dd, *J* = 2.2, 5.6 Hz, 1H, H_3), 6.17 (dd, *J* = 1.2, 5.6 Hz, 1H, H_2), 4.98 (m, 1H, H_4), 2.70 (dd, *J* = 5.9, 18.2 Hz, 1H, H_{5a}), 2.24 (dd, *J* = 2.2, 18.2 Hz, 1H, H_{5b}), 0.89 (s, 9H, *t*-Bu), 0.11 (s, 6H, $2xCH_3$). ^{13}C NMR ($CDCl_3$, 75 MHz): δ = 207.0 (C_1), 164.4 (C_3), 135.0 (C_2), 71.4 (C_4), 45.6 (C_5), 26.3 (*t*-Bu), 18.7(C), -4.0 ($2xCH_3$).

(+/−)-Dimethyl ((4-((*tert*-butyldimethylsilyl)oxy)-1-hydroxycyclopent-2-en-1-yl)methyl) phosphonate ((+/−)-2)

To a solution of dimethyl methylphosphonate (310 mg, 2.35 mmol) in THF (4 mL) at −78 °C was added dropwise a solution of *n*-BuLi (0.94 mL, 2.5 M in THF, 2.35 mmol). The mixture was then stirred at −78 °C for 20 min and a solution of (+/−)-**1** (500 mg, 2.35 mmol) in THF (3 mL) was added dropwise. The reaction mixture was stirred for 2 h at rt. The reaction was quenched by the addition of a saturated solution of ammonium chloride (10 mL). The aqueous phase was extracted with ether (2 x 10 mL), and the combined organic phases were dried ($MgSO_4$) and concentrated. The residue was purified by column chromatography (PE/EtOAc 100:0 to 0:100) to give (+/−)-**2** (500 mg, 63%) as a colorless oil.

Rf (EtOAc): 0.38. ^{1}H NMR ($CDCl_3$, 300 MHz): δ = 5.98 (dd, *J* = 0.9, 5.6 Hz, 1H, H_2), 5.82 (dd, *J* = 1.9, 5.6 Hz, 1H, H_3), 4.72-4.68 (m, 1H, H_4), 3.74 (d, *J* = 11.0 Hz, 3H, OCH_3), 3.73 (d, *J* = 11.0 Hz, 3H, OCH_3), 3.50 (s, 1H, OH), 2.53 (ddd, *J* = 0.5, 6.6, 13.6 Hz, 1H, H_{5a}), 2.12 (dd, *J* = 2.1, 17.6 Hz, 2H, H_6), 1.89 (ddd, *J* = 2.2, 4.4, 13.6 Hz, 1H, H_{5b}), 0.87 (s, 9H, *t*-Bu-), 0.06 (s, 6H, 2xCH_3). ^{31}P NMR ($CDCl_3$, 127 MHz): δ = 31.01. ^{13}C NMR ($CDCl_3$, 75 MHz): δ = 138.2 (d, *J* = 8.8 Hz, C_2), 135.9 (C_3), 80.2 (d, *J* = 4.8 Hz, C_1), 75.2 (C_4), 52.5 (t, *J* = 5.8 Hz, 2xOCH_3), 50.1 (d, *J* = 9.2 Hz, C_5), 36.1 (d, *J* = 136.8 Hz, C_6), 26.0 (*t*-Bu), 18.2 (C), -4.5 (2xCH_3). HRMS calculated for $C_{14}H_{28}O_4PSi$: 319.1494; found: 319.1494.

(+/−)-Dimethyl ((3-((*tert*-butyldimethylsilyl)oxy)-1-hydroxycyclopentyl)methyl)phosphonate ((+/−)-3)

To a solution of (+/−)-**2** (500 mg, 1.43 mmol) in methanol (3 mL) was added Pd/C (10%, 7 mg). The solution was stirred under hydrogen at atmospheric pressure and rt overnight. The reaction mixture was filtrated through Celite and the filtrate was evaporated. Compound (+/−)-**3** was obtained as a colorless oil (482 mg, 96%). Rf (DCM/MeOH: 95/5): 0.4. ^{1}H NMR ($CDCl_3$, 300 MHz): δ = 4.39-4.35 (m, 1H, H_3), 4.06 (s, 1H, OH), 3.74 (d, J = 10.9 Hz, 3H, OCH_3), 3.74 (d, J = 10.9 Hz, 3H, OCH_3), 2.15 (d, J = 18.3 Hz, 2H, C_6), 2.01-1.78 (m, 6H, $H_{carbocycle}$), 0.86 (s, 9H, *t*-Bu-), 0.05 (s, 6H, $2xCH_3$). ^{31}P NMR ($CDCl_3$, 127 MHz): δ = 31.44. ^{13}C NMR ($CDCl_3$, 75 MHz): δ = 79.9 (d, J = 4.4 Hz, C_1), 74.7 (C_3), 52.4 (d, J = 9.8 Hz, OCH_3), 52.3 (d, J = 9.8 Hz, OCH_3), 49.2 (d, J = 8.4 Hz, C_2), 39.6 (d, J = 8.6 Hz, C_5), 36.1 (d, J = 138.7 Hz, C_6), 34.6 (C_4), 25.9 (*t*-Bu), 18.1 (C_q), -4.7 (CH_3), -4.8 (CH_3). MS ESI-QTof>0 *m/z* 339.18 $[M+H]^+$. HRMS calculated for $C_{14}H_{32}O_5PSi$: 339.1757; found: 339.1760.

(+/−)-3-((*tert*-Butyldimethylsilyl)oxy)-1-((dimethoxyphosphoryl)methyl)cyclopentyl acetate ((+/−)-4)

To a solution of (+/−)-**3** (7.6 g, 22.45 mmol) in dry Et_2O (100 mL) at rt was successively added DMAP (263 mg, 2.16 mmol), triethylamine (4.54 mL, 32.53 mmol) and acetic anhydride (3.07 mL, 32.53 mmol). The reaction was allowed to stir for 13 d and then concentrated. The residue was diluted with ethyl acetate (100 mL) and extracted twice with saturated sodium bicarbonate solution (100 mL), followed by brine (100 mL). The organic layer was dried over $MgSO_4$ and evaporated under reduced pressure. The residue was purified by column chromatography (DCM/MeOH 100:0 to 98:2) to give

(+/−)-**4** (7.20 g, 88%) as a colorless oil. Rf (DCM/MeOH: 95/5): 0.28. ^{1}H NMR ($CDCl_3$, 300 MHz): δ = 4.28-4.20 (m, 1H, H_3), 3.71 (d, *J* = 10.9 Hz, 2xOCH_3), 2.67 (dd, *J* = 15.5, 18.9 Hz, 1H, H_6), 2.45 (dd, *J* = 15.5, 19.5 Hz, 1H, H_6), 2.37-2.14 (m, 3H, $H_{carbocycle}$), 1.99 (s, 3H, CH_3), 1.94-1.67 (m, 3H, $H_{carbocycle}$), 0.85 (s, 9H, *t*-Bu-), 0.01 (s, 6H, 2xCH_3).^{31}P NMR ($CDCl_3$, 127 MHz): δ = 29.16.^{13}C NMR ($CDCl_3$, 75 MHz): δ = 171.2 (C=O), 85.8 (d, *J* = 4.7 Hz, C_1), 72.3 (C_3), 52.4 (d, *J* = 6.5 Hz,2xOCH_3), 48.3 (d, *J* = 8.5 Hz, C_2), 37.6 (d, J = 5.9 Hz, C_5), 34.6 (C_4), 32.8 (d, *J* = 138.8 Hz, C_6), 25.9 (*t*-Bu), 22.3 (CH_3), 18.1 (CH_3), -4.7 (CH_3), -4.7 (CH_3). MS ESI-QTof>0 *m/z* 381.19 $[M+H]^+$. HRMS calculated for $C_{16}H_{34}O_6PSi$: 381.1862; found: 381.1862.

(+/−)-1-((Dimethoxyphosphoryl)methyl)-3-hydroxycyclopentyl acetate ((+/−)-5)

To a solution of (+/−)-**4** (870 mg, 2.28 mmol) in THF (10 mL) at 0 °C was added slowly a solution of TBAF (1 M) in THF (2.28 mL, 2.28 mmol). The reaction mixture was stirred at 0 °C for 2 h and evaporated. Purification of the residue by column chromatography (DCM/MeOH 1:0 to 96:4) gave (+/−)-**5** (517 mg, 85%) as a colorless oil. Rf (DCM/MeOH: 95/5): 0.2. ^{1}H NMR ($CDCl_3$, 300 MHz): δ = 4.36-4.30 (m, 1H, H_3), 3.71 (d, *J* = 11.0 Hz, 2xOCH_3), 2.61 (dd, *J* = 12.1, 15.7 Hz, 1H, H_{6a}), 2.51 (dd, *J* = 12.1, 15.9 Hz, 1H, H_{6b}), 2.46-2.42 (m, 2H, $H_{carbocycle}$), 2.20 (dd, *J* = 6.7, 15.3 Hz, 1H, $H_{carbocycle}$), 2.01 (s, 3H, CH_3), 1.99-1.85 (m, 2H, $H_{carbocycle}$), 1.82-1.73 (m, 1H, $H_{carbocycle}$). ^{31}P NMR ($CDCl_3$, 127 MHz): δ = 28.88. ^{13}C NMR ($CDCl_3$, 75 MHz): δ = 171.1 (C=O), 85.7 (d, *J* = 5.0 Hz, C_1), 72.3 (C_3), 52.5 (d, *J* = 6.6 Hz, 2xOCH_3), 48.0 (d, *J* = 7.1 Hz, C_2), 37.3 (d, *J* = 8.0 Hz, C_5), 34.1 (C_4), 32.7 (d, *J* = 139.4 Hz, C_6), 22.3 (CH_3). MS ESI-QTof>0 *m/z* 267.1 $[M+H]^+$. HRMS calculated for $C_{10}H_{20}O_6P$: 267.0997; found: 267.0996.

(+/−)-4-((*tert*-Butyldimethylsilyl)oxy)-1-((dimethoxyphosphoryl)methyl)cyclopent-2-en-1-yl acetate ((+/−)-6)

To a solution of (+/−)-**2** (2 g, 5.9 mmol) in dry Et_2O (20 mL) at rt was added DMAP (72 mg, 0.59 mmol) and triethylamine (1.23 mL, 8.85 mmol). After stirring for 10 min, acetic anhydride (0.67 mL, 7.13 mmol) was added and the reaction was allowed to stir overnight and then concentrated. The residue was diluted in ethyl acetate (50 mL) and extracted twice with saturated sodium bicarbonate solution (50 mL) and brine (50 mL). The organic layer was dried over $MgSO_4$ and evaporated. The residue was purified by column chromatography ((EP/EtOAc 8:2 to 0:1)) to give (+/−)-**6** (1.96 g, 88%) as a colorless oil. Rf (EtOAc): 0.52. ^{1}H NMR ($CDCl_3$, 300 MHz): δ = 6.29 (dd, *J* = 1.1, 5.6 Hz, 1H, H_2), 5.91 (dd, *J* = 1.9, 5.6 Hz, 1H, H_3), 4.78-4.73 (m, 1H, H_4), 3.70 (d, *J* = 11.0 Hz, 6H, 2xOCH_3), 2.90 (dd, *J* = 6.9, 14.2 Hz, 1H, H_{5a}), 2.60 (dd, *J* = 12.9, 16.5 Hz, 1H, H_{6a}), 2.50 (dd, *J* = 12.9, 16.4 Hz, 1H, H_{6b}), 2.06 (ddd, *J* = 2.7, 4.8, 14.3 Hz, 1H, H_{5b}), 2.10 (s, 3H, CH_3), 0.88 (s, 9H, *t*-Bu), 0.07 (s, 3H, CH_3), 0.07 (s, 3H, CH_3). ^{31}P NMR ($CDCl_3$, 127 MHz): δ = 28.39. ^{13}C NMR ($CDCl_3$, 75 MHz): δ = 170.7 (C=O), 137.9 (C_3), 134.7 (d, *J* = 6.4 Hz, C_2), 87.3 (d, *J* = 4.0 Hz, C_1), 74.7 (C_4), 52.5 (d, *J* = 11.2 Hz, OCH_3), 52.4 (d, *J* = 11.2 Hz, OCH_3), 48.8 (d, *J* = 7.1 Hz, C_5), 33.0 (d, *J* = 138.7 Hz, C_6), 26.0 (*t*-Bu), 22.0 (CH_3), 18.3 (C), -4.5 (CH_3), -4.6 (CH_3).

(+/−)-1-((Dimethoxyphosphoryl)methyl)-4-hydroxycyclopent-2-en-1-yl acetate ((+/−)-7)

To a solution of (+/−)-**6** (1.7 g, 4.5 mmol) in THF (30 mL) at 0 °C was slowly added a solution of TBAF (1 M) in THF (5.4 mL, 5.4 mmol). The reaction mixture was stirred at 0 °C for 2 h then the solvent was evaporated. Purification of the residue by column

chromatography (DCM/MeOH 100:0 to 97:3) gave (+/−)-**7** (0.9 g, 76%) as a colorless oil. Rf (DCM/MeOH: 95/5): 0.2. ^{1}H NMR ($CDCl_3$, 300 MHz): δ = 6.20 (d, *J* = 5.6 Hz, 1H, H_2), 6.07 (dd, *J* = 2.2, 5.6 Hz, 1H, H_3), 4.74 (s, 1H, H_4), 3.70 (d, *J* = 11.0 Hz, 6H, 2xOCH_3), 2.90 (s, 1H, OH), 2.80 (dd, *J* = 7.2, 15.2 Hz, 1H, H_{5a}), 2.51 (dd, *J* = 15.5, 18.5 Hz, 1H, H_{6a}), 2.40 (dd, *J* = 15.5, 19.2 Hz, 1H, H_{6b}), 2.20 (ddd, *J* = 1.3, 3.1, 15.3 Hz, 1H, H_{5b}), 2.01 (s, 3H, CH_3). ^{31}P NMR ($CDCl_3$, 127 MHz): δ = 28.10. ^{13}C NMR ($CDCl_3$, 75 MHz): δ = 170.7 (C=O), 138.1 (C_3), 135.2 (d, *J* = 8.0 Hz, C_2), 88.1 (d, *J* = 3.8 Hz, C_1), 74.9 (C_4), 52.5 (d, *J* = 9.4 Hz, OCH_3), 52.4 (d, *J* = 9.3 Hz, OCH_3), 47.1 (d, *J* = 6.0 Hz, C_5), 34.2 (d, *J* = 139.8 Hz, C_6), 22.2 (CH_3). MS ESI-QTof>0 *m/z* 287.07 $[M+Na]^+$. HRMS calculated for $C_{10}H_{17}O_6NaP$: 287.0660; found: 287.0657.

(+/−)-1-((Dimethoxyphosphoryl)methyl)-3-(6-(bis-Boc-amino)-9*H*-purin-9-yl)cyclopentyl acetate ((+/−)-8)

To a solution of triphenylphosphine (2.16 g, 8.26 mmol) and bis-Boc-adenine (2.77 g, 8.26 mmol) in THF (20 mL) at 0 °C was added dropwise DIAD (1.62 mL, 8.26 mmol). The reaction mixture was stirred at rt for 1 h. A solution of (+/−)-**5** (1 g, 3.75 mmol) in THF (10 mL) was added to the reaction mixture. The reaction was stirred for 1 h before evaporation of solvents. Purification by flash chromatography (DCM/MeOH 100:0 to 95:5) gave (+/−)-**8** (927 mg, 42%) as a white foam. Rf (DCM/MeOH: 95/5): 0.26. ^{1}H NMR ($CDCl_3$, 300 MHz): δ = 8.83 (s, 1H, H_2), 8.12 (s, 1H, H_8), 5.18-5.07 (m, 1H, $H_{3'}$), 3.74 (d, *J* = 11.3 Hz, 2xOCH_3), 3.74 (d, *J* = 10.6 Hz, 2xOCH_3), 3.02 (dd, *J* = 8.7, 15.0 Hz, 1H, $H_{carbocycle}$), 2.92 (dd, *J* = 15.6, 18.9 Hz, 1H, $H_{6'a}$), 2.69 (dd, *J* = 15.6, 19.7 Hz, 1H, $H_{6'b}$), 2.59-2.41 (m, 4H, $H_{carbocycle}$), 2.30-2.15 (m, 1H, $H_{carbocycle}$), 2.08 (s, 3H, CH_3), 1.46 (s, 18H, 2x*t*-Bu). ^{31}P NMR ($CDCl_3$, 127 MHz): δ = 28.28 ppm. ^{13}C NMR ($CDCl_3$, 75

MHz): δ = 171.0 (C=O), 153.3 (C_6), 151.9 (C_2), 150.7 (COO*t*-Bu), 150.6 (C_4), 143.8 (C8), 129.7 (C_5), 86.3 (d, J = 4.5 Hz, $C_{1'}$), 83.9 (OC*t*-Bu), 55.09 ($C_{3'}$), 52.6 (d, J = 6.6 Hz, 2xOCH_3), 44.1 (d, J = 5.8 Hz, $C_{2'}$), 37.1 (d, J = 8.9 Hz, $C_{5'}$), 32.0 (d, J = 139.6 Hz, $C_{6'}$), 30.4 ($C_{4'}$), 28.0 (6xCH_3), 22.3 (CH_3). UV (EtOH) λ_{max} = 269 nm (ε_{max} = 15900). MS ESI-QTof>0 *m/z* 584.25 $[M+H]^+$. HRMS calculated for $C_{25}H_{39}N_5O_9P$: 584.2485; found: 584.2482.

(+/−)-3-(2-Amino-6-chloro-9*H*-purin-9-yl)-1-((dimethoxyphosphoryl)methyl)cyclo-pentyl acetate ((+/−)-9)

To a solution of triphenylphosphine (1.08 mg, 4.13 mmol), and 2-amino-6-chloropurine (700 mg, 4.13 mmol) in THF (10 mL) at 0 °C was added dropwise DIAD (0.813 mL, 4.13 mmol). The reaction mixture was stirred at rt for 1 h. Then a solution of (+/−)-**5** (500 mg, 1.87 mmol) in THF (6 mL) was added to the reaction mixture. The reaction was stirred at rt for 1 h before filtration and evaporation of the solvent. Purification by flash chromatography (DCM/MeOH 1:0 to 97:3) gave (+/−)-**9** (406 mg, 54%) as a white foam. Rf (DCM/MeOH: 95/5): 0.14. ^1H NMR ($CDCl_3$, 300 MHz): δ = 7.79 (s, 1H, H_8), 5.26 (s, 2H, NH_2), 4.96-4.85 (m, 1H, $H_{1'}$), 3.74(d, J = 11.0 Hz, 3H, OCH_3), 3.73 (d, J = 11.0 Hz, 3H, OCH_3), 2.95-2.73 (m, 3H), 2.57-2.45 (m, 3H), 2.43-2.28 (m, 1H, $H_{carbocycle}$), 2.21-2.12 (m, 1H, $H_{carbocycle}$), 2.07 (s, 3H, CH_3). ^{31}P NMR ($CDCl_3$, 127 MHz): δ = 28.58. ^{13}C NMR ($CDCl_3$, 75 MHz): δ = 170.9 (C=O), 158.7 (C_2), 153.5 (C_4), 151.6 (C_6), 141.43 (C_8), 125.1 (C_5), 86.3 (d, J = 4.6 Hz, $C_{1'}$), 54.6 ($C_{3'}$), 52.6 (d, J= 5.0 Hz, 2xOCH_3), 43.5 (d, J = 3.7 Hz, $C_{2'}$), 37.3 (d, J = 9.1 Hz, $C_{5'}$), 32.0 (d, J = 139.7 Hz, $C_{6'}$), 30.3 ($C_{4'}$), 22.2 (CH_3).

UV (EtOH) λ_{max} = 310 nm (ε_{max} = 8000), λ_{max} = 249 nm (ε_{max2} = 6900). MS ESI-QTof>0 *m/z* 418.10 $[M+H]^+$ HRMS calculated for $C_{15}H_{22}N_5O_5PCl$: 418.1047; found: 418.1045.

(+/−)-Dimethyl ((3-(6-amino-9*H*-purin-9-yl)-1-hydroxycyclopentyl)methyl)phosphonate ((+/−)-10)

To a stirred solution of (+/−)-**8** (490 mg, 0.84 mmol) in $ClCH_2CH_2Cl$ (5 mL) was added dropwise TFA (2.45 mL) and the reaction mixture was stirred at rt for 4 h. The volatiles were removed under reduced pressure and the residue was purified by column chromatography (DCM/MeOH 100:0 to 95:5) to give a colorless foam. To a solution of the foam in MeOH (10 mL) was added K_2CO_3 (90 mg, 0.65 mmol). The reaction mixture was stirred at rt for 7 h before filtration and evaporation of the solvents. Purification by flash chromatography (DCM/MeOH 100:0 to 90:10) gave (+/−)-**10** (170 mg, 60%) as a white foam.

Rf (DCM/MeOH: 9/1): 0.19. ^1H NMR ($CDCl_3$, 300 MHz): δ = 8.08 (s, 1H, H_2), 8.01 (s, 1H, H_8), 5.05-4.94 (m, 1H, $H_{3'}$), 3.75 (d, *J* = 11.0 Hz, 2xOCH_3), 2.56-247 (m, 2H, $H_{carbocycle}$), 2.47 (d, *J* = 18.3 Hz, 2H, $H_{6'}$), 2.25-2.14 (m, 2H, $H_{carbocycle}$), 2.07-1.90 (m, 2H, $H_{carbocycle}$). ^{31}P NMR ($CDCl_3$, 127 MHz) δ = 33.68. ^{13}C NMR ($CDCl_3$, 75 MHz): δ = 155.0 (C_6), 151.8 (C_2), 148.4 (C_4), 140.1 (C_8), 118.4 (C_5), 77.5 (d, *J* = 6.1 Hz, $C_{1'}$), 54.2 ($C_{3'}$), 52.8 (d, *J* = 5.4 Hz, 2xOCH_3), 45.9 (d, *J = 5.9 Hz*, $C_{2'}$), 38.1 (d, *J* = 9.4 Hz, $C_{5'}$), 34.6 (d, *J* = 137.5 Hz, $C_{6'}$), 29.6 ($C_{4'}$). UV (EtOH) λ_{max} = 261 nm (ε_{max} = 13800). MS ESI-QTof>0 *m/z* 342.16 $[M+4H]^+$. HRMS calculated for $C_{13}H_{21}N_5O_4P$: 342.1582; found: 342.1574.

(+/−)-Dimethyl ((3-(2-amino-6-methoxy-9*H*-purin-9-yl)-1-hydroxycyclopentyl)meth-yl)phosphonate ((+/−)-11)

To a solution of (+/−)-**9** (500 mg, 1.19 mmol) in MeOH (15 mL) was added K_2CO_3 (579 mg, 4.18 mmol). The reaction mixture was stirred at rt for 5 h before filtration and evaporation of the solvent. Purification by column chromatography (DCM/MeOH 100:0 to 90:10) gave (+/−)-**11** (300 mg, 68%) as a white foam. Rf (DCM/MeOH: 95/5): 0.1. ^1H NMR (D_2O, 300 MHz): δ = 7.83 (s, 1H, H_8), 4.91-4.80 (m, 1H, $H_{3'}$), 3.97 (s, 3H, OCH_3), 3.75 (d, *J* = 11.0 Hz, 6H, 2xOCH_3), 2.52-2.36 (m, 2H, $H_{carbocycle}$), 2.45 (d, *J* = 18.0 Hz, 2H, $H_{6'a}$), 2.22-2.07 (m, 2H, $H_{carbocycle}$), 2.03-1.86 (m, 2H, $H_{carbocycle}$). ^{31}P NMR (D_2O, 127 MHz): δ = 33.70. ^{13}C NMR (D_2O, 75 MHz): δ = 161.17 (C_6),159.4 (C_2), 152.6 (C_4), 138.6 (C8), 113.9 (C_5), 77.6 (d, *J* = 6.0 Hz, $C_{1'}$), 54.2 ($C_{3'}$), 53.6 (OCH_3), 52.81 (d, *J* = 6.0 Hz, 2xOCH_3), 45.9 (d, *J* = 9.3 Hz, $C_{2'}$), 38.2 (d, *J = 9.4 Hz*, $C_{5'}$), 34.6 (d, *J* = 137.2 Hz, $C_{6'}$), 29.7 ($C_{4'}$). UV (EtOH) λ_{max} = 282 nm (ε_{max} = 8800), λ_{max} = 247 nm (ε_{max} = 7900). MS ESI-QTof>0 *m/z* 372.14 $[M+H]^+$. HRMS calculated for $C_{14}H_{23}N_5O_5P$: 372.1437; found: 372.1441.

(+/−)-Sodium hydrogen ((3-(6-amino-9*H*-purin-9-yl)-1-hydroxycyclopentyl)methyl) phosphonate ((+/−)-12)

To a solution of (+/−)-**10** (103 mg, 0.3 mmol) in acetonitrile/DMF (5 mL 4:1, v/v) was added sodium iodide (135 mg, 0.9 mmol). The reaction mixture was heated to 40 °C and TMSCl (0.12 mL, 0.9 mmol) was added. The reaction was stirred for 1 h at 40 °C and overnight at rt. The reaction mixture was neutralized with TEAB (1 M). The solvent was evaporated and the residue was purified by reversed phase chromatography

(H_2O/MeOH 1:0 to 4:1) followed by dowex chromatography (Na^+) to give (+/−)-**12** (62 mg, 61%) as a white solid. Rf (*i*PrOH/NH_4OH/H_2O: 7/2/1): 0.26. ^1H NMR (D_2O, 300 MHz): δ = 8.21 (s, 1H, H_2), 8.10 (s, 1H, H_8), 5.11-4.96 (m, 1H, $H_{3'}$), 2. 68-2.38 (m, 2H, $H_{carbocycle}$), 2.32-2.12 (m, 2H, $H_{carbocycle}$), 2.00 (d, *j = 16.1 Hz*, 2H, $H_{6'a,b}$), 1.95 (dd, *J* = 7.2, 14.2 Hz, 2H, $H_{carbocycle}$). ^{31}P NMR (D_2O, 127 MHz): δ = 18.83. ^{13}C NMR ($CDCl_3$, 75 MHz) : δ = 155.2 (C_6), 151.9 (C_2), 148.7 (C_4), 140.5 (C8), 118.5 (C_5), 79.1 (d, *J*= 4.7 Hz, $C_{1'}$), 54.5 ($C_{3'}$), 46.0 (d, *J* = 7.6 Hz, $C_{2'}$), 38.7 (d, *J* = 125.4 Hz, $C_{6'}$), 38.2 (d, *J* = 8.8 Hz, $C_{5'}$), 30.2 ($C_{4'}$). **UV** (H_2O) λ_{max} = 261 nm (ε_{max} = 12700), λ_{min} = 230 nm (ε_{min} = 2300). MS ESI-QTof>0 *m/z* 314.10 $[M+H]^+$. HRMS calculated for $C_{11}H_{17}N_5O_4P$: 314.1018; found: 314.1014.

(+/−)-Sodium hydrogen ((3-(2-amino-6-oxo-1*H*-purin-9(6*H*)-yl)-1-hydroxycyclopent-yl)methyl)phosphonate ((+/−)-13)

To a solution of (+/−)-**11** (150 mg, 0.404 mmol) in DMF (3 mL) at 0 °C was added dropwise trimethylsilyl bromide (524 µL, 4.04 mmol). The reaction was stirred 1 h at 0 °C and 23 h at rt. After addition of TEAB (1 M) until pH 7, evaporation of solvents and purification by reversed phase chromatography (H_2O/MeOH 1:0 to 1:1) followed by Dowex chromatography (Na^+) compound (+/−)-**13** (50 mg, 35%) was obtained as a white solid. Rf (*i*PrOH/NH_4OH/H_2O: 6/3/1): 0.35. ^1H NMR (D_2O, 300 MHz): δ = 7.91 (s, 1H, H_8), 4.95-4.86 (m, 1H, $H_{3'}$), 2.51-2.39 (m, 2H, $H_{carbocycle}$), 2.26-2.15 (m, 2H, $H_{carbocycle}$), 2.02 (d, *J* = 16.4 Hz, 2H, $H_{6'}$), 1.97-190 (m, 2H, $H_{carbocycle}$). ^{31}P NMR (D_2O, 127 MHz): δ = 19.26. ^{13}C NMR (D_2O, 75 MHz): δ = 158.9 (C_6),153.4 (C_2), 151.4 (C_4), 138.1 (C_8), 116.0 (C_5), 79.1 (d, *J* = 4.8 Hz, $C_{1'}$), 54.0 ($C_{3'}$), 46.1 (d, *J* = 7.8 Hz, $C_{2'}$), 38.6 (d, *J* = 126.6 Hz,

$C_{6'}$), 38.2 (d, *J* = 8.8 Hz, $C_{5'}$), 30.2 ($C_{4'}$). UV (H_2O) λ_{max} = 253 nm (ε_{max} = 10800), λ_{min} = 227 nm (ε_{min} = 3200). MS ESI-QTof>0 *m/z* 330.10 $[M+H]^+$. HRMS calculated for $C_{11}H_{17}N_5O_5P$: 330.0967; found: 330.0968.

(+/−)-1-((Dimethoxyphosphoryl)methyl)-4-(6-(bis-boc-amino)-9*H*-purin-9-yl)cyclopent-2-en-1-yl acetate ((+/−)-14)

To a solution of triphenylphosphine (2.75 g, 10.5 mmol) and dried bis-Boc-adenine (3.5 g, 10.5 mmol) in THF (30 mL) at 0 °C was added dropwise DIAD (2.06 mL, 10.5 mmol). The reaction mixture was stirred at rt for 1 h after which a solution of (+/−)-**7** (1.28 g, 4.8 mmol) in THF (10 mL) was added. The mixture was stirred at rt for 1 h before evaporation of the solvents. The product was purified by silica gel column chromatography (DCM/MeOH 100:0 to 95:5) to give (+/−)-**14** (1.55 g, 56%) as a white foam. Rf (DCM/MeOH: 95/5): 0.36. ^{1}H NMR ($CDCl_3$, 300 MHz): δ = 8.84 (s, 1H, H_2), 8.20 (s, 1H, H_8), 6.65 (dd, *J* = 2.3, 5.6 Hz, 1H, $H_{2'}$), 6.13 (dd, *J* = 1.9, 5.6 Hz, 1H, $H_{3'}$), 6.00-5.96 (m, 1H, $H_{4'}$), 3.74 (d, *J* = 12.0 Hz, 3H, OCH_3), 3.73 (d, *J* = 12.0 Hz, 3H, OCH_3), 3.15 (dd, *J* = 7.6, 14.8 Hz, 1H, $H_{5'a}$), 2.82 (dd, *J* = 15.4, 18.4 Hz, 1H, $H_{6'a}$), 2.71 (dd, *J* = 15.4, 20.1 Hz, 1H, $H_{6'b}$), 2.57 (dd, *J* = 6.2, 14.8 Hz, 1H, $H_{5'b}$), 2.04 (s, 3H, CH_3), 1.45 (s, 18H, 2x*t*-Bu). ^{31}P NMR ($CDCl_3$, 127 MHz): δ = 27.48. ^{13}C NMR ($CDCl_3$, 75 MHz): δ = 170.5 (C=O), 153.3 (C_4), 152.1 (C_2), 150.7 (COO*t*-Bu), 150.5 (C_6), 143.4 (C_8), 138.2 (d, *J* = 9.4 Hz, $C_{2'}$), 134.5 ($C_{3'}$), 129.2 (C_5), 88.7 (d, *J* = 2.3 Hz, $C_{1'}$), 84.0 (OC*t*-Bu), 59.0 ($C_{4'}$), 52.7 (d, *J* = 10.4 Hz, OCH_3), 52.6 (d, *J* = 10.4 Hz, OCH_3), 45.0 (d, *J* = 3.5 Hz, $C_{5'}$), 32.7 (d, *J* = 139.4 Hz, $C_{6'}$), 27.9 (2x*t*-Bu), 21.9 (CH_3). UV (EtOH) λ_{max}= 268 nm (ε_{max} =

11500). MS ESI-QTof>0 *m/z* 582.23 $[M+H]^+$. HRMS calculated for $C_{25}H_{37}N_5O_9P$: 582.2329; found: 582.2326.

(+/−)-Dimethyl ((4-(6-amino-9*H*-purin-9-yl)-3-hydroxycyclopent-1-en-1-yl)methyl) phosphonate ((+/−)-15)

To a stirred solution of (+/−)-**14** (1 g, 1.72 mmol) in $ClCH_2CH_2Cl$ (10 mL) was added dropwise TFA (5 mL) and the reaction was stirred at rt for 3 h. The volatiles were removed under reduced pressure and the residue was purified by flash chromatography (DCM/MeOH 1:0 to 9:1) to give (+/−)-**15** (497 mg, 85%) as a white foam. Rf (DCM/MeOH: 9/1): 0.41. ^1H NMR (D_2O, 300 MHz): δ = 8.08 (s, 1H, H_2), 8.06 (s, 1H, H_8), 6.07-5.73 (m, 1H, $H_{2'}$), 5.09 (t, J = 4.4 Hz, $H_{3'}$), 4.89-4.83 (m, 1H, $H_{4'}$), 3.80 (d, J = 11.1 Hz, $2xOCH_3$), 3.19-2.93 (m, 3H, $H_{6'}$ + $H_{5'a}$), 2.84-2.75 (m, 1H, $H_{5'b}$). ^{31}P NMR (D_2O, 127 MHz): δ = 32.02. ^{13}C NMR (D_2O, 75 MHz): δ = 154.9 (C_4), 151.6 (C_2), 148.4 (C_6), 140.6 (C_8), 135.9 (d, J = 11.3 Hz, $C_{1'}$), 130.0 (d, J = 12.1 Hz, $C_{2'}$), 118.4 (C_5), 80.6 (d, J = 2.4 Hz, $C_{3'}$), 62.6 ($C_{4'}$), 53.4 (d, J = 6.6 Hz, $2xOCH_3$), 39.8 (d, J = 3.6 Hz, $C_{5'}$), 26.1 (d, J = 137.1 Hz, $C_{6'}$). UV (EtOH) λ_{max} = 261 nm (ε_{max} = 14800). MS ESI-QTof>0 *m/z* 340.12 $[M+H]^+$. HRMS calculated for $C_{13}H_{19}N_5O_4P$: 340.1175; found: 340.1176.

(+/−)-Sodium hydrogen ((4-(6-amino-9*H*-purin-9-yl)-3-hydroxycyclopent-1-en-1-yl)methyl)phosphonate ((+/−)-16)

To a solution of (+/−)-**15** (260 mg, 0.76 mmol) in acetonitrile (4 mL) was added sodium iodide (344 mg, 2.29 mmol) and the mixture was heated to 40 °C. TMSCl (290 µL, 2.29 mmol) was added and the reaction mixture was stirred for 1 h at 40 °C and overnight at

rt. After neutralization with TEAB (1 M) and the solvent was evaporated. The residue was purified by reversed phase chromatography (H_2O/MeOH 1:0 to 4:1) followed by dowex chromatography (Na^+) to give (+/−)-**16** (160 mg, 60%) as a white solid. Rf (*i*-PrOH/NH_4OH/H_2O: 7/2/1): 0.28. ^1H NMR (D_2O, 300 MHz): δ = 8.17 (s, 1H, H_2), 8.12 (s, 1H, H_8), 5.64 (d, J = 2.4 Hz, 1H, $H_{3'}$), 5.05 (s, 1H, $H_{2'}$), 4.95-4.84 (m, 1H, $H_{4'}$), 3.23 (dd, J = 7.8, 17.0 Hz, 1H, $H_{5'a}$), 2.85 (d, J = 17.2 Hz, 1H, $H_{5'b}$), 2.56 (d, J = 20.4 Hz, 2H, $H_{6'}$). ^{31}P NMR (D_2O, 127 MHz): δ = 17.23. ^{13}C NMR (D_2O, 75 MHz): δ = 155.3 (C_4), 152.0 (C_2), 148.6 (C_6), 143.0 (d, J = 9.7 Hz, $C_{1'}$), 141.0 (C_8), 125.6 (d, J = 10.5 Hz, $C_{2'}$), 118.6 (C_5), 81.5 (d, J = 2.4 Hz, $C_{3'}$), 62.8 ($C_{4'}$), 40.3 (d, J = 2.5 Hz, $C_{5'}$), 32.5 (d, J = 124.6 Hz, $C_{6'}$). UV (H_2O) λ_{max} = 260 nm (ε_{max} = 13600). MS ESI>0 *m/z* 312.08 $[M+H]^+$. HRMS calculated for $C_{11}H_{15}N_5O_4P$: 312.0862; found: 312.0861.

2-(bis-Boc-amino)-6-methoxy-purine (17)

To a stirred suspension of 2-amino-6-methoxypurine (3 g, 18.2 mmol) and DMAP (670 mg, 5.45 mmol) in THF (50 mL) was added Boc_2O (15.9 g, 72.7 mL). The reaction mixture was stirred for 3 h at rt. Then the solvent was removed under reduced pressure and the residue was dissolved in methanol (100 mL) and a saturated solution of $NaHCO_3$ (50 mL). The mixture was heated at 50 °C for 1 h and the solvent was removed under reduced pressure. The aqueous layer was extracted with $CHCl_3$ (3 × 100 mL).The organic layer was dried over $MgSO_4$ and filtered. The solvent was removed under reduced pressure and the residue was purified by silica gel column chromatography (PE/EtOAc 8:2 to 0:1) to give **17** (5 g, 75%). Rf (EtOAc): 0.63. ^1H NMR ($CDCl_3$, 300 MHz): δ = 13.09 (s, 1H, NH), 8.17 (s, 1H, H_8), 4.14 (s, 3H, OCH_3), 1.50 (s, 18H, 2x*t*-Bu).

^{13}C NMR ($CDCl_3$, 75 MHz): δ = 161.4, 153.2, 151.5, 151.0, 142.2, 119.5 (C_5), 84.0 (OC*t*-Bu), 54.7 (OCH_3), 28.0 (2x*t*-Bu).

(+/−)-1-((Dimethoxyphosphoryl)methyl)-4-(2-(bis-Boc-amino)-6-methoxy-9*H*-purin-9-yl)cyclopent-2-en-1-yl acetate ((+/−)-18)

To a solution of triphenylphosphine (1.9 g, 7.24 mmol) and 2-(bis-Boc-amino)-6-methoxypurine (**17**, 2.6 g, 7.24 mmol) in THF (20 mL) was added dropwise DIAD (1.4 mL, 7.24 mmol) at 0 °C. The reaction mixture was stirred at rt for 1 h after which a solution of (+/−)-**7** (870 mg, 3.4 mmol) in THF (5 mL) was added. The mixture was stirred at rt for 1 h before evaporation of the solvents. The residue was purified by flash chromatography (EtOAc 100% and DCM/MeOH 99:1) to give (+/−)-**18** (1.1 g, 55%) and (+/−)-**19** (100 mg, 5%) as white foam. Rf (DCM/MeOH: 95/5): 0.29. ^1H NMR ($CDCl_3$, 400 MHz): δ = 8.03 (s, 1H, H_8), 6.63 (dd, *J* = 2.3, 5.6 Hz, 1H, $H_{2'}$), 6.11 (dd, *J* = 1.9, 5.6 Hz, 1H, $H_{3'}$), 5.99-5.77 (m, 1H, $H_{4'}$), 4.14 (s, 3H, OCH_3), 3.74 (d, *J* = 11.0 Hz, 3H, OCH_3), 3.73 (d, *J* = 11.0 reHz, 3H, OCH_3), 3.12 (dd, *J* = 7.6, 14.8 Hz, 1H, $H_{5'a}$), 2.84 (dd, *J* = 15.4, 18.5 Hz, 1H, $H_{6'a}$), 2.71 (dd, *J* = 15.4, 20.1 Hz, 1H, $H_{6'b}$), 2.52 (dd, *J* = 6.2, 14.8 Hz, 1H, $H_{5'b}$), 2.03 (s, 3H, CH_3), 1.45 (s, 18H, 6xCH_3). ^{31}P NMR ($CDCl_3$, 162 MHz): δ = 27.36. ^{13}C NMR ($CDCl_3$, 100 MHz): δ = 170.6 (C=O), 161.6 (C_6), 152.6 (C_4), 152.0 (COO*t*-Bu), 151.1(C_2), 141.4 (C_8), 138.1 (d, *J* = 9.1 Hz, $C_{2'}$), 134.6 ($C_{3'}$), 120.3 (C_5), 88.8 (d, *J* = 1.9 Hz, $C_{1'}$), 83.2 (OC*t*-Bu), 58.9 ($C_{4'}$), 54.7 (OCH_3), 52.6 (d, *J* = 15.3 Hz, OCH_3), 52.6 (d, *J* = 15.4 Hz, OCH_3), 45.1 (d, *J* = 3.5 Hz, $C_{5'}$), 32.6 (d, *J* = 139.2 Hz, $C_{6'}$), 28.0 (6xCH_3), 21.9 (CH_3). UV (EtOH) λ_{max} = 253 nm (ε_{max} = 17100). MS ESI-QTof>0 *m/z* 612.24 $[M+H]^+$. HRMS calculated for $C_{26}H_{39}N_5O_{10}P$: 612.2435; found: 612.2433.

(+/−)-1-((Dimethoxyphosphoryl)methyl)-4-(2-(bis-boc-amino)-6-methoxy-7*H*-purin-7-yl)cyclopent-2-en-1-yl acetate ((+/−)-19)

Rf (DCM/MeOH: 95/5): 0.2. ^{1}H NMR ($CDCl_3$, 400 MHz): δ = 8.12 (s, 1H, H_8), 6.68 (dd, *J* = 2.2, 5.6 Hz, 1H, $H_{2'}$), 6.20 (dd, *J* = 1.9, 5.6 Hz, 1H, $H_{3'}$), 6.01-5.98 (m, 1H, $H_{4'}$), 4.13 (s, 3H, OCH_3), 3.72 (d, *J* = 11.0 Hz, 6H, 2xOCH_3), 3.15 (dd, *J* = 7.5, 15.0 Hz, 1H, $H_{5'a}$), 2.79 (dd, *J* = 15.5, 18.5 Hz, 1H, $H_{6'a}$), 2.54 (dd, *J* = 15.4, 20.0 Hz, 1H, $H_{6'b}$), 2.38 (dd, *J* = 5.6, 15.0 Hz, 1H, $H_{5'b}$), 2.06 (s, 3H, CH_3), 1.44 (s, 18H, 6xCH_3). ^{31}P NMR ($CDCl_3$, 162 MHz): δ = 26.97. ^{13}C NMR ($CDCl_3$, 100 MHz): δ = 170.5 (C=O), 163.0 (C_4), 157.6 (C_6), 152.0 (COO*t*-Bu), 151.3 (C_2), 144.0 (C_8), 138.9 (d, *J* = 9.7 Hz, $C_{2'}$), 133.8 ($C_{3'}$), 111.4 (C_5), 88.7 (d, *J* = 2.9 Hz, $C_{1'}$), 83.1 (OC*t*-Bu), 62.2 ($C_{4'}$), 54.7 (OCH_3), 52.7 (d, *J* = 10.3 Hz, OCH_3), 52.6 (d, *J* = 10.3 Hz, OCH_3), 46.7 (d, *J* = 3.8 Hz, $C_{5'}$), 33.3 (d, *J* = 139.4 Hz, $C_{6'}$), 28.1 (6xCH_3), 21.9 (CH_3). MS ESI-QTof>0 *m/z* 612.24 $[M+H]^+$. HRMS calculated for $C_{26}H_{39}N_5O_{10}P$: 612.2435; found: 612.2437.

(+/−)-Dimethyl ((4-(2-amino-6-methoxy-9*H*-purin-9-yl)-3-hydroxycyclopent-1-en-1-yl)methyl) phosphonate ((+/−)-20)

To a stirred solution of (+/−)-**18** (780 mg, 1.27 mmol) in $ClCH_2CH_2Cl$ (10 mL) was added dropwise TFA (3.7 mL) and the reaction was stirred at rt for 7 h. The volatile solvents were removed under reduced pressure and the residue was purified by flash chromatography (DCM/MeOH 100:0 to 96:4) to give (+/−)-**20** (240 mg, 51%) as a white foam. Rf (DCM/MeOH: 95/5): 0.07. ^{1}H NMR (MeOD, 300 MHz): δ = 7.86 (s, 1H, H_8), 5.79-5.77 (m, 1H, $H_{2'}$), 5.14-5.10 (m, 1H, $H_{3'}$), 4.78-4.71 (m, 1H, $H_{4'}$), 4.04 (s, 3H, OCH_3), 3.77 (d, *J* = 11.0 Hz, 6H, 2xOCH_3), 3.04-2.80 (m, 4H, $H_{5'}$ + $H_{6'}$). ^{31}P NMR

(MeOD, 127 MHz): δ = 29.59. ^{13}C NMR (MeOD, 75 MHz): δ = 162.7 (C_6),161.6 (C_2), 155.1 (C_4), 139.8 (C8) , 136.4 (d, *J* = 11.0 Hz, $C_{1'}$), 132.2 (d, *J* = 12.2 Hz, $C_{2'}$), 115.6 (C_5), 81.7 ($C_{3'}$), 64.8 ($C_{4'}$), 54.1 (OCH3), 53.6 (d, *J* = 6.7 Hz, 2xOCH3), 40.7 (d, *J* = 2.8 Hz, $C_{5'}$), 28.2 (d, *J* = 139.3 Hz, $C_{6'}$). UV (EtOH) λ_{max} = 279 nm (ε_{max} = 7000), λ_{max} = 250 nm (ε_{max} = 5800). MS ESI-QTof>0 *m/z* 370.13 $[M+H]^+$. HRMS calculated for $C_{14}H_{21}N_5O_5P$: 370.1280; found: 370.1280.

(+/−)-Sodium hydrogen ((4-(2-amino-6-oxo-1*H*-purin-9(6*H*)-yl)-3-hydroxycyclopent-1-en-1-yl)methyl)phosphonate ((+/−)-21)

To a solution of (+/−)-**20** (90 mg, 0.24 mmol) in DMF (2 mL) at 0 °C was added dropwise trimethylsilyl bromide (310 µL, 2.43 mmol). The reaction was stirred for 1 h at 0 °C and 15 h at rt. After the addition of TEAB until pH 7, the solvents were evaporatd and the residue purified by reversed phase chromatography (H_2O/MeOH 1:0 to 1:1) followed by Dowex chromatography (Na^+) to give (+/−)-**21** (50 mg, 59%) as a white solid. Rf (*i*-PrOH/NH_4OH/H_2O: 10/9/1): 0.65. ^1H NMR (D_2O, 300 MHz): δ = 7.83 (s, 1H, H_8), 6.65 (d, *J* = 2.8 Hz, 1H, $H_{2'}$), 4.95 (s, 1H, $H_{3'}$), 4.74-4.68 (m, 1H, $H_{4'}$), 3.16 (dd, *J* = 8.8, 16.2 Hz, 1H, $H_{5'a}$), 2.80 (d, *J* = 17.1 Hz, 1H, $H_{5'b}$), 2.62 (d, *J* = 20.1 Hz, 2H, $H_{6'}$). ^{31}P NMR (D_2O, 127 MHz): δ = 18.57. ^{13}C NMR (D_2O, 75 MHz): δ = 158.8 (C_6),153.4 (C_2), 151.3 (C_4), 141.5 (d, *J* = 10.1 Hz, $C_{1'}$), 138.2 (C_8), 126.4 (d, *J* = 10.8 Hz, $C_{2'}$), 115.9 (C_5), 81.6 ($C_{3'}$), 62.2 ($C_{4'}$), 40.2 (d, *J* = 1.0 Hz, $C_{5'}$), 31.8 (d, *J* = 127.5 Hz, $C_{6'}$). UV (H_2O) λ_{max} = 253 nm (ε_{max} = 9800). MS ESI-QTof<0 *m/z* 326.07 $[M-H]^-$ .HRMS calculated for $C_{11}H_{13}N_5O_5P$: 326.0654; found: 326.0655.

(+/−)-4-(6-Benzamido-9*H*-purin-9-yl)-1-((dimethoxyphosphoryl)methyl)cyclopent-2-en-1-yl acetate ((+/−)-22)

To a solution of triphenylphosphine (1.96 g, 7.5 mmol) and dried N^6-Bz-adenine (1.8 g, 7.5 mmol) in THF (20 mL) at 0 °C was added dropwise DIAD (1.47 mL, 7.5 mmol). The reaction mixture was stirred at rt for 1 h. Then a solution of (+/−)-**7** (900 mg, 3.4 mmol) in THF (4 mL) was added and the reaction mixture was stirred at rt for 1 h followed by evaporation of the solvents. The product was purified by flash chromatography (EtOAc 100% and DCM/MeOH 100:0 to 95:5) to give (+/−)-**22** (265 mg, 17%) as a white foam. Rf (DCM/MeOH: 95/5): 0.25. ^{1}H NMR ($CDCl_3$, 300 MHz): δ = 9.14 (s, 1H, NH), 8.79 (s, 1H, H_2), 8.15 (s, 1H, H_8), 8.03-7.49 (m, 5H, H_{Ar}), 6.66 (dd, J = 2.3, 5.6 Hz, 1H, $H_{2'}$), 6.16 (dd, J = 1.9, 5.6 Hz, 1H, $H_{3'}$), 6.08-5.93 (m, 1H, $H_{4'}$), 3.75 (d, J = 11.0 Hz, 3H, OCH_3), 3.74 (d, J = 11.0 Hz, 3H, OCH_3), 3.16 (dd, J = 7.6, 14.8 Hz, 1H, $H_{5'a}$), 2.87 (dd, J = 15.4, 18.5 Hz, H, $H_{6'a}$), 2.74 (dd, J = 15.4, 20.1 Hz, H, $H_{6'b}$), 2.59 (dd, J = 6.2, 14.8 Hz, 1H, $H_{5'b}$), 2.05 (s, 3H, CH_3). ^{31}P NMR ($CDCl_3$, 127 MHz): δ = 27.52. ^{13}C NMR ($CDCl_3$, 75 MHz): δ = 170.6 (C=O), 164.8 (C=O), 152.7 (C_2), 152.0 (C_4), 149.7 (C_6), 141.6 (C_8), 138.2 (d, J = 9.1 Hz, $C_{2'}$), 134.5 (CH_{Ar}), 133.8 (C_{Ar}), 132.9 ($C_{3'}$), 123.3 (C_5), 88.8 (d, J = 1.0 Hz, $C_{1'}$), 59.0 ($C_{4'}$), 52.7 (d, J = 10.7 Hz, OCH3), 52.6 (d, J = 10.7 Hz, OCH3), 45.0 (d, J = 2.3 Hz, $C_{5'}$), 32.7 (d, J = 139.2 Hz, $C_{6'}$), 21.9 (CH_3). UV (EtOH) λ_{max} = 280 nm (ε_{max} = 20500). MS ESI-QTof>0 *m/z* 486.15 $[M+H]^+$. HRMS calculated for $C_{22}H_{25}N_5O_6P$: 486.1542; found: 486.1537.

(+/−)-4-(2-Amino-6-chloro-9*H*-purin-9-yl)-1-((dimethoxyphosphoryl)methyl)cyclopent-2-en-1-yl acetate ((+/−)-23)

To a solution of triphenylphosphine (650 mg, 2.5 mmol) and 2-amino-6-chloropurine (420 mg, 2.5 mmol) in THF (10 mL) at 0 °C was added dropwise DIAD (0.5 mL, 2.5 mmol). The reaction mixture was stirred at rt for 1 h after which a solution of (+/−)-**7** (300 mg, 1.13 mmol) in THF (5 mL) was added. The reaction mixture was stirred at rt for 1 h before filtration and evaporation of the solvent. Purification by flash chromatography (DCM/MeOH 100:0 to 97:3) gave (+/−)-**23** (332 mg, 53%) as a white foam. Rf (DCM/MeOH: 95/5): 0.17. ^{1}H NMR ($CDCl_3$, 300 MHz): δ = 7.84 (s, 1H, H_8), 6.61 (dd, *J* = 2.3, 5.6 Hz, 1H, $H_{2'}$), 6.10 (dd, *J* = 1.9, 5.6 Hz, 1H, $H_{3'}$), 5.83-5.65 (m, 1H, $H_{4'}$), 5.16 (s, 2H, NH_2), 3.74 (d, *J* = 11.0 Hz, 3H, OCH_3), 3.73 (d, *J* = 11.0 Hz, 3H, OCH_3), 3.08 (dd, *J* = 7.6, 14.9 Hz, 1H, $H_{5'a}$), 2.86 (dd, *J* = 15.4, 18.5 Hz, 1H, $H_{6'a}$), 2.69 (dd, *J* = 15.4, 20.1 Hz, 1H, $H_{6'b}$), 2.49 (dd, *J* = 6.0, 14.9 Hz, 1H, $H_{5'b}$), 2.04 (s, 3H, CH_3). ^{31}P NMR ($CDCl_3$, 127 MHz): δ = 27.50. ^{13}C NMR ($CDCl_3$, 75 MHz): δ = 170.5 (C=O), 159.1 (C_2), 153.7 (C_4), 151.6 (C_6), 140.7 (C_8), 138.2 (d, *J* = 9.3 Hz, $C_{2'}$), 134.4 ($C_{3'}$), 125.7 (C_5), 88.8 (d, *J* = 2.6 Hz, $C_{1'}$), 58.7 ($C_{4'}$), 52.7 (d, *J* = 9.9 Hz, OCH_3), 52.6 (d, *J* = 9.8 Hz, OCH_3), 44.7 (d, *J* = 3.7 Hz, $C_{5'}$), 32.9 (d, *J* = 139.3 Hz, $C_{6'}$), 21.9 (CH_3). UV (EtOH) λ_{max} = 310 nm (ε_{max} = 9500). MS ESI-QTof>0 *m/z* 416.09 $[M+H]^+$. HRMS calculated for $C_{15}H_{20}N_5O_5PCl$: 416.0891; found: 416.0888.

(+/−)-Dimethyl ((4-(6-amino-9*H*-purin-9-yl)-1-hydroxycyclopent-2-en-1-yl)methyl)phosphonate ((+/−)-24)

To a solution of (+/−)-**22** (250 mg, 0.51 mmol) in MeOH (5 mL) was added K_2CO_3 (143 mg, 1.03 mmol) and the reaction mixture was stirred at rt for 20 h. After filtration and evaporation of the solvent the residue was purified by flash chromatography (DCM/MeOH 1:0 to 9:1) to give (+/−)-**24** (60 mg, 34%) as a white solid. Rf (DCM/MeOH: 9/1): 0.2. ^{1}H NMR (D_2O, 300 MHz): δ = 8.14 (s, 1H, H_2), 8.03 (s, 1H, H_8), 6.37 (dd, *J* = 1.5, 5.4 Hz, 1H, $H_{2'}$), 6.22 (dd, *J* = 1.7, 5.5 Hz, 1H, $H_{3'}$), 5.76-5.72 (m, 1H, $H_{4'}$), 3.71 (d, *J* = 11.0 Hz, 6H, 2xOCH_3), 2.77 (dd, *J* = 7.7, 14.6 Hz, 1H, $H_{5'a}$), 2.51 (dd, *J* = 5.0, 18.5 Hz, 2H, $H_{6'}$), 2.29 (dd, *J* = 5.3, 14.7 Hz, 1H, $H_{5'b}$). ^{31}P NMR (D_2O, 127 MHz): δ = 32.33. ^{13}C NMR (D_2O, 75 MHz): δ = 155.0 (C_2), 151.6 (C_4), 148.5 (C_6), 140.9 (d, *J* = 9.3 Hz, $C_{2'}$), 140.2 (C_8), 131.3 ($C_{3'}$), 118.6 (C_5), 80.9 (d, *J* = 4.1 Hz, $C_{1'}$), 59.3 ($C_{4'}$), 52.9 (t, *J* = 6.5 Hz, 2xOCH3), 46.0 (d, *J* = 6.5 Hz, $C_{5'}$), 34.9 (d, *J* = 137.3 Hz, $C_{6'}$). UV (H_2O) λ_{max} = 261 nm (ε_{max} = 12200). MS ESI-QTof>0 *m/z* 340.12 $[M+H]^+$. HRMS calculated for $C_{13}H_{19}N_5O_4P$: 340.1175; found: 340.1177

(+/−)-Dimethyl ((4-(2-amino-6-methoxy-9*H*-purin-9-yl)-1-hydroxycyclopent-2-en-1-yl)methyl)phosphonate ((+/−)-25)

To a solution of (+/−)-**23** (100 mg, 0.24 mmol) in MeOH (3 mL) was added K_2CO_3 (116 mg, 0.84 mmol) and the reaction mixture was stirred at rt for 4 h. After filtration and evaporation of the volatiles the residue was purified by flash chromatography (DCM/MeOH 100:0 to 90:10) to give (+/−)-**25** (64 mg, 72%) as a white foam. Rf (DCM/MeOH: 95/5): 0.1. ^{1}H NMR ($CDCl_3$, 300 MHz): δ = 7.55 (s, 1H, H_8), 6.28 (dd, *J* =

1.9, 5.5 Hz, 1H, $H_{2'}$), 6.10 (dd, *J* = 2.1, 5.5 Hz, 1H, $H_{3'}$), 5.74-5.69 (m, 1H, $H_{4'}$), 4.92 (s, 2H, NH_2), 4.06 (s, 3H, OCH_3), 3.77 (d, *J* = 13.6 Hz, 3H, OCH_3), 3.74 (d, *J* = 13.6 Hz, 3H, OCH_3), 2.81 (dd, *J* = 7.8, 14.3 Hz, 1H, $H_{5'a}$), 2.36 (dd, *J* = 1.0, 17.6 Hz, 2H, $H_{6'}$), 2.22-2.15 (m, 1H, $H_{5'b}$). ^{31}P NMR ($CDCl_3$, 127 MHz): δ = 30.91. ^{13}C NMR ($CDCl_3$, 75 MHz): δ = 161.8 (C_6),159.4 (C_2), 153.73 (C_4), 141.1 (d, *J* = 10.7 Hz, $C_{2'}$), 137.3 (C_8), 131.9 ($C_{3'}$), 116.2 (C_5), 81.0 (d, *J* = 5.4 Hz, $C_{1'}$), 58.9 ($C_{4'}$), 54.0 (OCH_3), 52.7 (d, *J* = 9.2 Hz, OCH_3), 52.6 (d, *J* = 9.2 Hz, OCH_3), 47.3 (d, *J* = 10.2 Hz, $C_{5'}$), 36.4 (d, *J* = 135.6 Hz, $C_{6'}$).

UV (EtOH) λ_{max} = 284 nm (ε_{max} = 8300), λ_{max} = 245 nm (ε_{max} = 8000). MS ESI-QTof>0 *m/z* 370.13 $[M+H]^+$. HRMS calculated for $C_{14}H_{21}N_5O_5P$: 370.1280; found: 370.1277.

(+/−)-Sodium hydrogen ((4-(6-amino-9*H*-purin-9-yl)-1-hydroxycyclopent-2-en-1-yl)methyl)phosphonate ((+/−)-26)

To a solution of (+/−)-**24** (60 mg, 0.176 mmol) in DMF (2 mL) at 0 °C was added dropwise trimethylsilyl bromide (230 µL, 1.76 mmol) and the reaction mixture was stirred for 1 h at 0 °C and 16 h at rt. After the addition of TEAB until pH 7, evaporation of solvents and purification by reversed phase chromatography (H_2O/MeOH 1:0 to 1:1) followed by Dowex chromatography (Na^+) compound (+/−)-**26** (7 mg, 12%) was obtained as a white solid. Rf (*i*-PrOH/NH_4OH/H_2O: 7/2/1): 0.3 ^{1}H NMR (D_2O, 400 MHz): δ = 8.12 (s, 1H, H_2), 8.10 (s, 1H, H_8), 6.39 (dd, *J* = 2.1, 5.6 Hz, 1H, $H_{2'}$), 6.14 (dd, *J* = 2.0, 5.6 Hz, 1H, $H_{3'}$), 5.75-5.72 (m, 1H, $H_{4'}$), 2.71 (dd, *J* = 7.7, 14.5 Hz, 1H, $H_{5'a}$), 2.38 (dd, *J* = 5.6, 14.5 Hz, 1H, $H_{5'b}$), 2.17 (dd, *J* = 18.1 Hz, 2H, $H_{6'}$). ^{31}P NMR (D_2O, 162 MHz): δ = 18.89. ^{13}C NMR (D_2O, 100 MHz): δ = 155.2 (C_4), 152.0 (C_2), 148.4 (C_6), 141.6 (d, *J* = 7.4 Hz, $C_{2'}$), 140.4 (C_8), 130.8 ($C_{3'}$), 118.5 (C_5), 82.0 (d, *J* = 2.9 Hz, $C_{1'}$), 59.3 ($C_{4'}$), 45.7 (d, *J* =

6.6 Hz, $C_{5'}$), 38.8 (d, *J* = 127.9 Hz, $C_{6'}$). MS ESI-QTof>0 *m/z* 334.07 $[M+H]^+$. HRMS calculated for $C_{11}H_{14}N_5O_4NaP$: 334.0681; found: 334.0684.

(+/−)-Sodium hydrogen ((4-(2-amino-6-oxo-1*H*-purin-9(6*H*)-yl)-1-hydroxycyclopent-2-en-1-yl)methyl)phosphonate ((+/−)-27)

To a solution of (+/−)-**25** (250 mg, 0.677 mmol) in DMF (5 mL) at 0 °C was added dropwise trimethylsilyl bromide (875 µL, 6.77 mmol) and the reaction mixture was stirred for 1 h at 0 °C and 15 h at rt. After the addition of TEAB until pH 7, evaporation of solvents and purification by reversed phase chromatography (H_2O/MeOH 1:0 to 1:1) followed by Dowex chromatography (Na^+) compound (+/−)-**27** (37 mg, 17%) was obtained as a white solid. Rf (*i*-PrOH/NH_4OH/H_2O: 7/2/1): 0.1. ^{1}H NMR (D_2O, 300 MHz): δ = 7.80 (s, 1H, H_8), 6.35 (dd, *J* = 2.1, 5.6 Hz, 1H, $H_{2'}$), 6.09 (dd, *J* = 2.0, 5.6 Hz, 1H, $H_{3'}$), 5.61-5.57 (m, 1H, $H_{4'}$), 2.68 (dd, *J* = 7.7, 14.5 Hz, 1H, $H_{5'a}$), 2.35 (dd, *J* = 5.7, 14.5 Hz, 1H, $H_{5'b}$), 2.17 (d, *J* = 17.0 Hz, 2H, $H_{6'}$). ^{31}P NMR (D_2O, 127 MHz): δ = 19.01. ^{13}C NMR (D_2O, 75 MHz): δ = 158.8 (C=O),153.5 (C_2), 151.1 (C_4), 141.2 (d, *J* = 7.3 Hz, $C_{2'}$), 138.0 (C_8), 131.2 ($C_{3'}$), 116.1 (C_5), 82.1 (d, *J* = 2.9 Hz, $C_{1'}$), 58.9 ($C_{4'}$), 45.8 (d, *J* = 6.9 Hz, $C_{5'}$), 38.8 (d, *J* = 127.9 Hz, $C_{6'}$). UV (H_2O) λ_{max} = 252 nm (ε_{max} = 9300). MS ESI-QTof>0 *m/z* 328.08 $[M+H]^+$. HRMS calculated for $C_{11}H_{15}N_5O_5P$: 328.0811; found: 328.0812.
