# Supplementary material for: Revaluation of biomass-derived furfuryl alcohol derivatives for the synthesis of carbocyclic nucleoside phosphonate analogues
Source: Beilstein J Org Chem. 2017 Feb 9;13:251–6. doi: 10.3762/bjoc.13.28 (PMC5331271; doi:10.3762/bjoc.13.28)
Supplement: File 2 — Copies of NMR spectra for the synthesized compounds. [file Beilstein_J_Org_Chem-13-251-s002.pdf]

# Supporting Information File 2

# for

# Revaluation of biomass-derived furfuryl alcohol derivatives for the synthesis of carbocyclic nucleoside phosphonate analogues

Bemba Sidi Mohamed, Christian Périgaud and Christophe Mathé*

Address: Institut des Biomolécules Max Mousseron (IBMM), UMR 5247, Université de Montpellier, CNRS, ENSCM, cc 1705, Site Triolet, Place Eugène Bataillon 34095 Montpellier cedex 5, France

Email: Christophe Mathé - christophe.mathe@umontpellier.fr

*Corresponding author

## Copies of NMR spectra for the synthesized compounds

$^{1}H$ NMR, $^{31}P$ NMR and $^{13}C$ NMR for compound (+/−)-**2** S3

$^{1}H$ NMR, $^{31}P$ NMR and $^{13}C$ NMR for compound (+/−)-**3** S4

$^{1}H$ NMR, $^{31}P$ NMR and $^{13}C$ NMR for compound (+/−)-**4** S6

$^{1}H$ NMR, $^{31}P$ NMR and $^{13}C$ NMR for compound (+/−)-**5** S7

$^{1}H$ NMR, $^{31}P$ NMR and $^{13}C$ NMR for compound (+/−)-**6** S9

$^{1}H$ NMR, $^{31}P$ NMR and $^{13}C$ NMR for compound (+/−)-**7** S10

$^{1}H$ NMR, $^{31}P$ NMR and $^{13}C$ NMR for compound (+/−)-**8** S12

$^{1}H$ NMR, $^{31}P$ NMR and $^{13}C$ NMR for compound (+/−)-**9** S13

$^{1}H$ NMR, $^{31}P$ NMR and $^{13}C$ NMR for compound (+/−)-**10** S15

$^{1}H$ NMR, $^{31}P$ NMR and $^{13}C$ NMR for compound (+/−)-**11** S18

$^{1}H$ NMR, $^{31}P$ NMR and $^{13}C$ NMR for compound (+/−)-**12** S19

$^{1}H$ NMR, $^{31}P$ NMR and $^{13}C$ NMR for compound (+/−)-**13** S21

$^{1}H$ NMR, $^{31}P$ NMR and $^{13}C$ NMR for compound (+/−)-**14** S22

$^{1}H$ NMR, $^{31}P$ NMR and $^{13}C$ NMR for compound (+/−)-**15** S24

$^{1}H$ NMR, $^{31}P$ NMR and $^{13}C$ NMR for compound (+/−)-**16** S25

NOESY spectrum of (+/−)-**16** S27

$^{1}H$ NMR and $^{13}C$ NMR for compound (+/−)-**17** S28

$^{1}H$ NMR, $^{31}P$ NMR and $^{13}C$ NMR for compound (+/−)-**18** S29

$^{1}H$ NMR, $^{31}P$ NMR and $^{13}C$ NMR for compound (+/−)-**19** S30

$^{1}H$ NMR, $^{31}P$ NMR and $^{13}C$ NMR for compound (+/−)-**20** S32

$^{1}H$ NMR, $^{31}P$ NMR and $^{13}C$ NMR for compound (+/−)-**21** S33

$^{1}H$ NMR, $^{31}P$ NMR and $^{13}C$ NMR for compound (+/−)-**22** S35

$^{1}H$ NMR, $^{31}P$ NMR and $^{13}C$ NMR for compound (+/−)-**23** S36

$^{1}H$ NMR, $^{31}P$ NMR and $^{13}C$ NMR for compound (+/−)-**24** S38

$^{1}H$ NMR, $^{31}P$ NMR and $^{13}C$ NMR for compound (+/−)-**25** S39

$^{1}H$ NMR, $^{31}P$ NMR and $^{13}C$ NMR for compound (+/−)-**26** S41

$^{1}H$ NMR, $^{31}P$ NMR and $^{13}C$ NMR for compound (+/−)-**27** S42

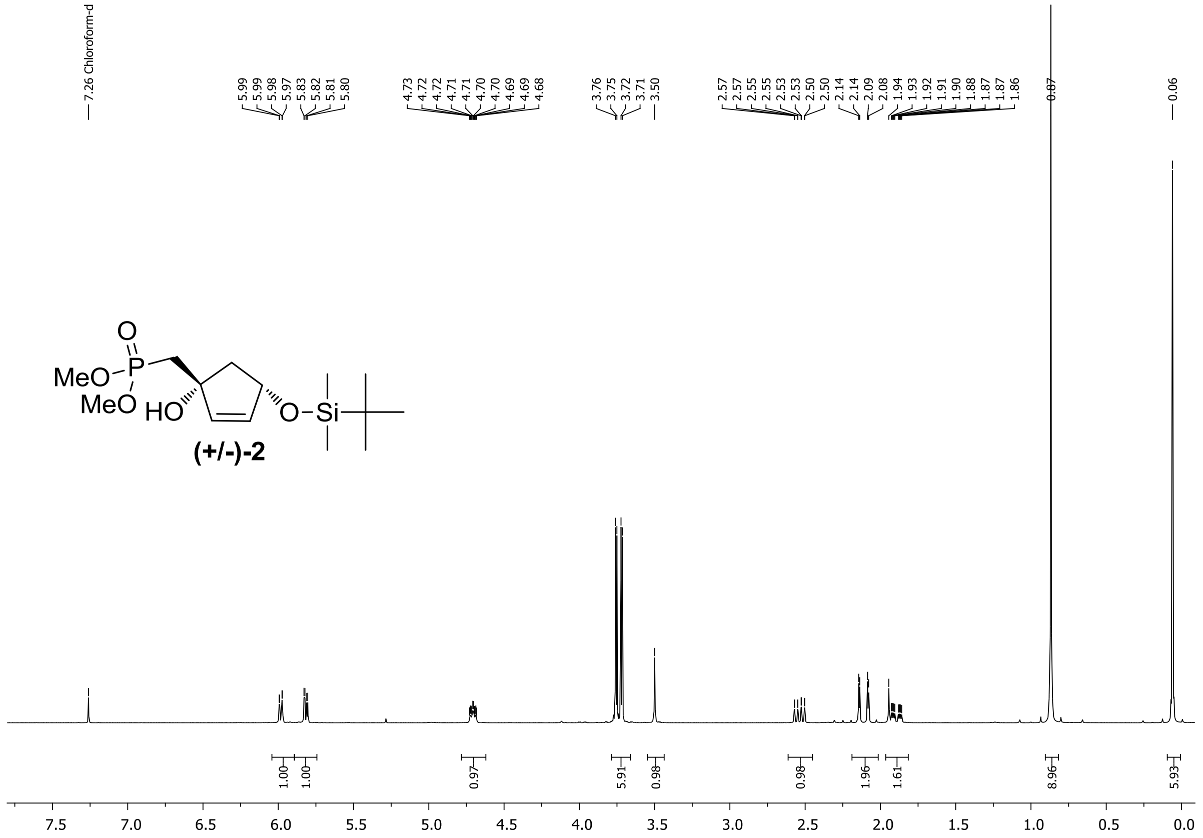
7.26 Chloroform-d
5.99 5.99 5.98 5.97 5.83 5.82 5.81 5.80
4.73 4.72 4.72 4.71 4.71 4.70 4.70 4.69 4.69 4.68
3.76 3.75 3.72 3.71 3.50
2.57 2.57 2.55 2.55 2.53 2.53 2.50 2.50 2.14 2.14 2.09 2.08 1.94 1.93 1.93 1.92 1.91 1.90 1.88 1.87 1.87 1.86
0.87
0.06
MeO
MeO
O
P
HO
O
Si
(+/-)-2
1.00 1.00 0.97 5.91 0.98 0.98 1.96 1.61 8.96 5.93
7.5 7.0 6.5 6.0 5.5 5.0 4.5 4.0 3.5 3.0 2.5 2.0 1.5 1.0 0.5 0.0

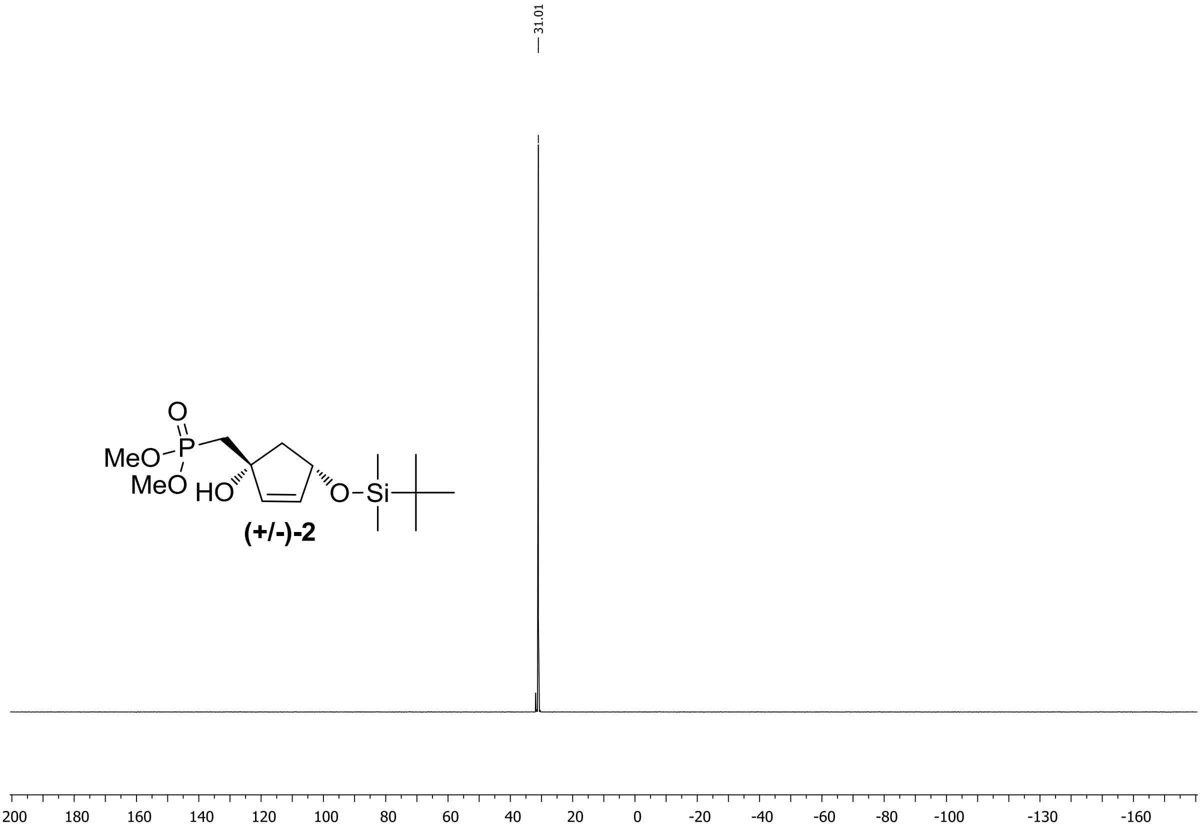
31.01
MeO
MeO
O
P
HO
O
Si
(+/-)-2
200 180 160 140 120 100 80 60 40 20 0 -20 -40 -60 -80 -100 -130 -160

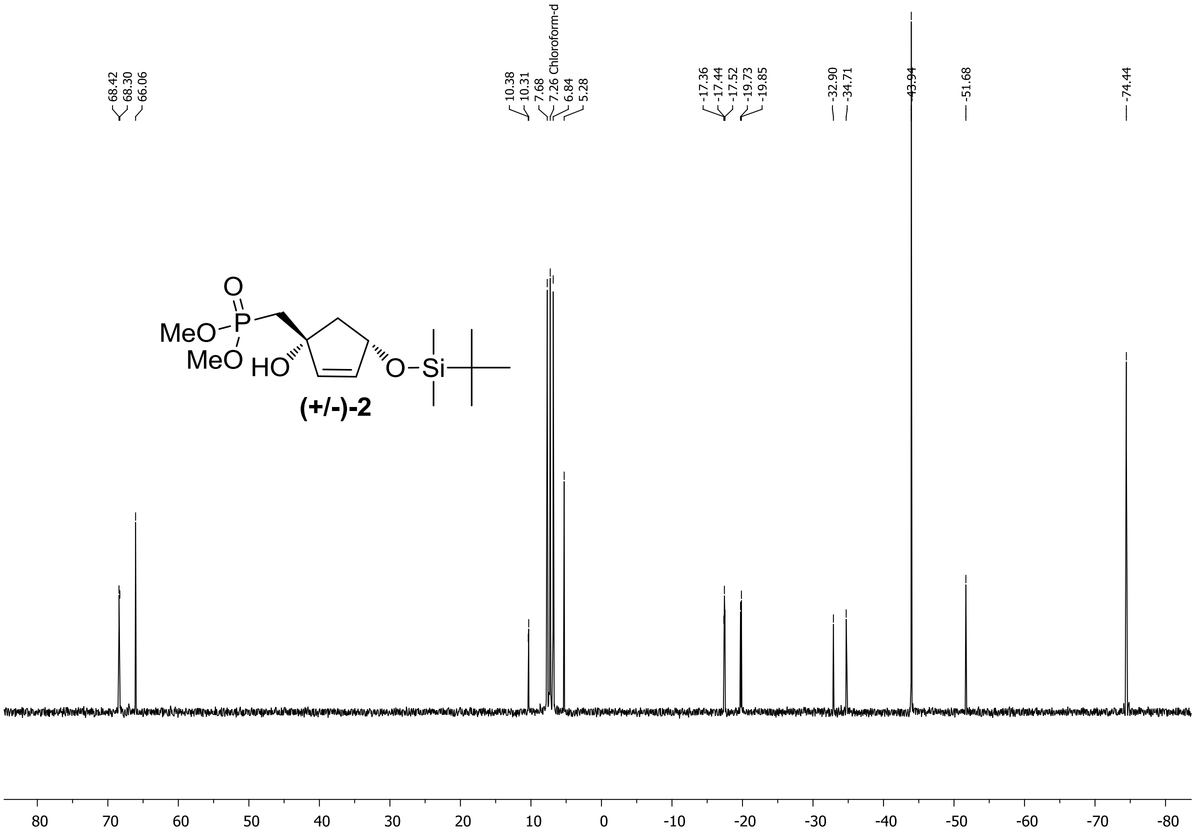
68.42
68.30
66.06
10.38
10.31
7.68
7.26 Chloroform-d
6.84
5.28
-17.36
-17.44
-17.52
-19.73
-19.85
-32.90
-34.71
-43.94
-51.68
-74.44
MeO
MeO
HO
O
P
O-Si
(+/-)-2
80 70 60 50 40 30 20 10 0 -10 -20 -30 -40 -50 -60 -70 -80

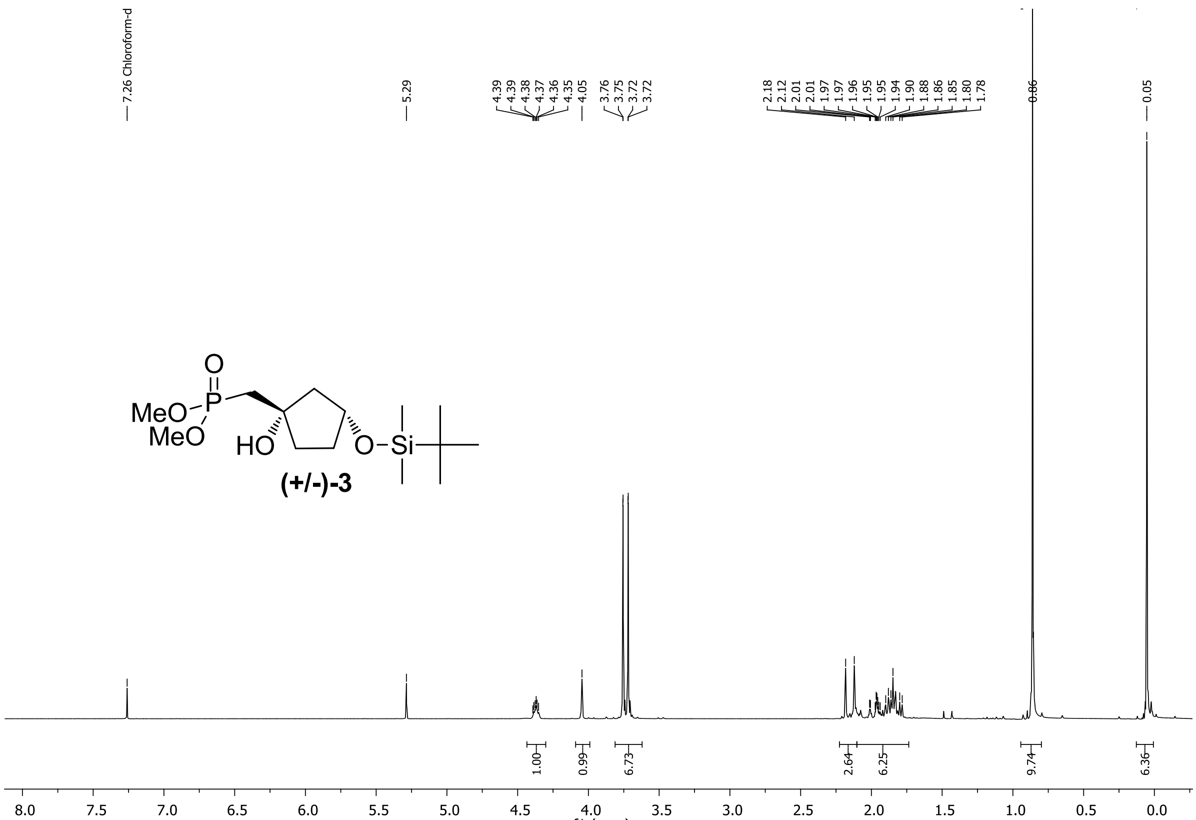
7.26 Chloroform-d
5.29
4.39
4.39
4.38
4.37
4.36
4.35
4.05
3.76
3.75
3.72
3.72
2.18
2.12
2.01
2.01
1.97
1.96
1.95
1.95
1.94
1.90
1.88
1.86
1.85
1.80
1.78
0.86
0.05
MeO
MeO
HO
O
P
O-Si
(+/-)-3
1.00
0.99
6.73
2.64
6.25
9.74
6.36
8.0 7.5 7.0 6.5 6.0 5.5 5.0 4.5 4.0 3.5 3.0 2.5 2.0 1.5 1.0 0.5 0.0
f1 (ppm)

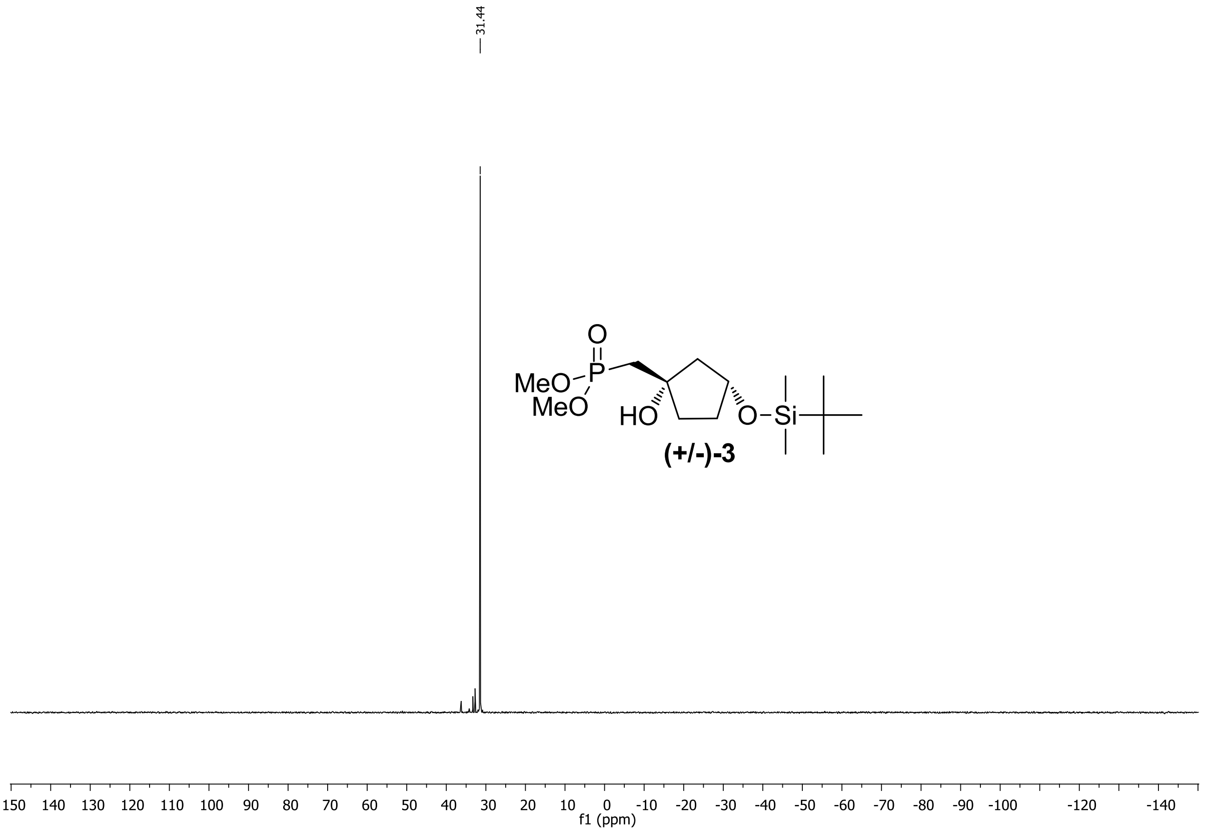
31.44
MeO
MeO
P
O
HO
O
Si
(+/-)-3
150 140 130 120 110 100 90 80 70 60 50 40 30 20 10 0 -10 -20 -30 -40 -50 -60 -70 -80 -90 -100 -120 -140
f1 (ppm)

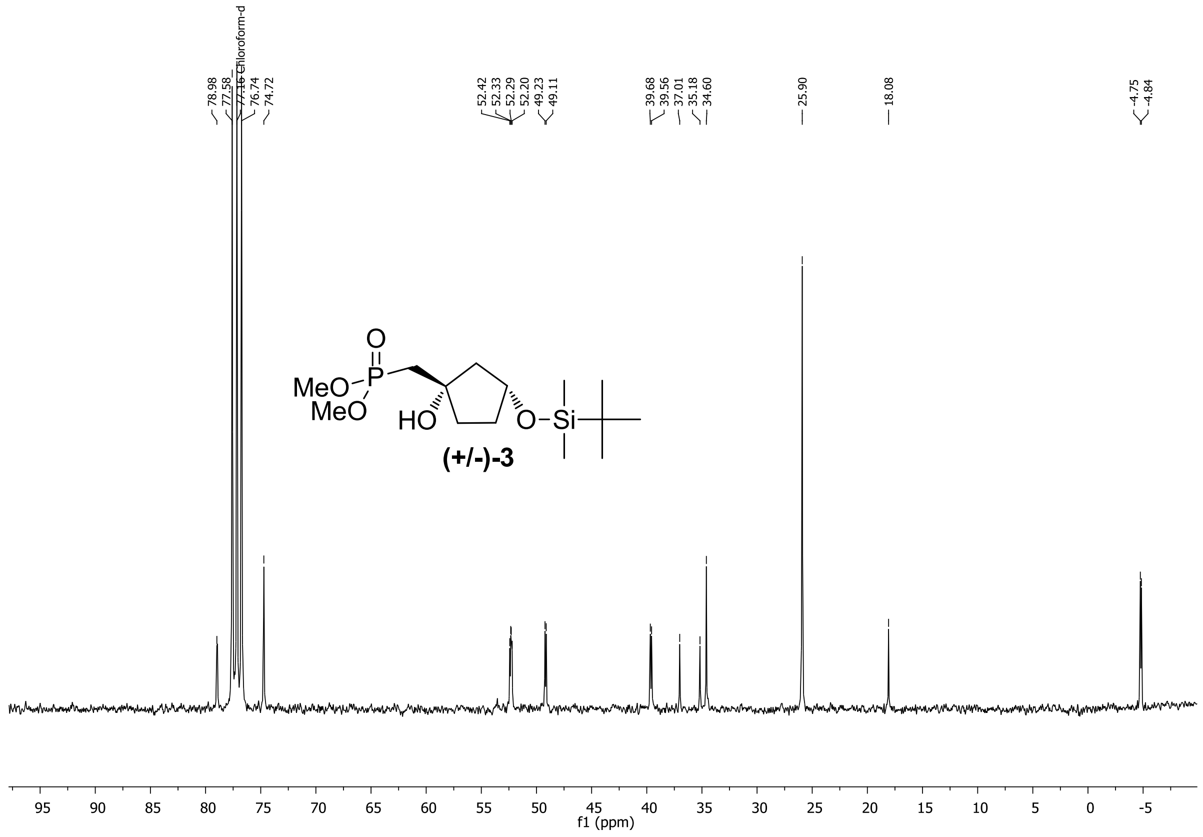
78.98
77.58
77.16 Chloroform-d
76.74
74.72
52.42
52.33
52.29
52.20
49.23
49.11
39.68
39.56
37.01
35.18
34.60
25.90
18.08
-4.75
-4.84
MeO
MeO
P
O
HO
O
Si
(+/-)-3
95 90 85 80 75 70 65 60 55 50 45 40 35 30 25 20 15 10 5 0 -5
f1 (ppm)

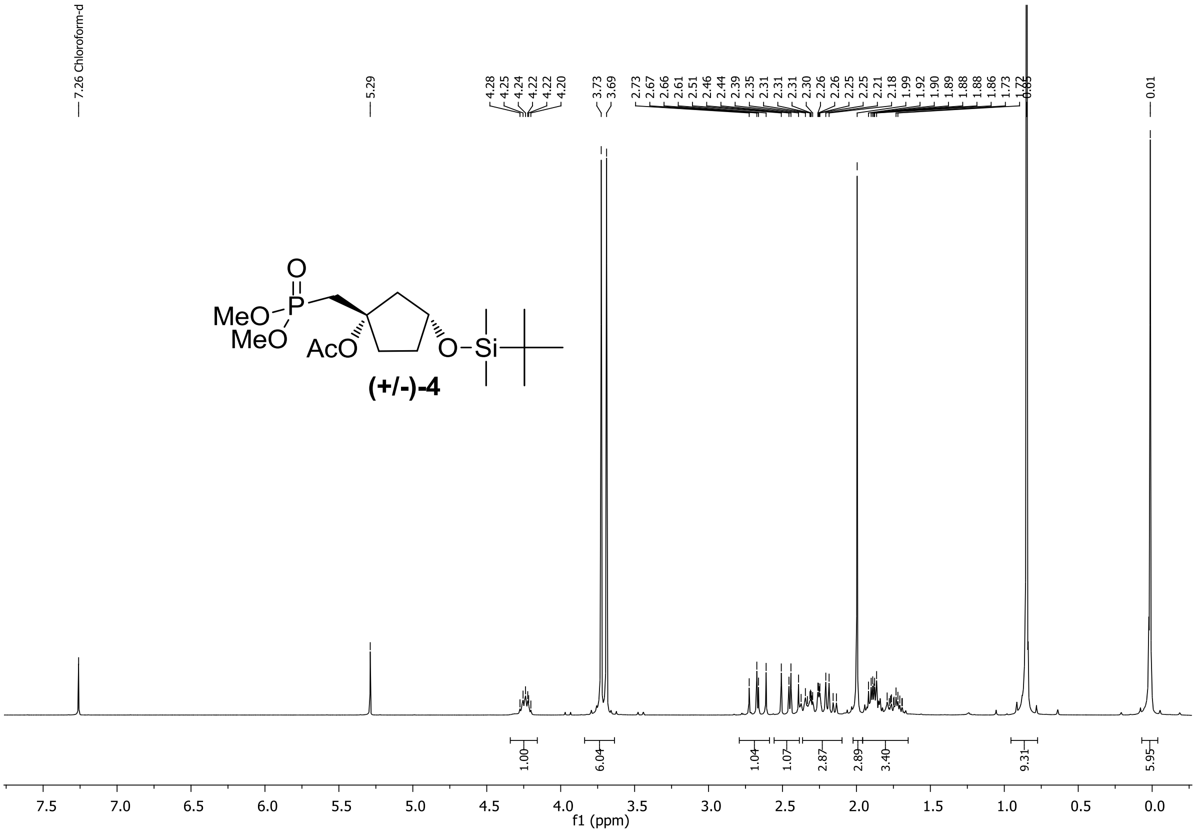
7.26 Chloroform-d
5.29
4.28 4.25 4.24 4.22 4.22 4.20
3.73 3.69
2.73 2.67 2.66 2.61 2.51 2.46 2.44 2.39 2.35 2.31 2.31 2.31 2.30 2.26 2.26 2.25 2.25 2.21 2.18 1.99 1.92 1.90 1.89 1.88 1.88 1.86 1.73 1.72 0.85
0.01
MeO
MeO
P
O
AcO
O
Si
(+/-)-4
1.00
6.04
1.04
1.07
2.87
2.89
3.40
9.31
5.95
7.5 7.0 6.5 6.0 5.5 5.0 4.5 4.0 3.5 3.0 2.5 2.0 1.5 1.0 0.5 0.0
f1 (ppm)

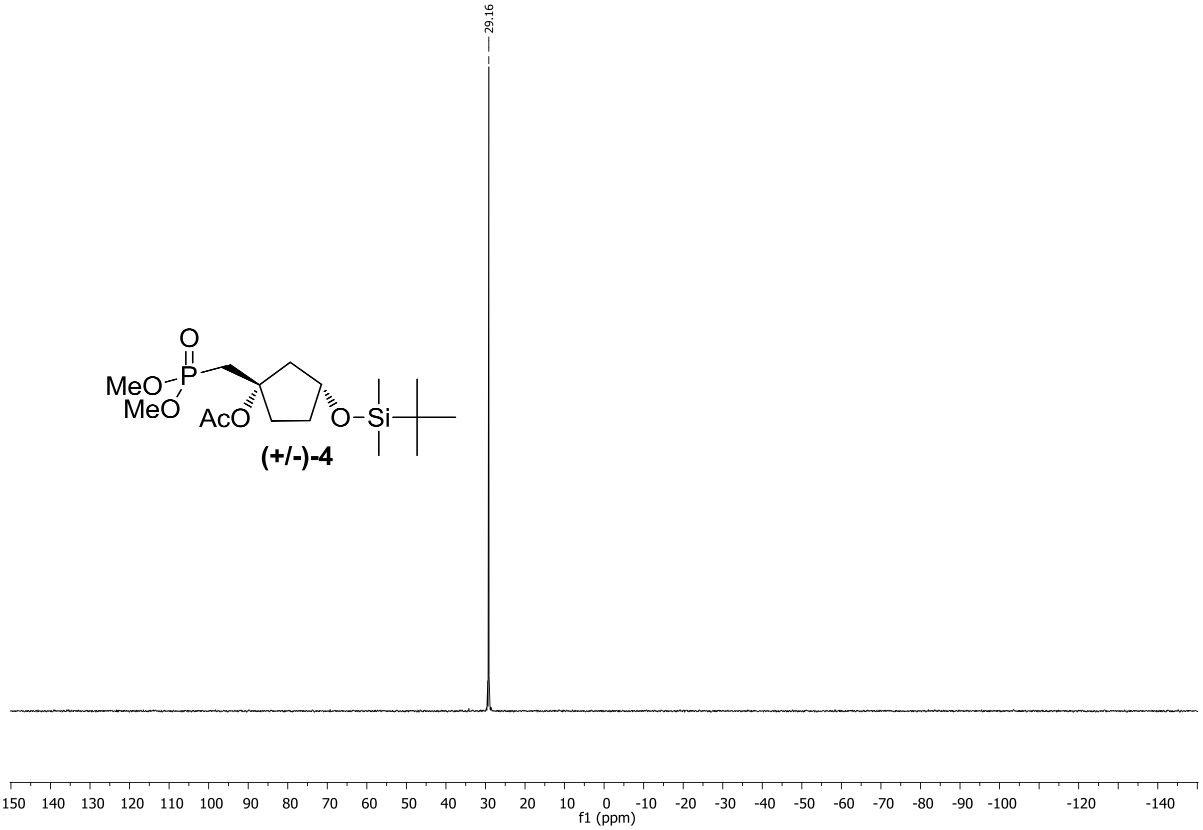
29.16
MeO
MeO
P
O
AcO
O
Si
(+/-)-4
150 140 130 120 110 100 90 80 70 60 50 40 30 20 10 0 -10 -20 -30 -40 -50 -60 -70 -80 -90 -100 -120 -140
f1 (ppm)

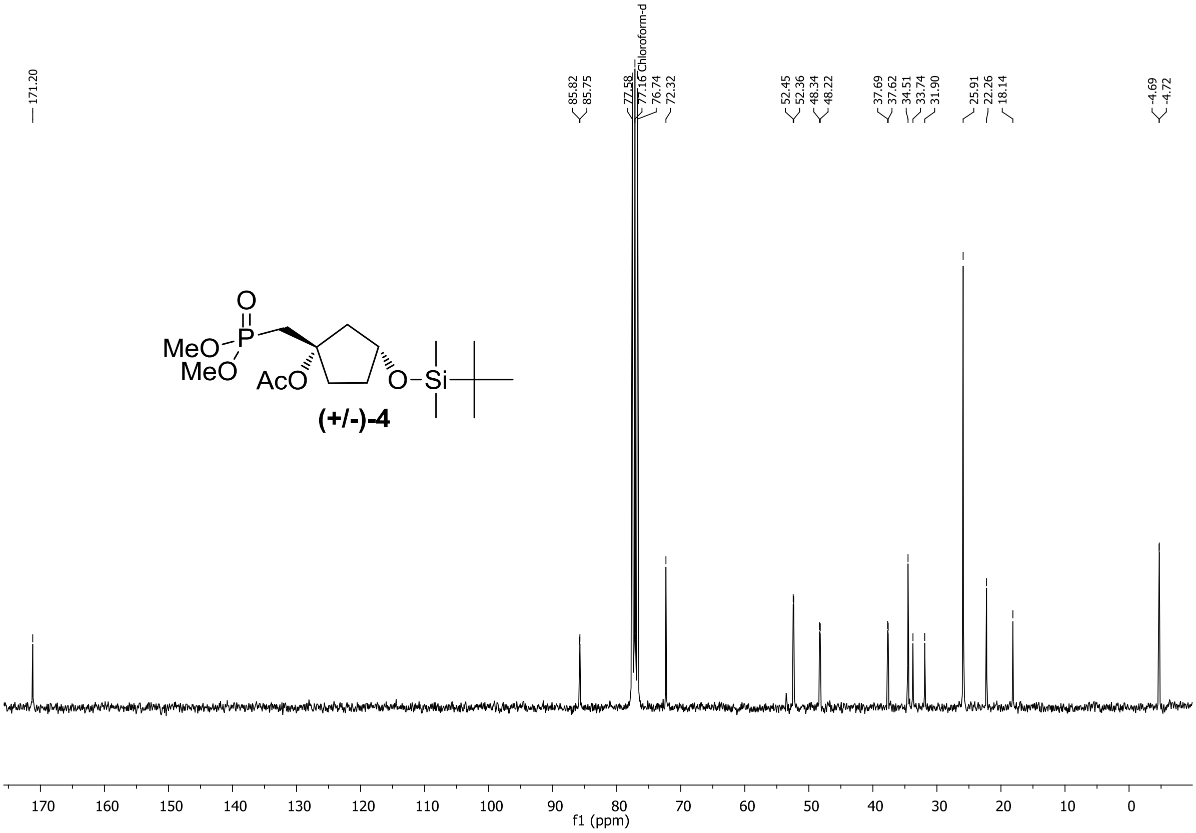
171.20
85.82
85.75
77.58
77.16 Chloroform-d
76.74
72.32
52.45
52.36
48.34
48.22
37.69
37.62
34.51
33.74
31.90
25.91
22.26
18.14
-4.69
-4.72
MeO
MeO
AcO
O
P
O-Si
(+/-)-4
170 160 150 140 130 120 110 100 90 80 70 60 50 40 30 20 10 0
f1 (ppm)

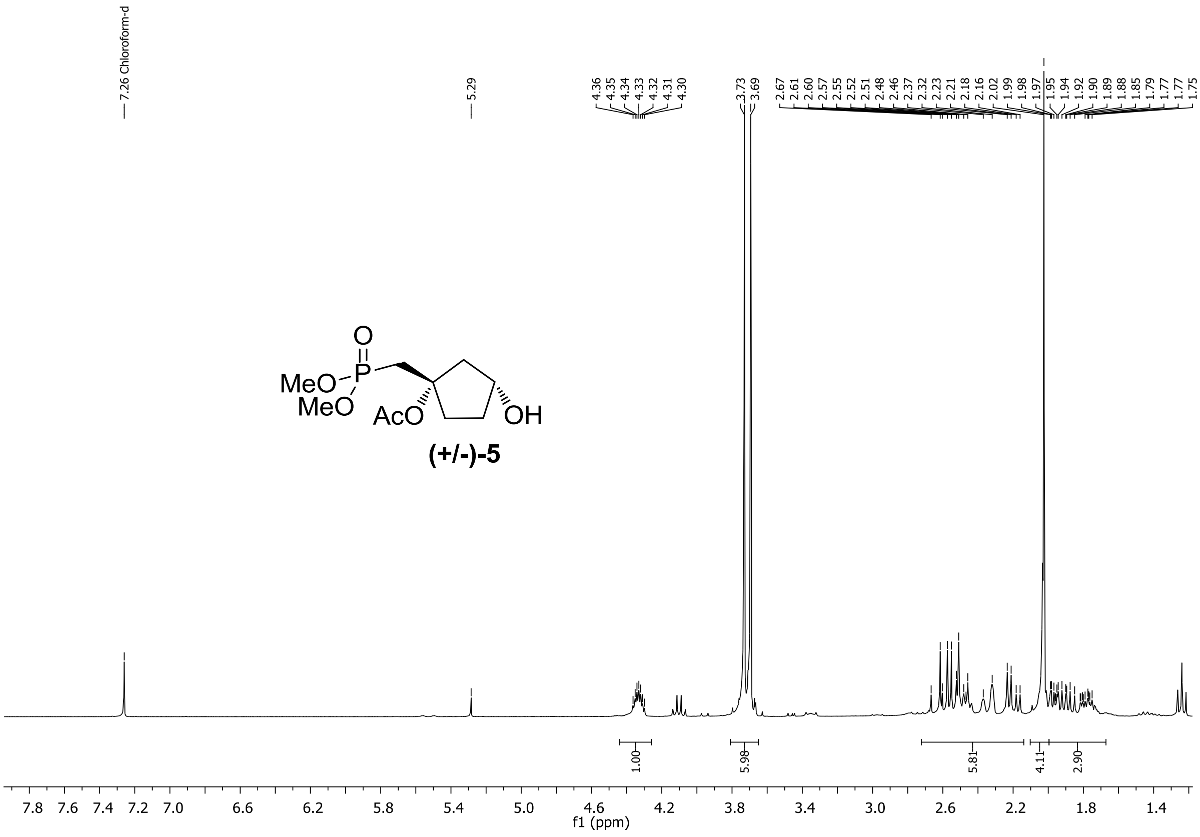
7.26 Chloroform-d
5.29
4.36
4.35
4.34
4.33
4.32
4.31
4.30
3.73
3.69
2.67
2.61
2.60
2.57
2.55
2.52
2.51
2.48
2.46
2.37
2.32
2.23
2.21
2.18
2.16
2.02
1.99
1.98
1.97
1.95
1.94
1.92
1.90
1.89
1.88
1.85
1.79
1.77
1.75
MeO
MeO
AcO
O
P
OH
(+/-)-5
1.00
5.98
5.81
4.11
2.90
7.8 7.6 7.4 7.2 7.0 6.6 6.2 5.8 5.4 5.0 4.6 4.2 3.8 3.4 3.0 2.6 2.2 1.8 1.4
f1 (ppm)

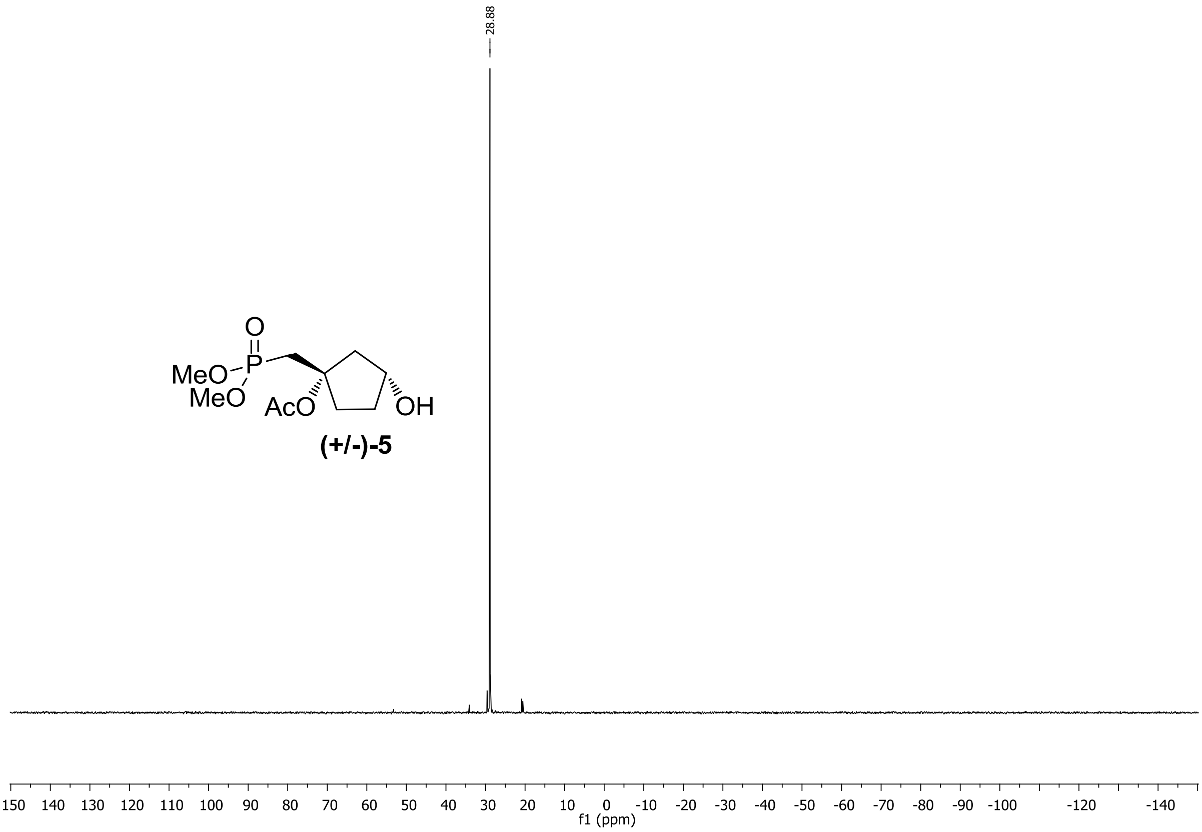

28.88
MeO
MeO
AcO
OH
(+/-)-5
150 140 130 120 110 100 90 80 70 60 50 40 30 20 10 0 -10 -20 -30 -40 -50 -60 -70 -80 -90 -100 -120 -140
f1 (ppm)


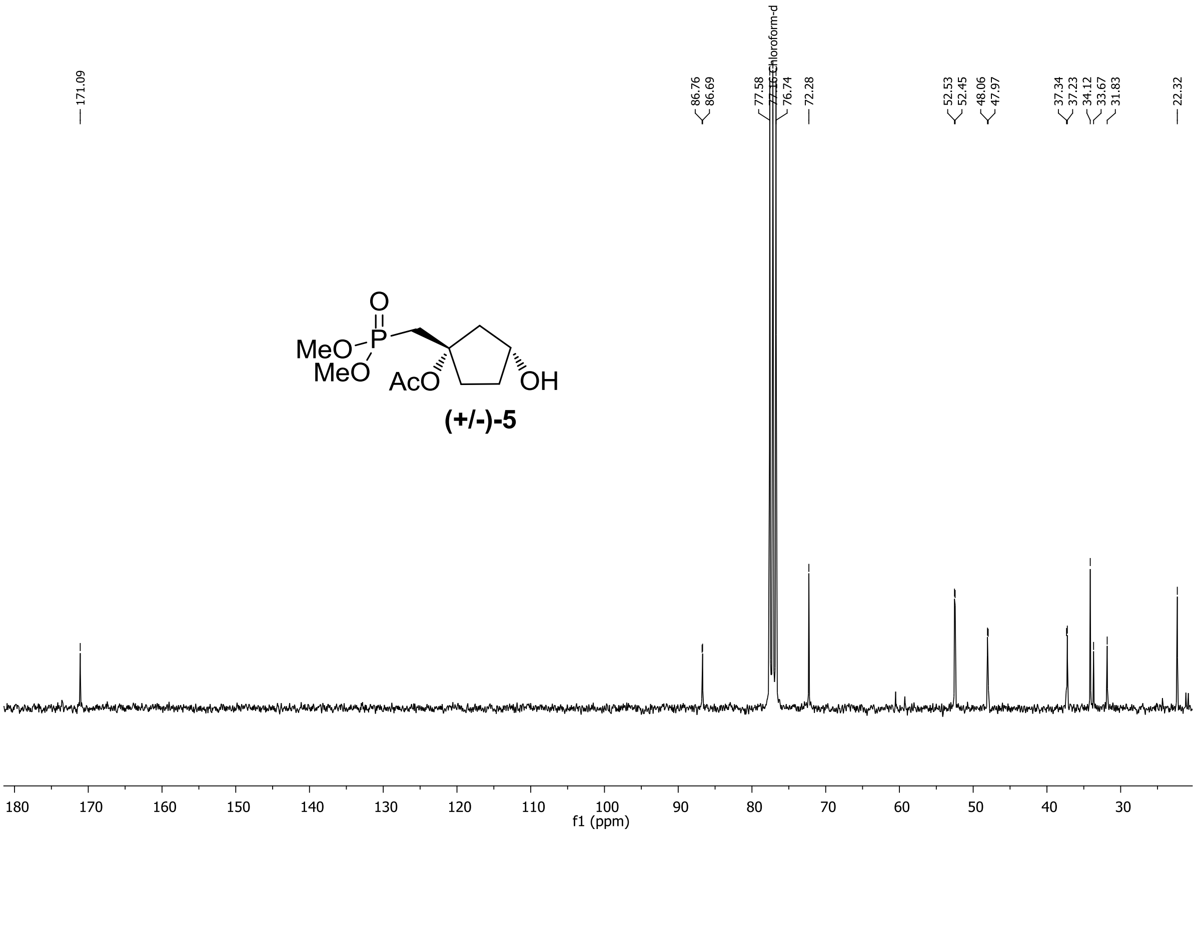

171.09
86.76
86.69
77.58
77.16 Chloroform-d
76.74
72.28
52.53
52.45
48.06
47.97
37.34
37.23
34.12
33.67
31.83
22.32
MeO
MeO
AcO
OH
(+/-)-5
180 170 160 150 140 130 120 110 100 90 80 70 60 50 40 30
f1 (ppm)

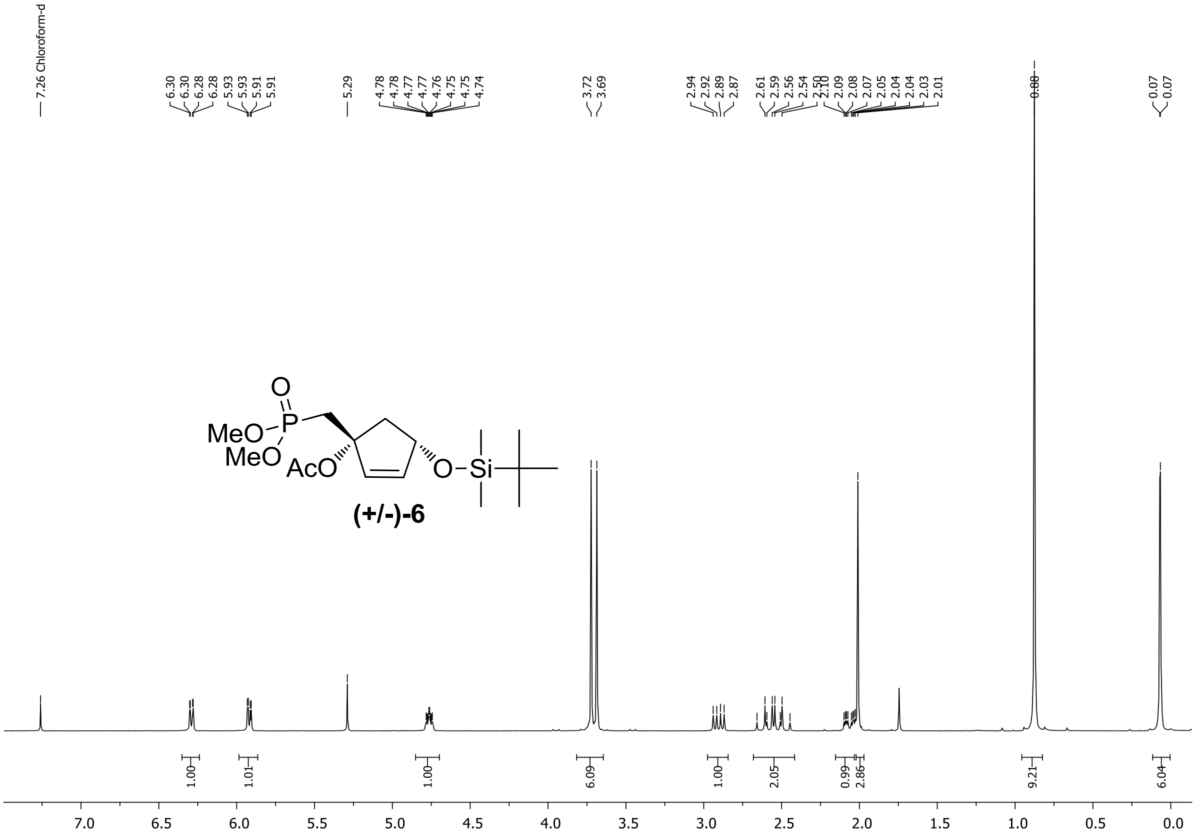
7.26 Chloroform-d
6.30 6.30 6.28 6.28 5.93 5.93 5.91 5.91
5.29
4.78 4.78 4.77 4.77 4.76 4.75 4.75 4.74
3.72 3.69
2.94 2.92 2.89 2.87
2.61 2.59 2.56 2.54 2.10 2.09 2.08 2.07 2.05 2.04 2.04 2.03 2.01
0.88
0.07 0.07
MeO MeO P O AcO O Si
(+/-)-6
1.00 1.01 1.00 6.09 1.00 2.05 0.99 2.86 9.21 6.04
7.0 6.5 6.0 5.5 5.0 4.5 4.0 3.5 3.0 2.5 2.0 1.5 1.0 0.5 0.0

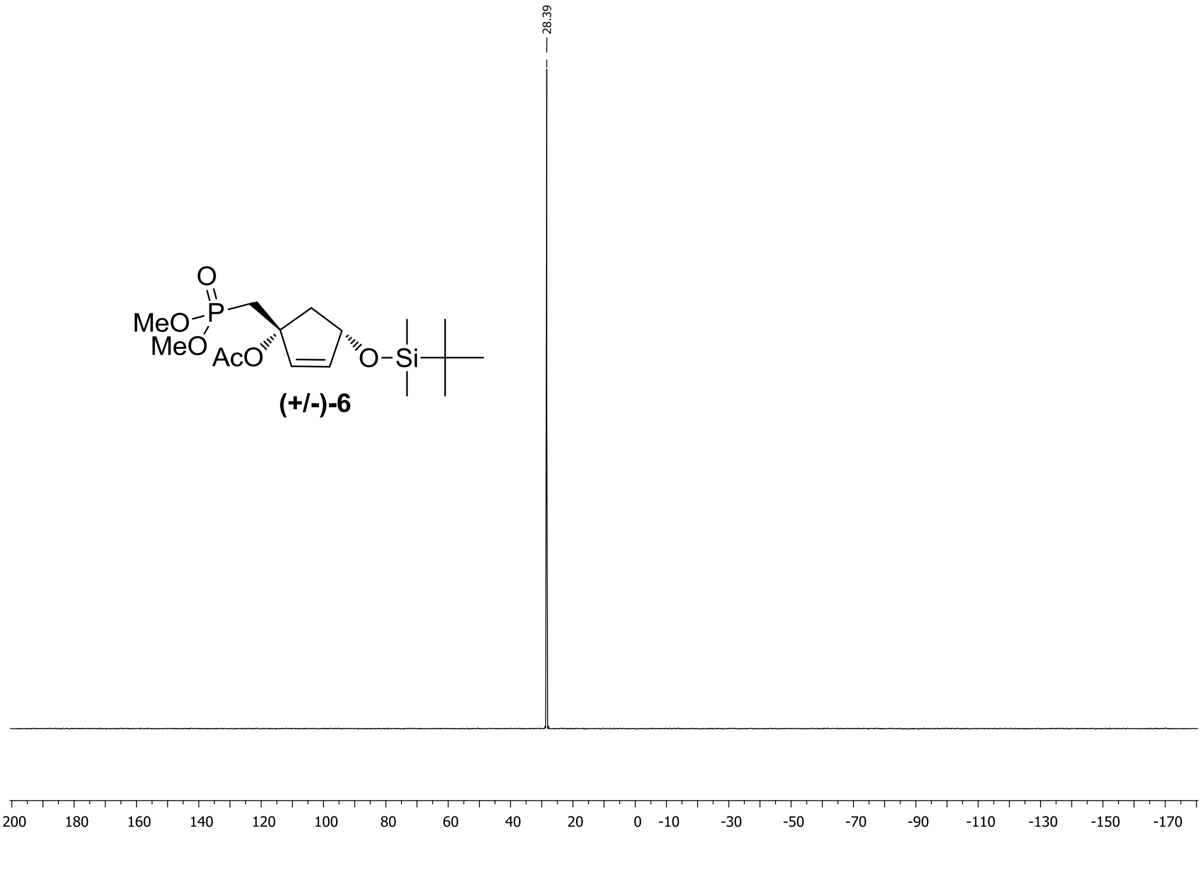
28.39
MeO MeO P O AcO O Si
(+/-)-6
200 180 160 140 120 100 80 60 40 20 0 -10 -30 -50 -70 -90 -110 -130 -150 -170

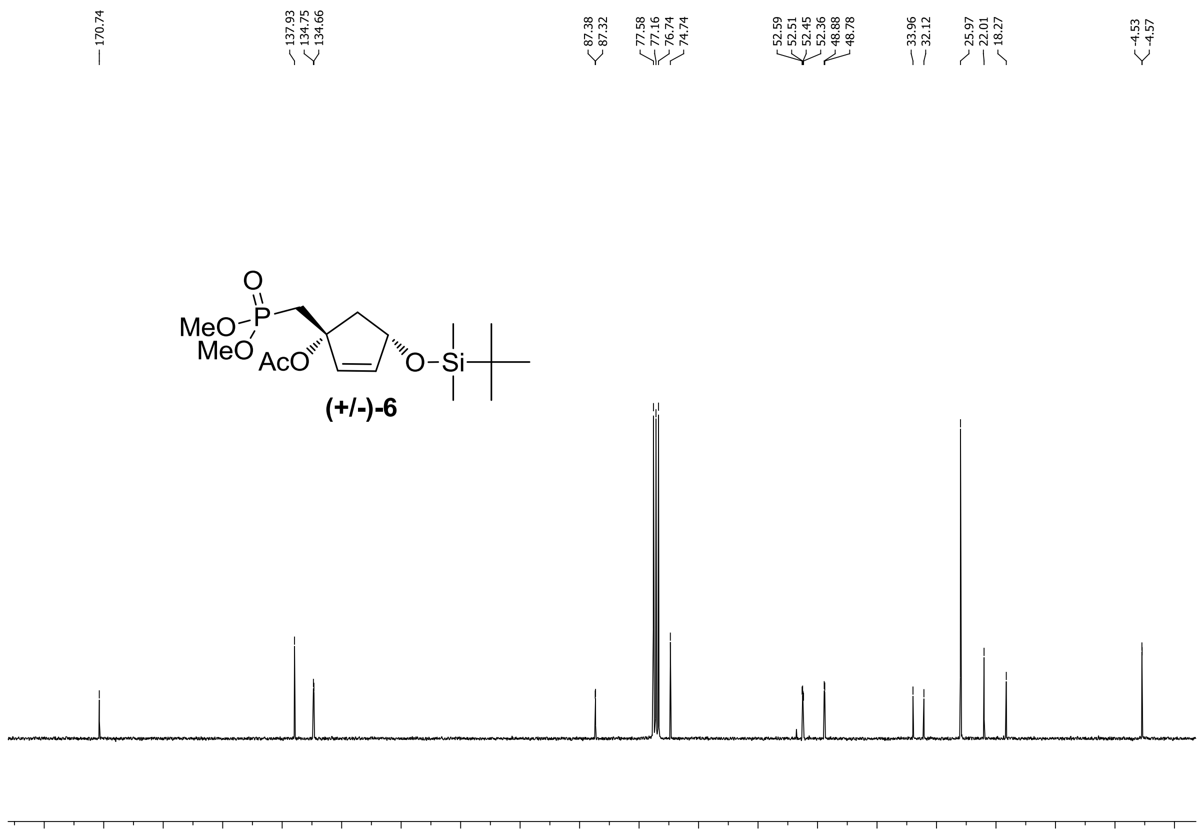
170.74
137.93
134.75
134.66
87.38
87.32
77.58
77.16
76.74
74.74
52.59
52.51
52.45
52.36
48.88
48.78
33.96
32.12
25.97
22.01
18.27
-4.53
-4.57
MeO
MeO
AcO
O
P
O-Si
180 170 160 150 140 130 120 110 100 90 80 70 60 50 40 30 20 10 0 -10

(+/-)-6

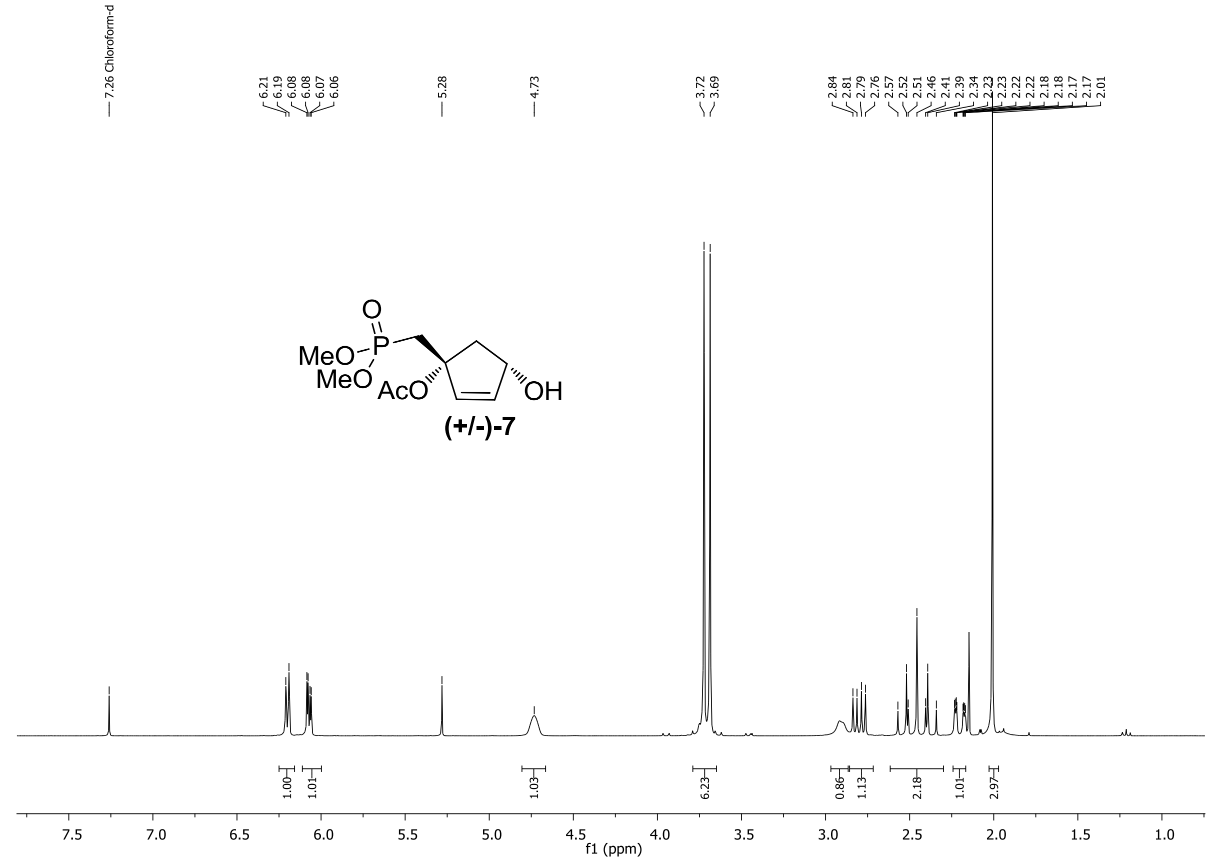
7.26 Chloroform-d
6.21
6.19
6.08
6.08
6.07
6.06
5.28
4.73
3.72
3.69
2.84
2.81
2.79
2.76
2.57
2.52
2.51
2.46
2.41
2.39
2.34
2.23
2.23
2.22
2.22
2.18
2.18
2.17
2.01
MeO
MeO
AcO
O
P
OH
1.00
1.01
1.03
6.23
0.86
1.13
2.18
1.01
2.97
7.5 7.0 6.5 6.0 5.5 5.0 4.5 4.0 3.5 3.0 2.5 2.0 1.5 1.0
f1 (ppm)

(+/-)-7

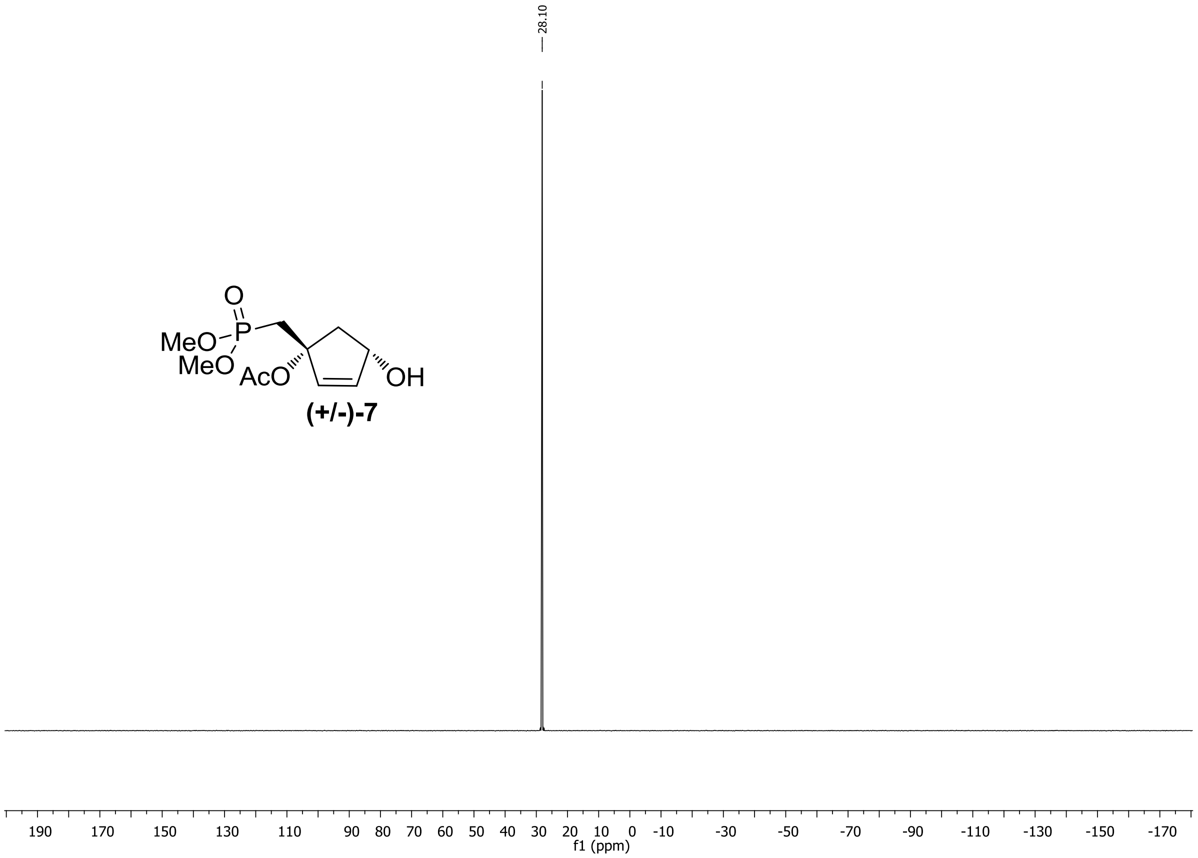

28.10
MeO
MeO
AcO
OH
O
P
(+/-)-7
190 170 150 130 110 90 80 70 60 50 40 30 20 10 0 -10 -30 -50 -70 -90 -110 -130 -150 -170
f1 (ppm)


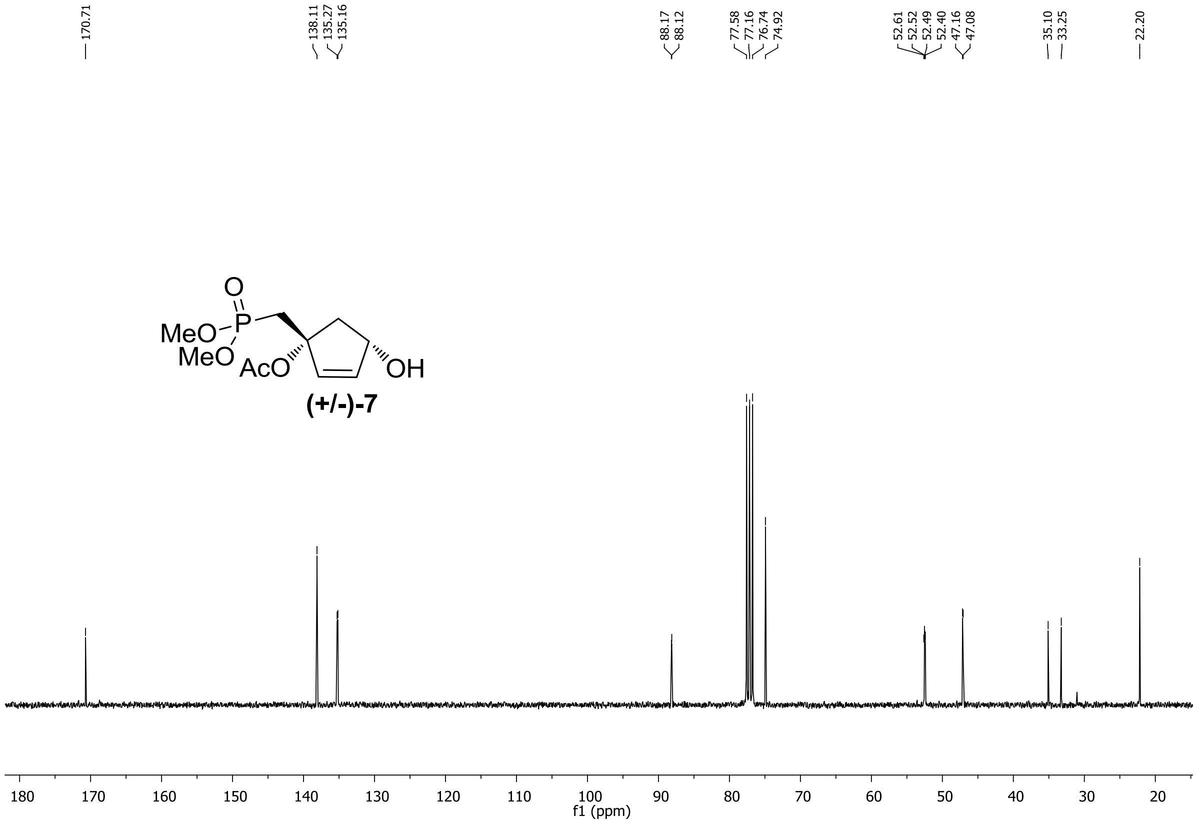

170.71
138.11
135.27
135.16
88.17
88.12
77.58
77.16
76.74
74.92
52.61
52.52
52.49
52.40
47.16
47.08
35.10
33.25
22.20
MeO
MeO
AcO
OH
O
P
(+/-)-7
180 170 160 150 140 130 120 110 100 90 80 70 60 50 40 30 20
f1 (ppm)

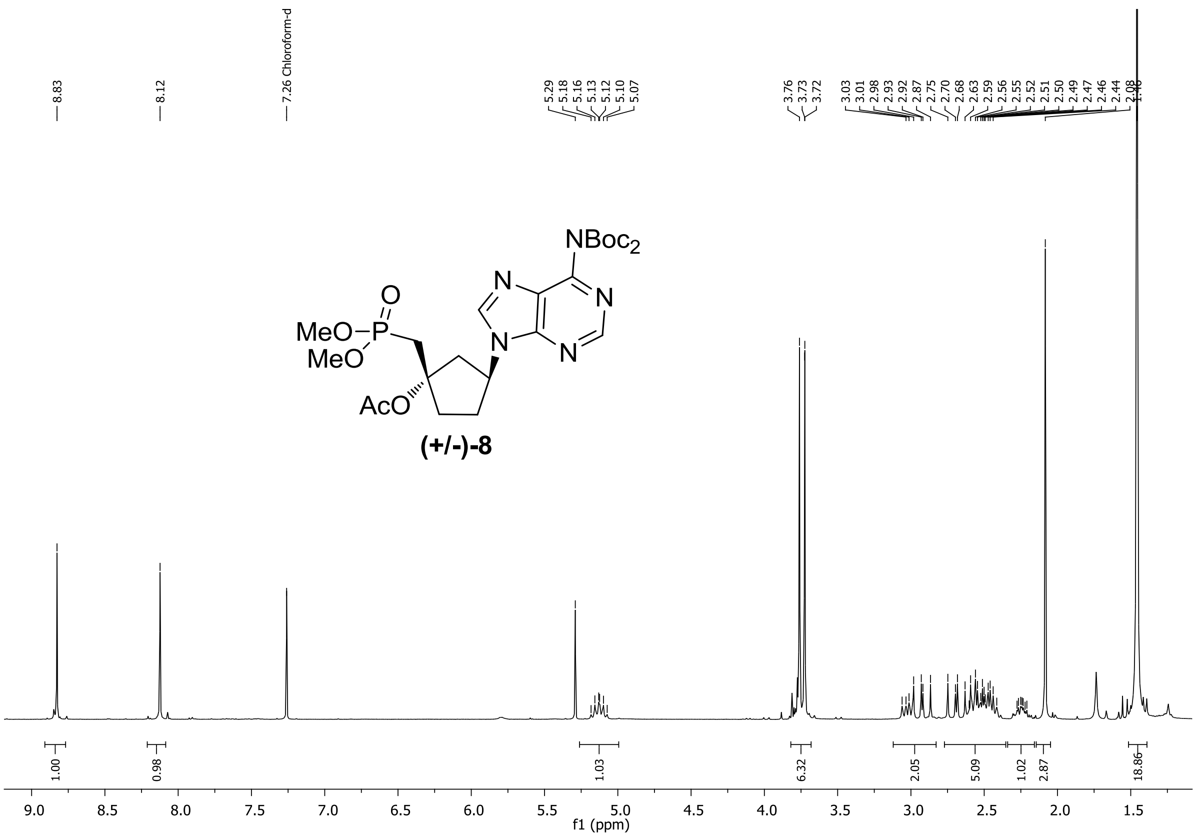
8.83
8.12
7.26 Chloroform-d
5.29
5.18
5.16
5.13
5.12
5.10
5.07
3.76
3.73
3.72
3.03
3.01
2.98
2.93
2.92
2.87
2.75
2.70
2.68
2.63
2.59
2.56
2.55
2.52
2.51
2.50
2.49
2.47
2.46
2.44
2.08
1.46
NBoc2
N
N
N
N
O
MeO−P
MeO
AcO
(+/-)-8
1.00
0.98
1.03
6.32
2.05
5.09
1.02
2.87
18.86
9.0 8.5 8.0 7.5 7.0 6.5 6.0 5.5 5.0 4.5 4.0 3.5 3.0 2.5 2.0 1.5
f1 (ppm)

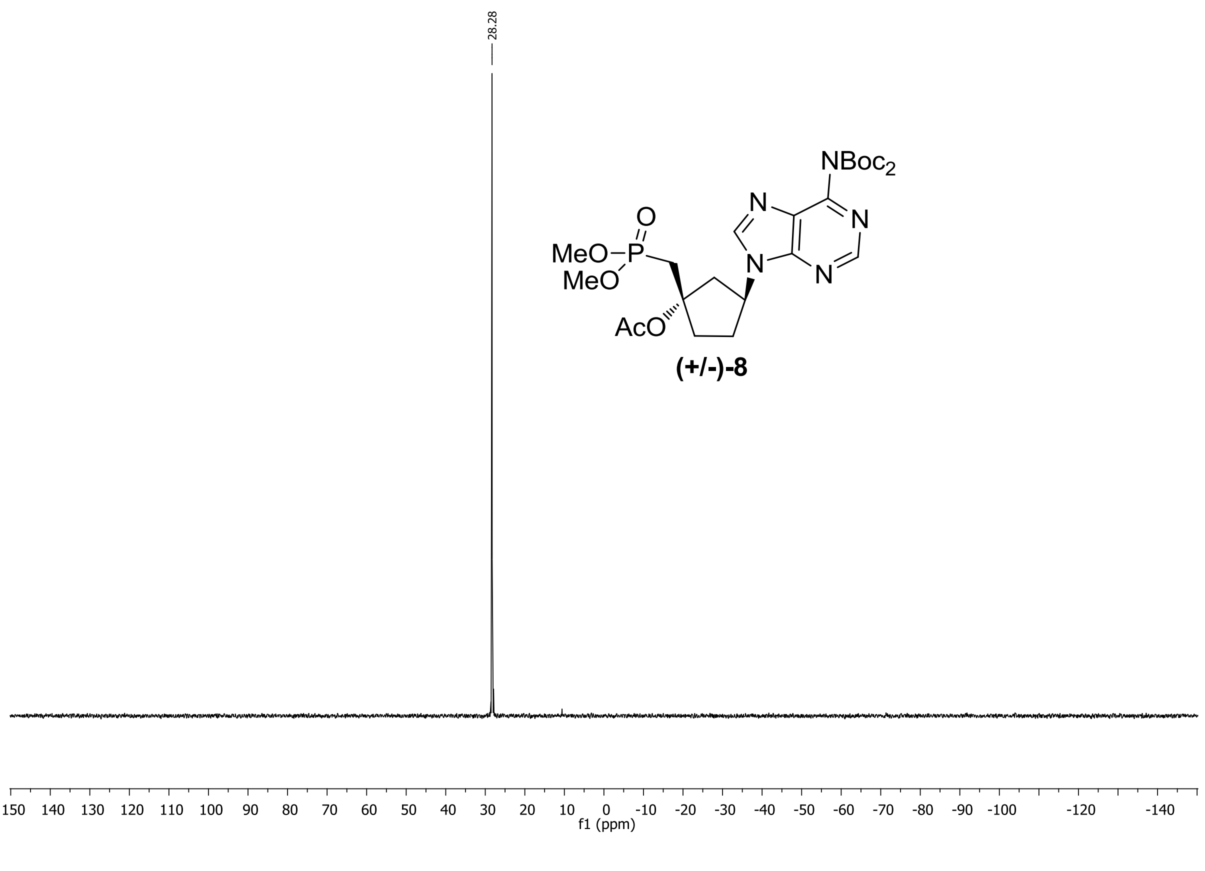
28.28
NBoc2
N
N
N
N
O
MeO−P
MeO
AcO
(+/-)-8
150 140 130 120 110 100 90 80 70 60 50 40 30 20 10 0 -10 -20 -30 -40 -50 -60 -70 -80 -90 -100 -120 -140
f1 (ppm)

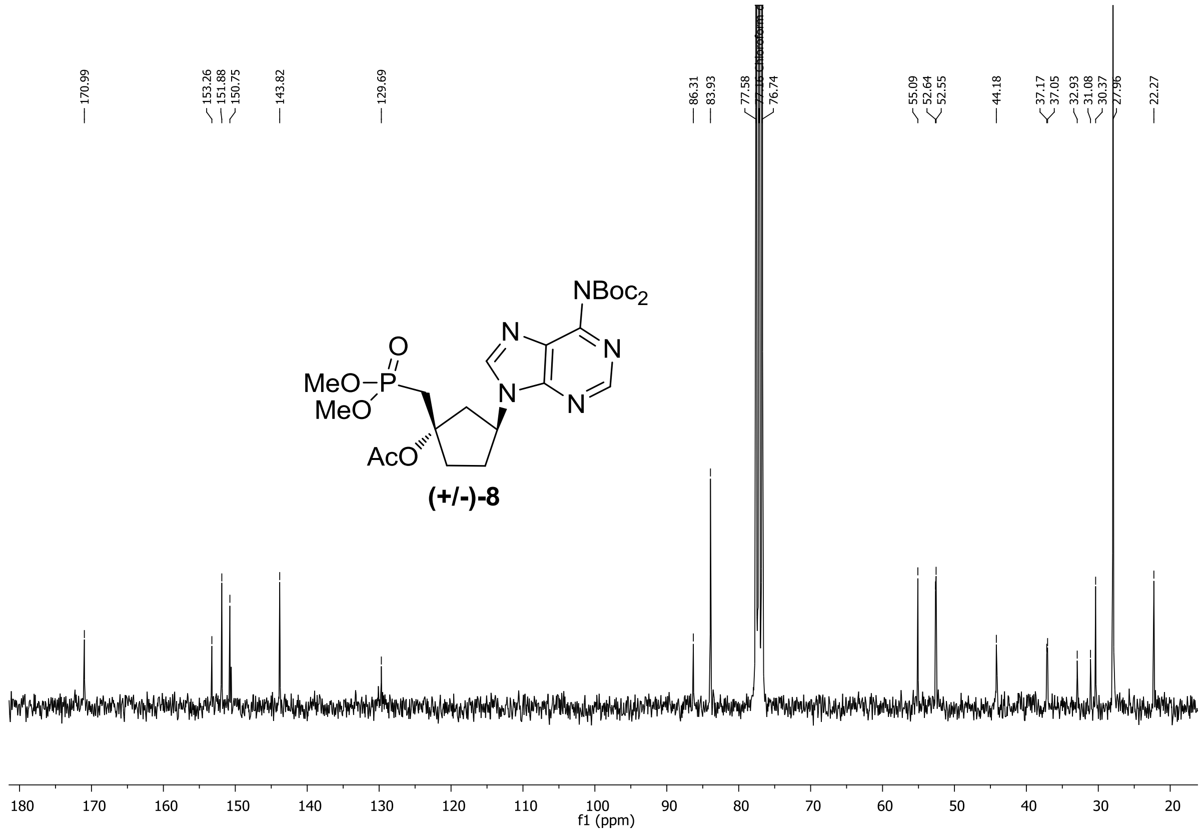
170.99
153.26
151.88
150.75
143.82
129.69
86.31
83.93
77.58
77.16 Chloroform-d
76.74
55.09
52.64
52.55
44.18
37.17
37.05
32.93
31.08
30.37
27.96
22.27
NBoc2
N
N
N
N
O
MeO–P
MeO
AcO
(+/-)-8
180 170 160 150 140 130 120 110 100 90 80 70 60 50 40 30 20
f1 (ppm)

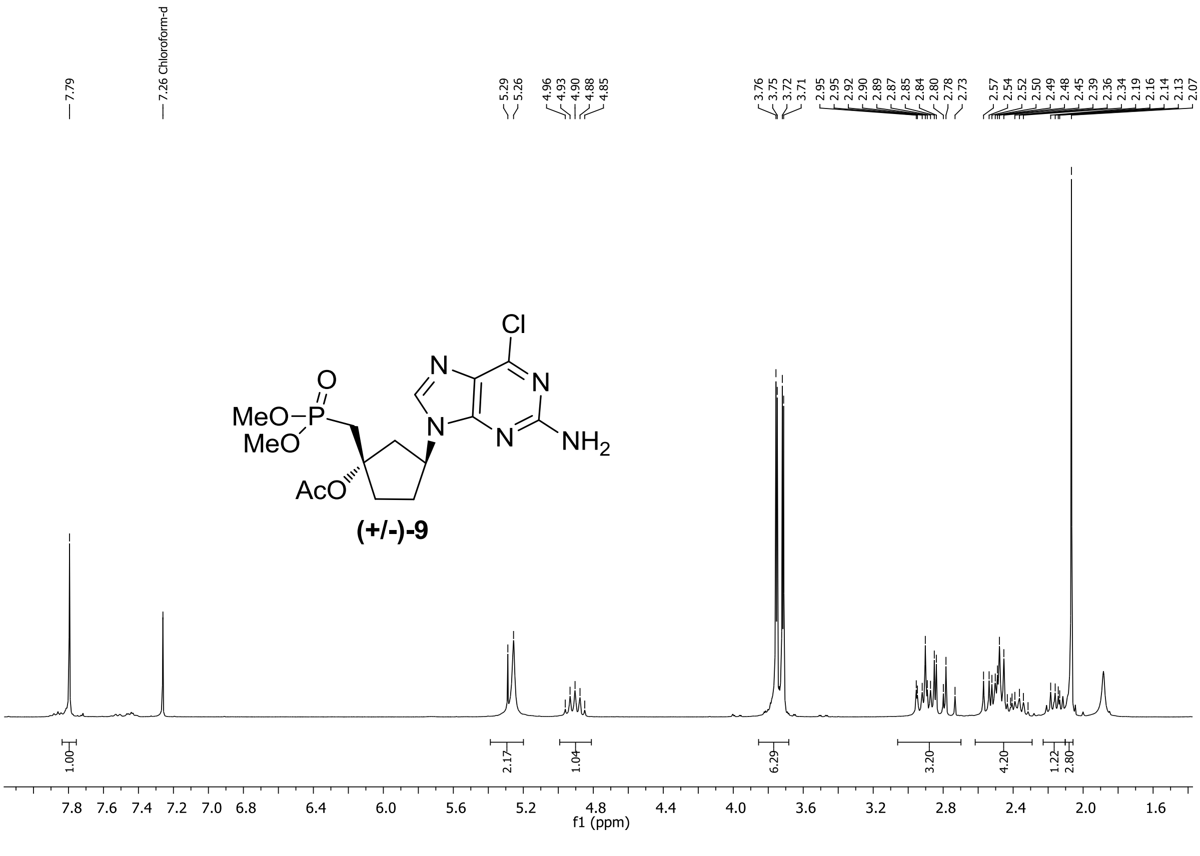
7.79
7.26 Chloroform-d
5.29
5.26
4.96
4.93
4.90
4.88
4.85
3.76
3.75
3.72
3.71
2.95
2.95
2.92
2.90
2.89
2.87
2.85
2.84
2.80
2.78
2.73
2.57
2.54
2.52
2.50
2.49
2.48
2.45
2.39
2.36
2.34
2.19
2.16
2.14
2.13
2.07
Cl
N
N
N
N
NH2
O
MeO–P
MeO
AcO
(+/-)-9
1.00
2.17
1.04
6.29
3.20
4.20
1.22
2.80
7.8 7.6 7.4 7.2 7.0 6.8 6.4 6.0 5.6 5.2 4.8 4.4 4.0 3.6 3.2 2.8 2.4 2.0 1.6
f1 (ppm)

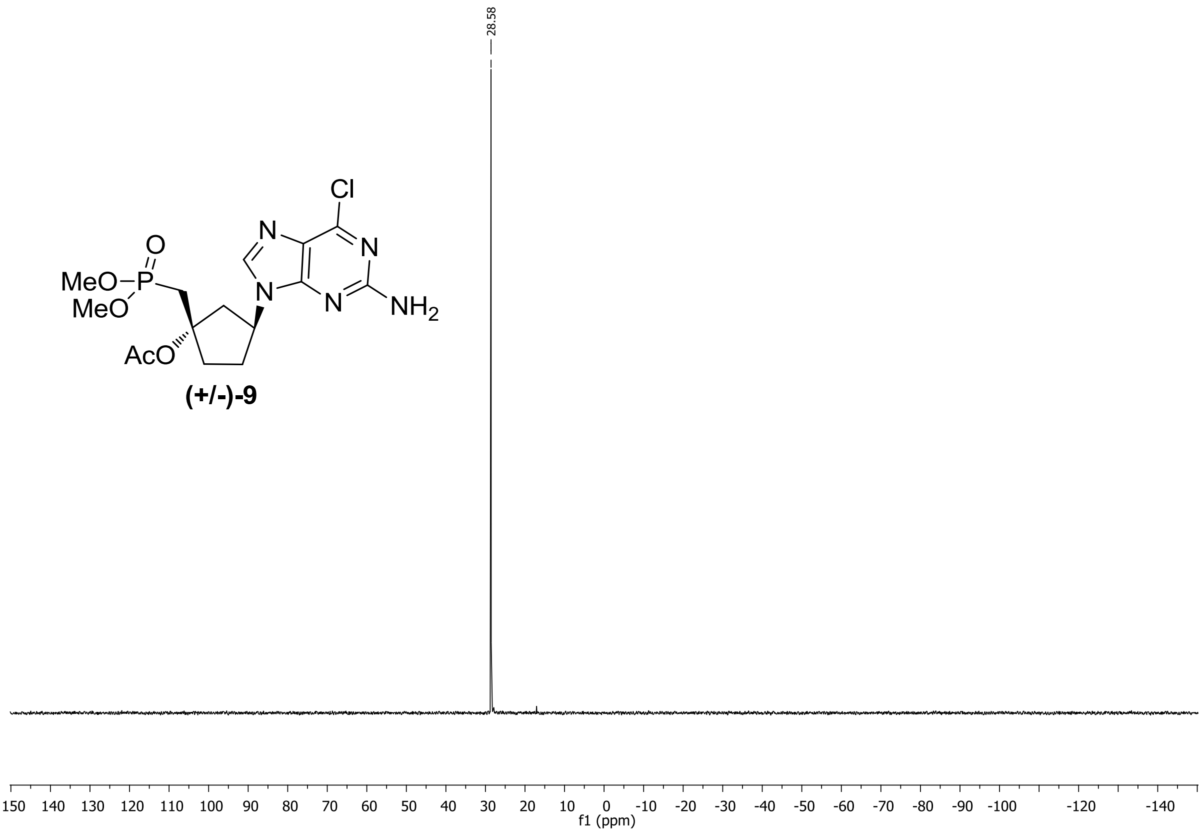
28.58
Cl
N
N
O
MeO−P
MeO
N
N
NH2
AcO
(+/-)-9
150 140 130 120 110 100 90 80 70 60 50 40 30 20 10 0 -10 -20 -30 -40 -50 -60 -70 -80 -90 -100 -120 -140
f1 (ppm)

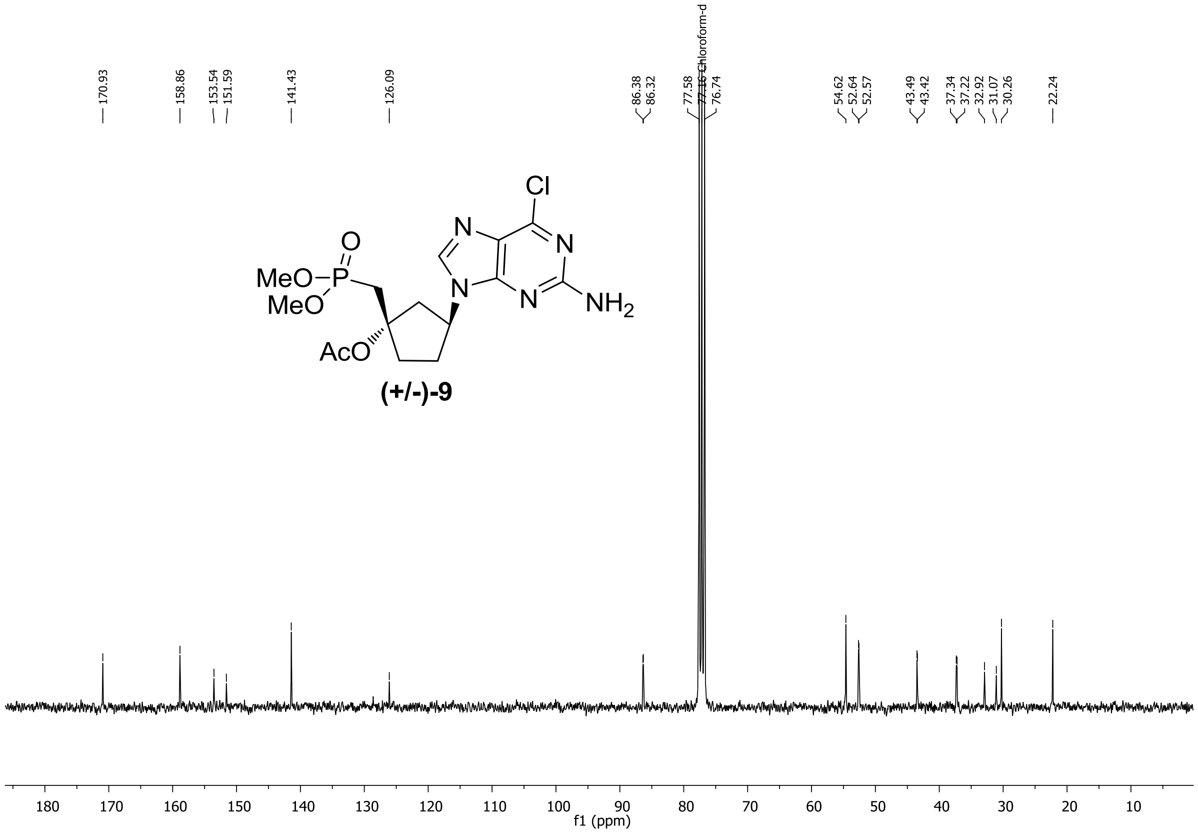
170.93
158.86
153.54
151.59
141.43
126.09
86.38
86.32
77.58
77.16 Chloroform-d
76.74
54.62
52.64
52.57
43.49
43.42
37.34
37.22
32.92
31.07
30.26
22.24
Cl
N
N
O
MeO−P
MeO
N
N
NH2
AcO
(+/-)-9
180 170 160 150 140 130 120 110 100 90 80 70 60 50 40 30 20 10
f1 (ppm)

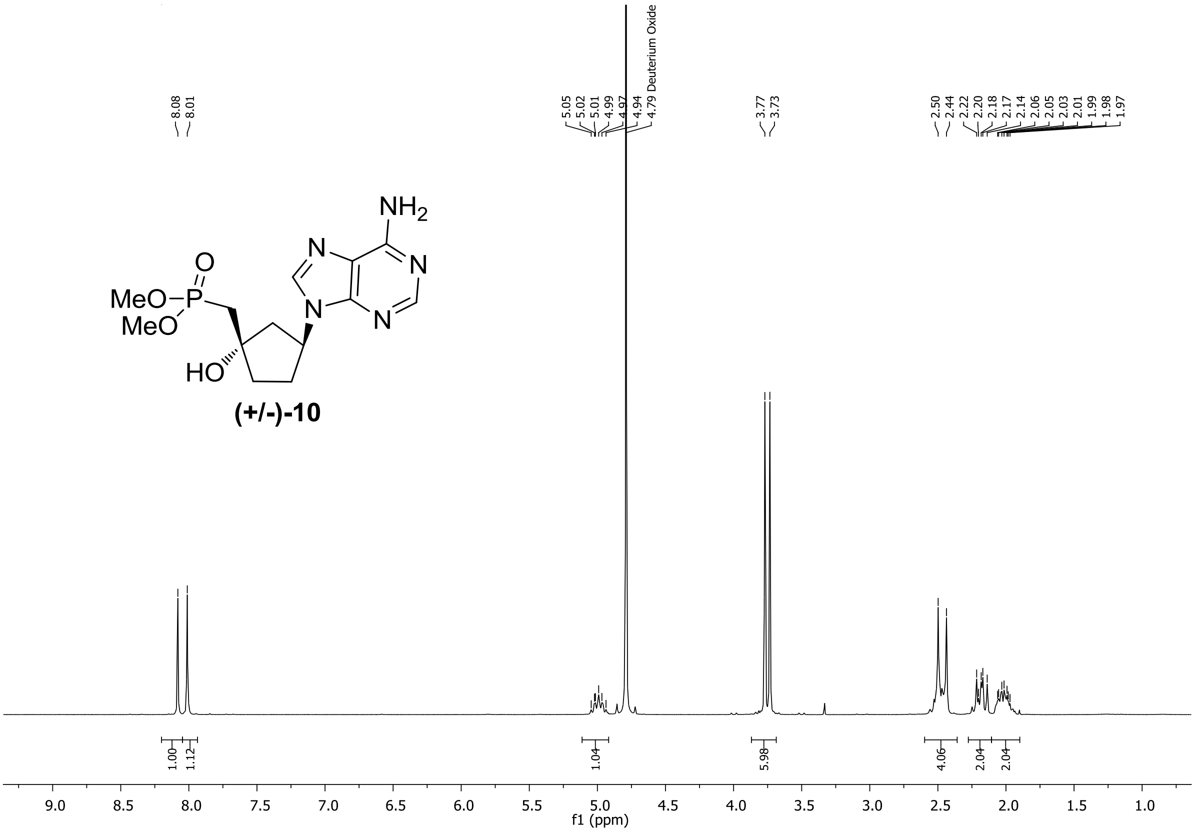
8.08
8.01
5.05
5.02
5.01
4.99
4.97
4.94
4.79 Deuterium Oxide
3.77
3.73
2.50
2.44
2.22
2.20
2.18
2.17
2.14
2.06
2.05
2.03
2.01
1.99
1.98
1.97
NH2
N
N
N
N
O
MeO–P
MeO
HO
(+/-)-10
1.00
1.12
1.04
5.98
4.06
2.04
2.04
f1 (ppm)

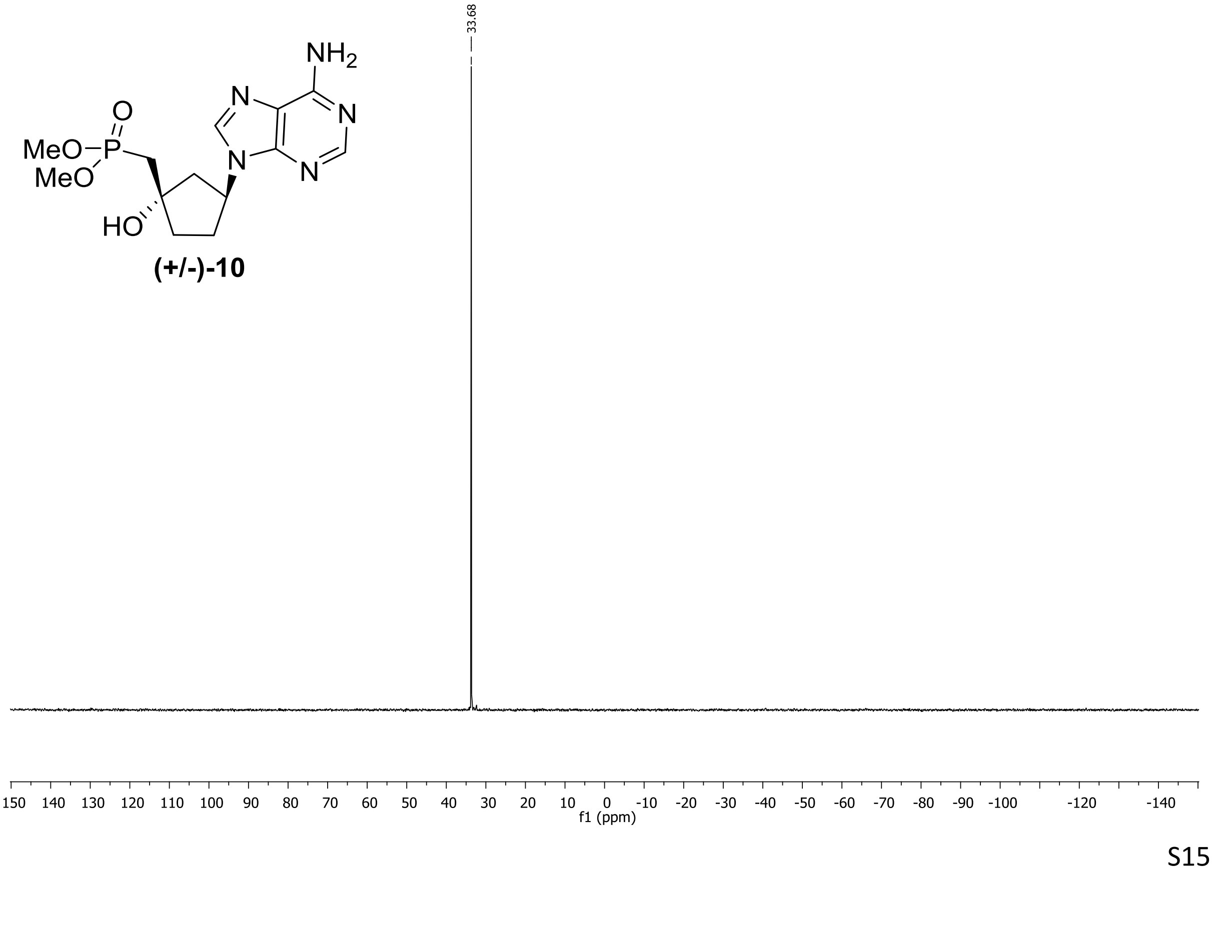
33.68
NH2
N
N
N
N
O
MeO–P
MeO
HO
(+/-)-10
f1 (ppm)

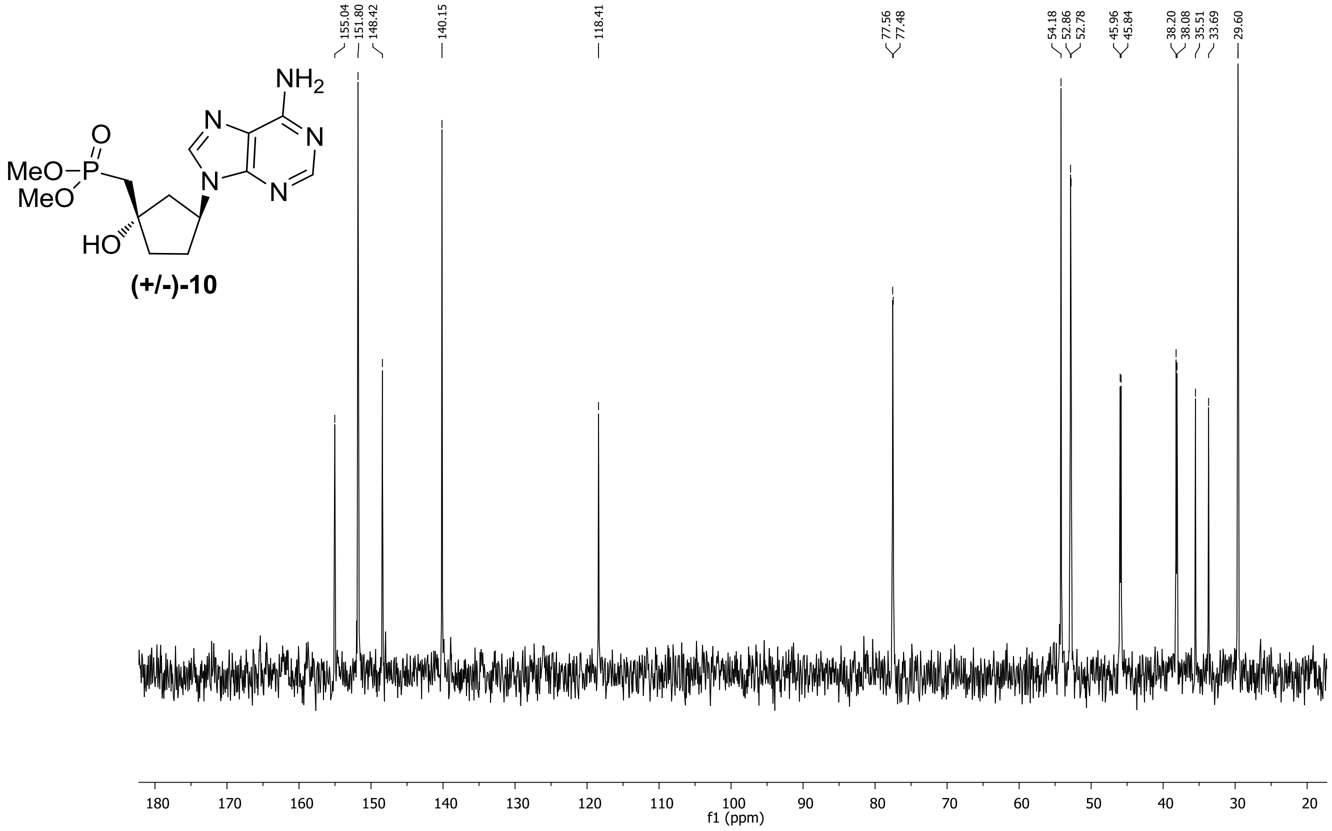
NH2
N
N
N
N
O
MeO-P
MeO
HO
(+/-)-10
155.04
151.80
148.42
140.15
118.41
77.56
77.48
54.18
52.86
52.78
45.96
45.84
38.20
38.08
35.51
33.69
29.60
180 170 160 150 140 130 120 110 100 90 80 70 60 50 40 30 20
f1 (ppm)

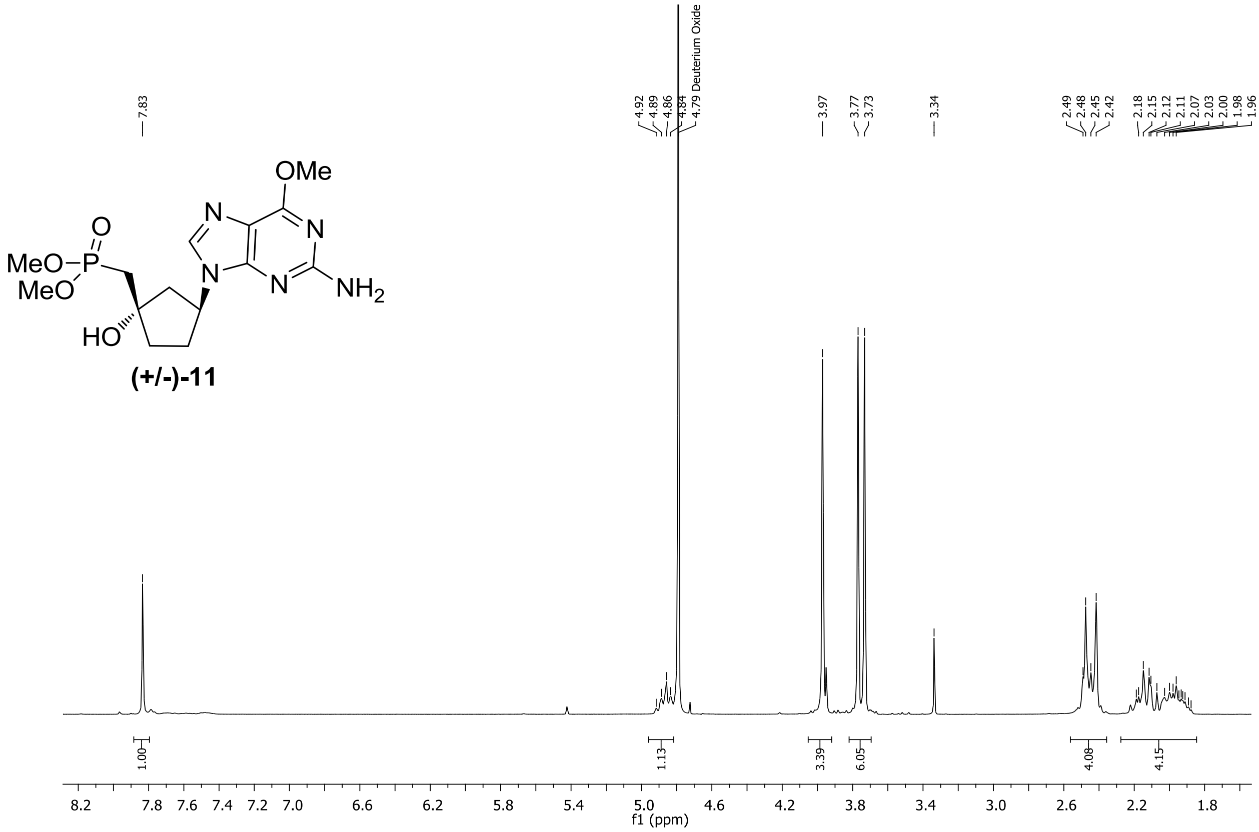

OMe
N
N
N
N
NH2
O
MeO–P
MeO
HO
7.83
4.92
4.89
4.86
4.84
4.79 Deuterium Oxide
3.97
3.77
3.73
3.34
2.49
2.48
2.45
2.42
2.18
2.15
2.12
2.11
2.07
2.03
2.00
1.98
1.96
1.00
1.13
3.39
6.05
4.08
4.15
8.2 7.8 7.6 7.4 7.2 7.0 6.6 6.2 5.8 5.4 5.0 4.6 4.2 3.8 3.4 3.0 2.6 2.2 1.8
f1 (ppm)


**(+/-)-11**

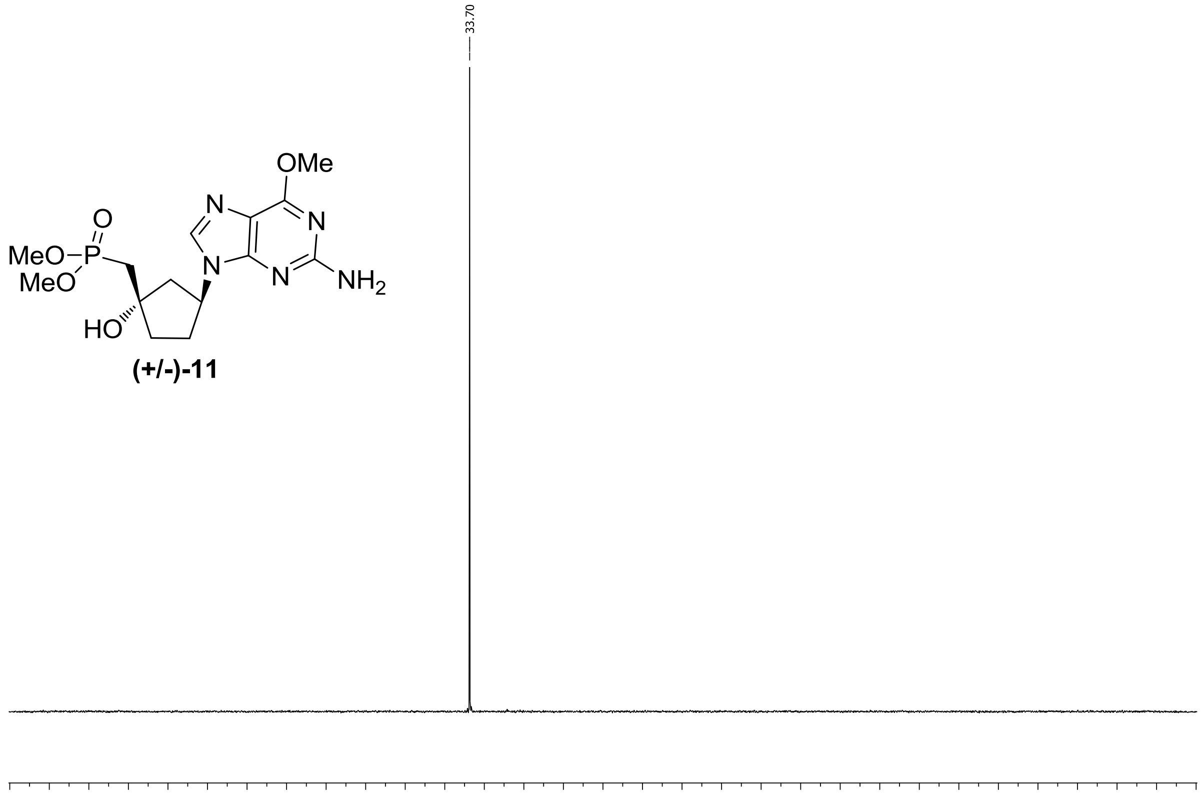

OMe
N
N
N
N
NH2
O
MeO–P
MeO
HO
33.70
150 140 130 120 110 100 90 80 70 60 50 40 30 20 10 0 -10 -20 -30 -40 -50 -60 -70 -80 -90 -100 -120 -140
f1 (ppm)


**(+/-)-11**

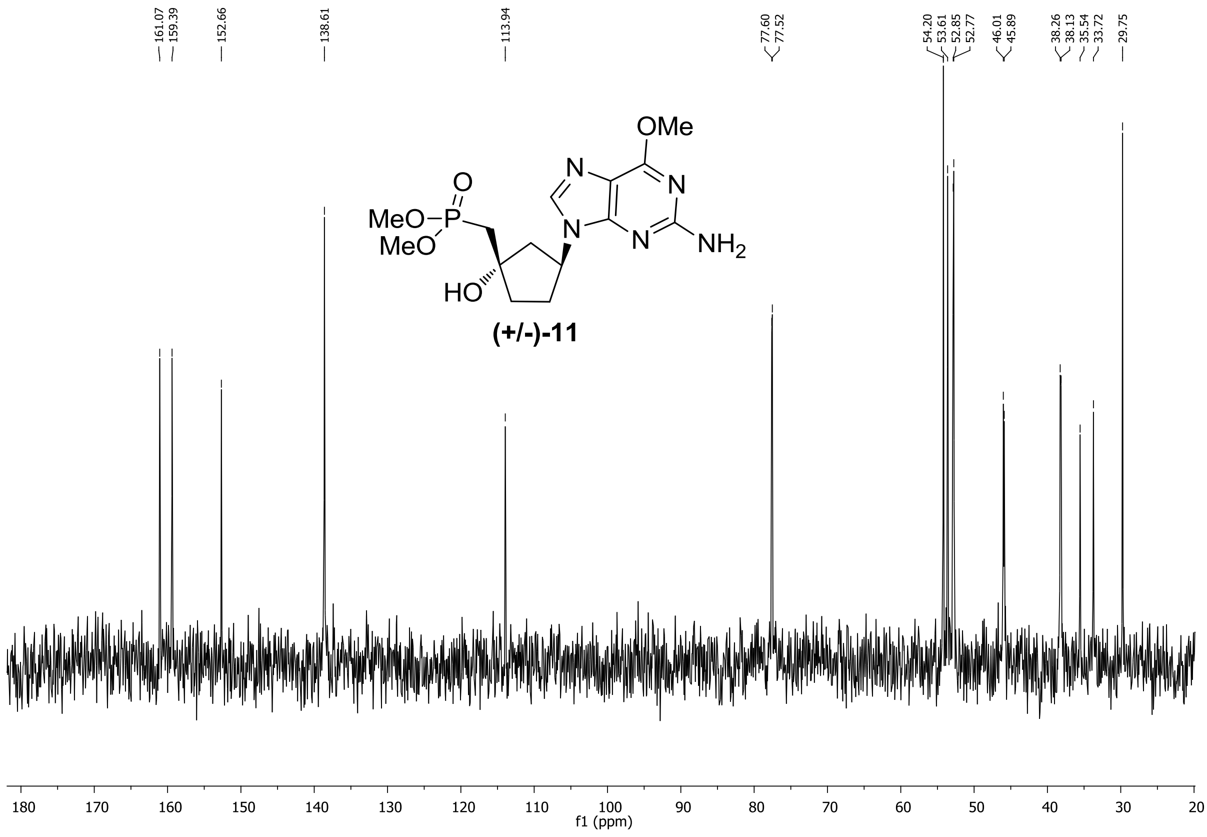
161.07
159.39
152.66
138.61
113.94
77.60
77.52
54.20
53.61
52.85
52.77
46.01
45.89
38.26
38.13
35.54
33.72
29.75
OMe
N
N
O
MeO−P
MeO
N
N
NH2
HO
(+/-)-11
180 170 160 150 140 130 120 110 100 90 80 70 60 50 40 30 20
f1 (ppm)

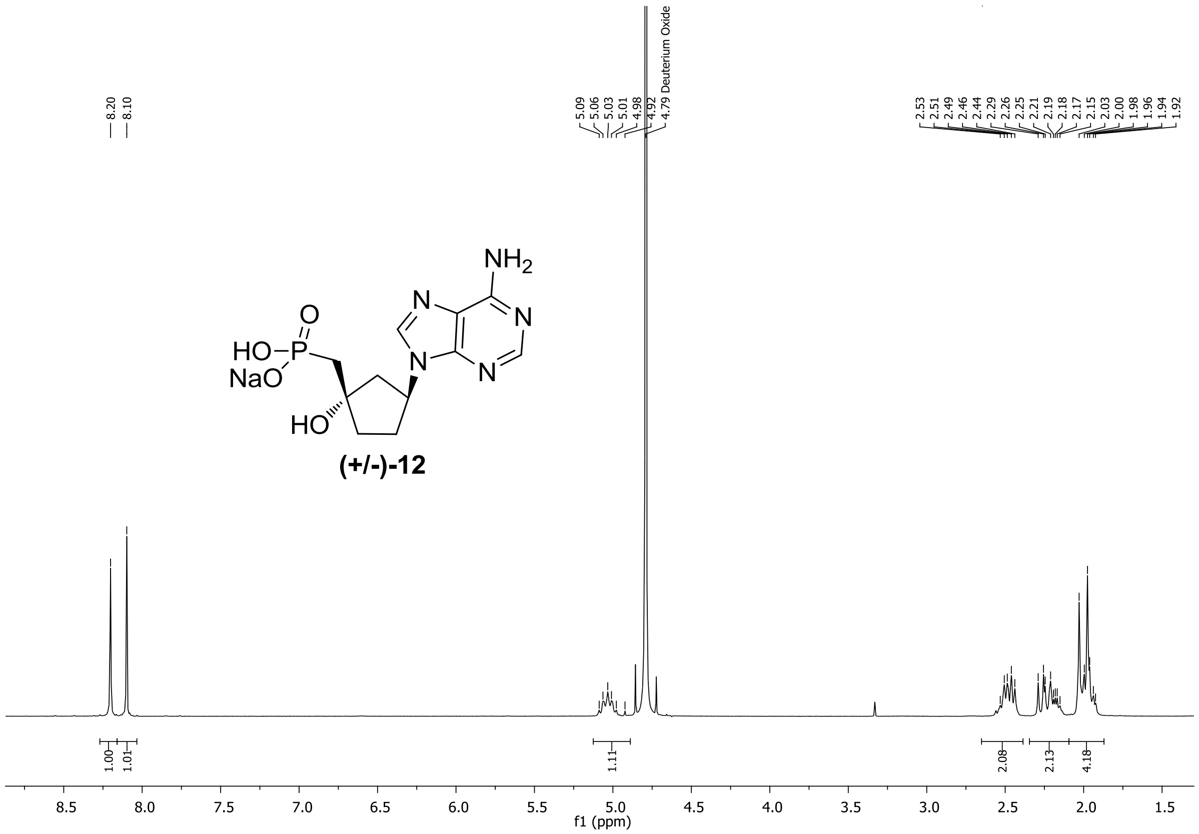
8.20
8.10
5.09
5.06
5.03
5.01
4.98
4.92
4.79 Deuterium Oxide
2.53
2.51
2.49
2.46
2.44
2.29
2.26
2.25
2.21
2.19
2.18
2.17
2.15
2.03
2.00
1.98
1.96
1.94
1.92
NH2
N
N
O
HO−P
NaO
N
N
HO
(+/-)-12
1.00
1.01
1.11
2.08
2.13
4.18
8.5 8.0 7.5 7.0 6.5 6.0 5.5 5.0 4.5 4.0 3.5 3.0 2.5 2.0 1.5
f1 (ppm)

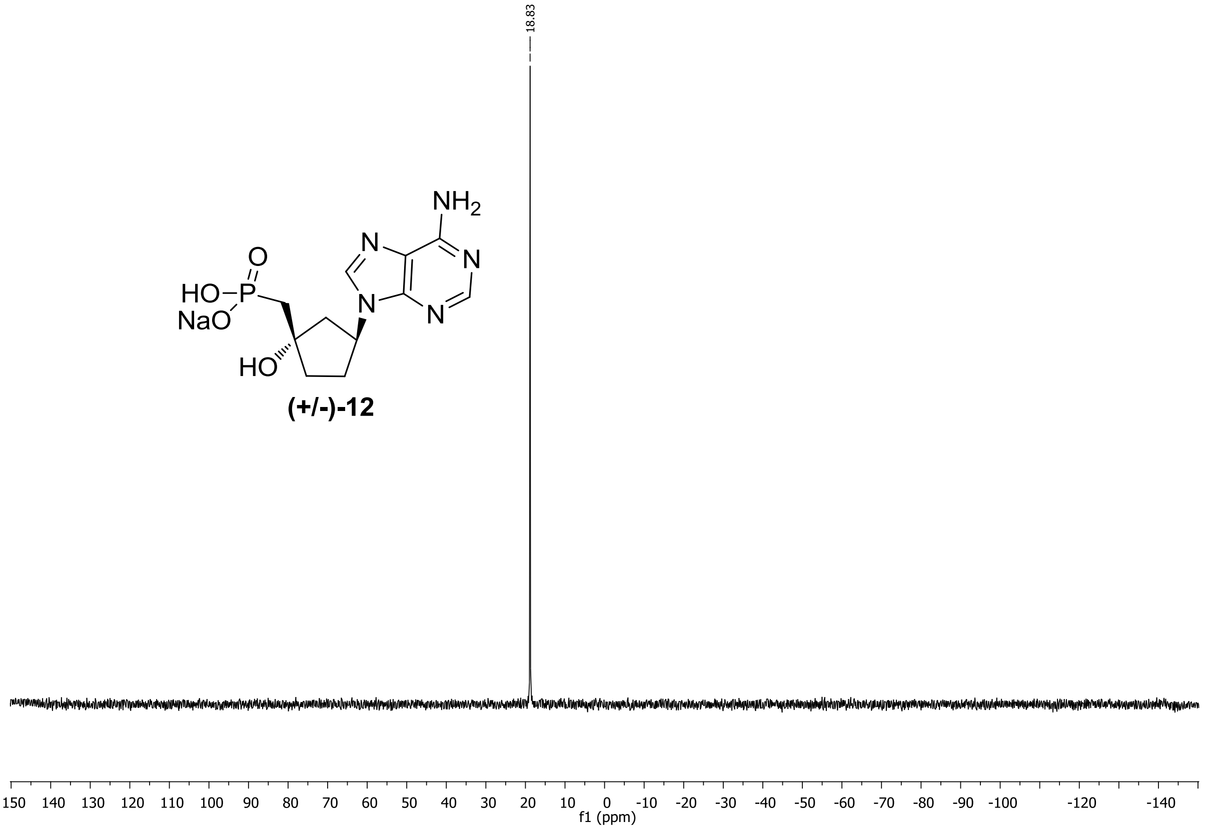
18.83
NH2
O
HO-P
NaO
N
HO
(+/-)-12
f1 (ppm)

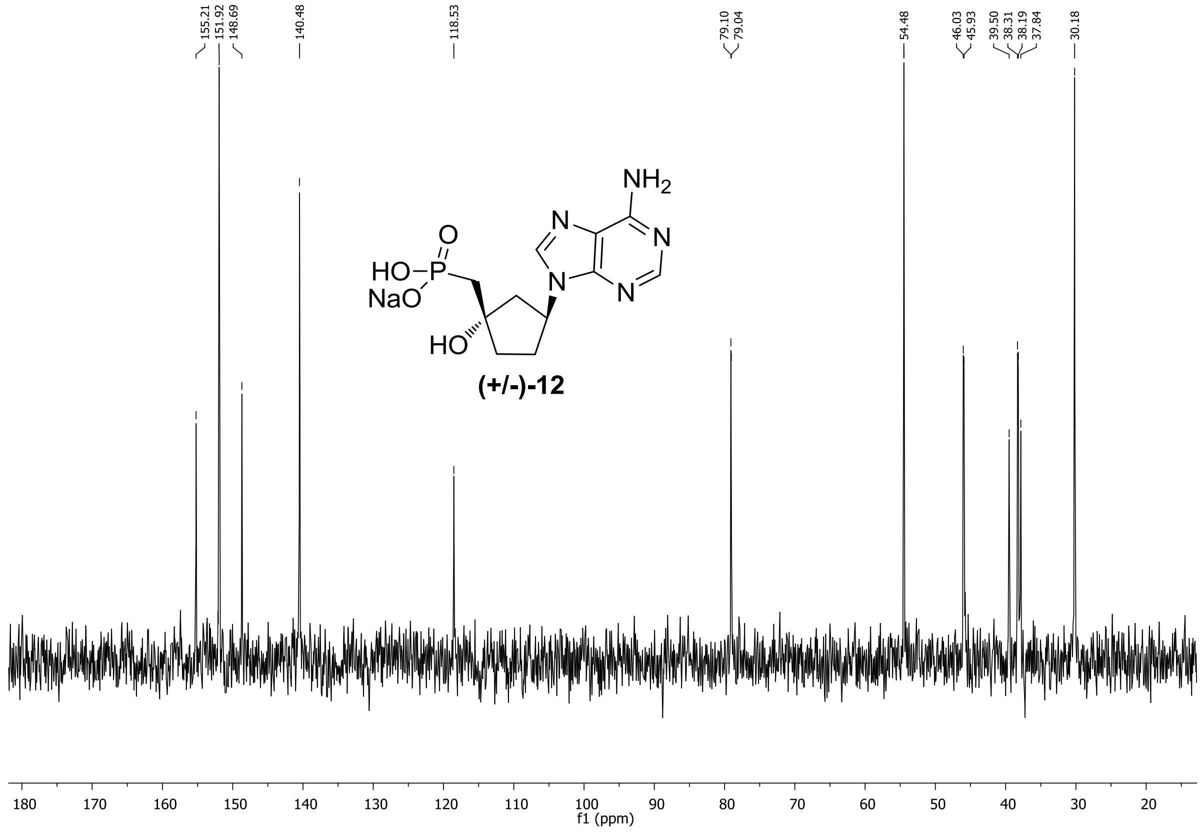
155.21
151.92
148.69
140.48
118.53
79.10
79.04
54.48
46.03
45.93
39.50
38.31
38.19
37.84
30.18
NH2
O
HO-P
NaO
N
HO
(+/-)-12
f1 (ppm)

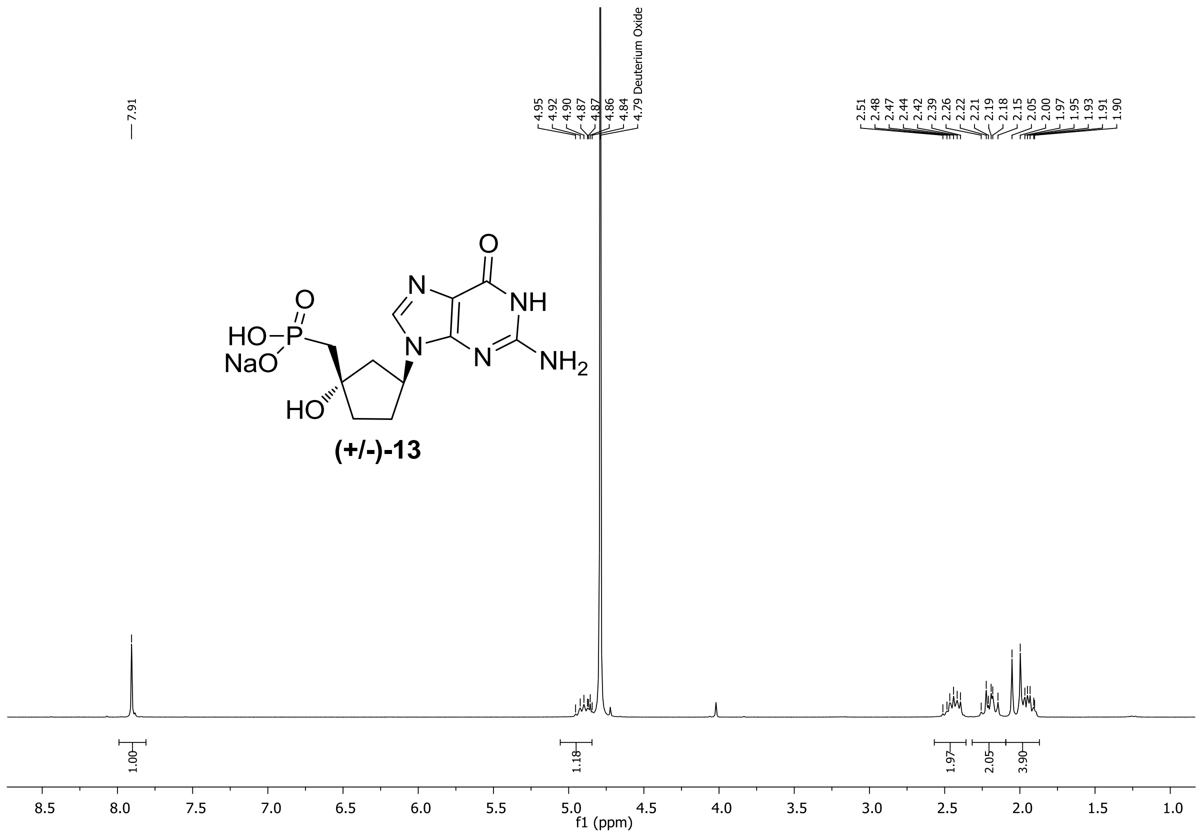
7.91
4.95
4.92
4.90
4.87
4.87
4.86
4.84
4.79 Deuterium Oxide
2.51
2.48
2.47
2.44
2.42
2.39
2.26
2.22
2.21
2.19
2.18
2.15
2.05
2.00
1.97
1.95
1.93
1.91
1.90
1.00
1.18
1.97
2.05
3.90
f1 (ppm)

(+/-)-13

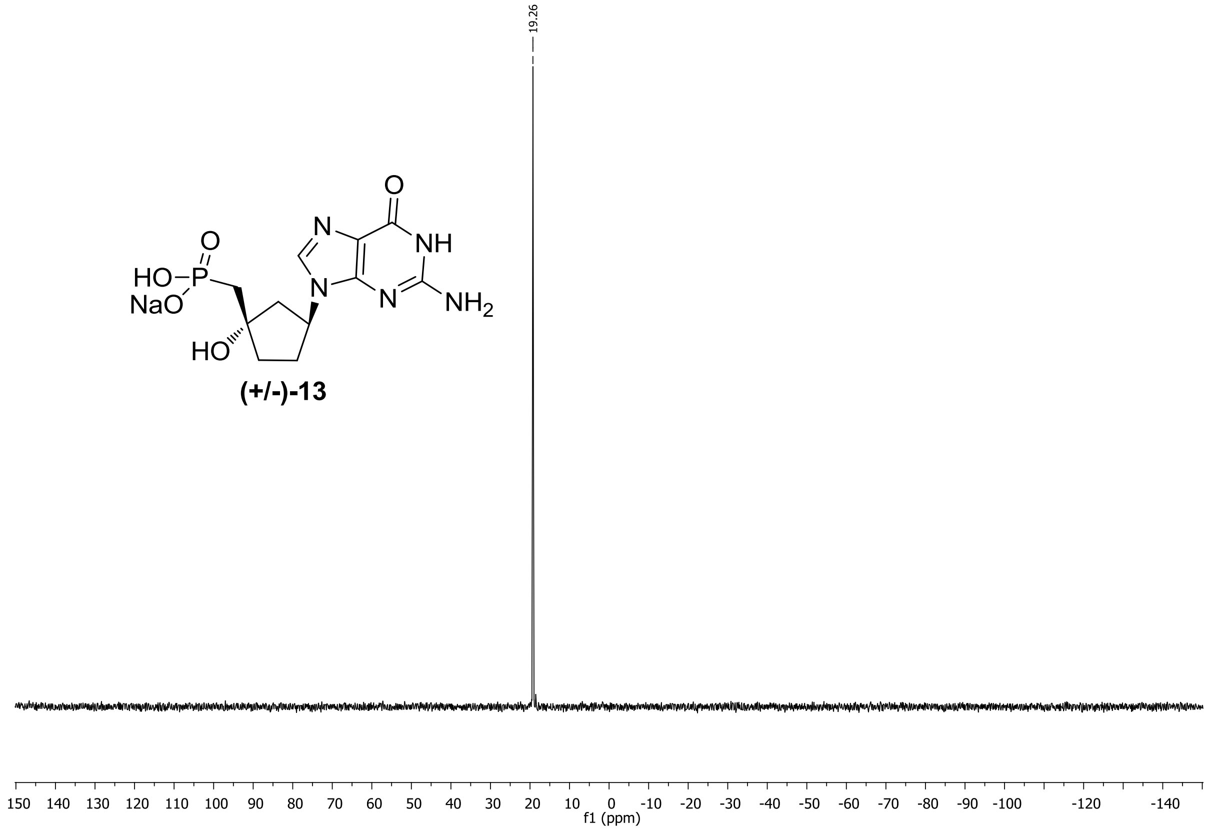
19.26
f1 (ppm)

(+/-)-13

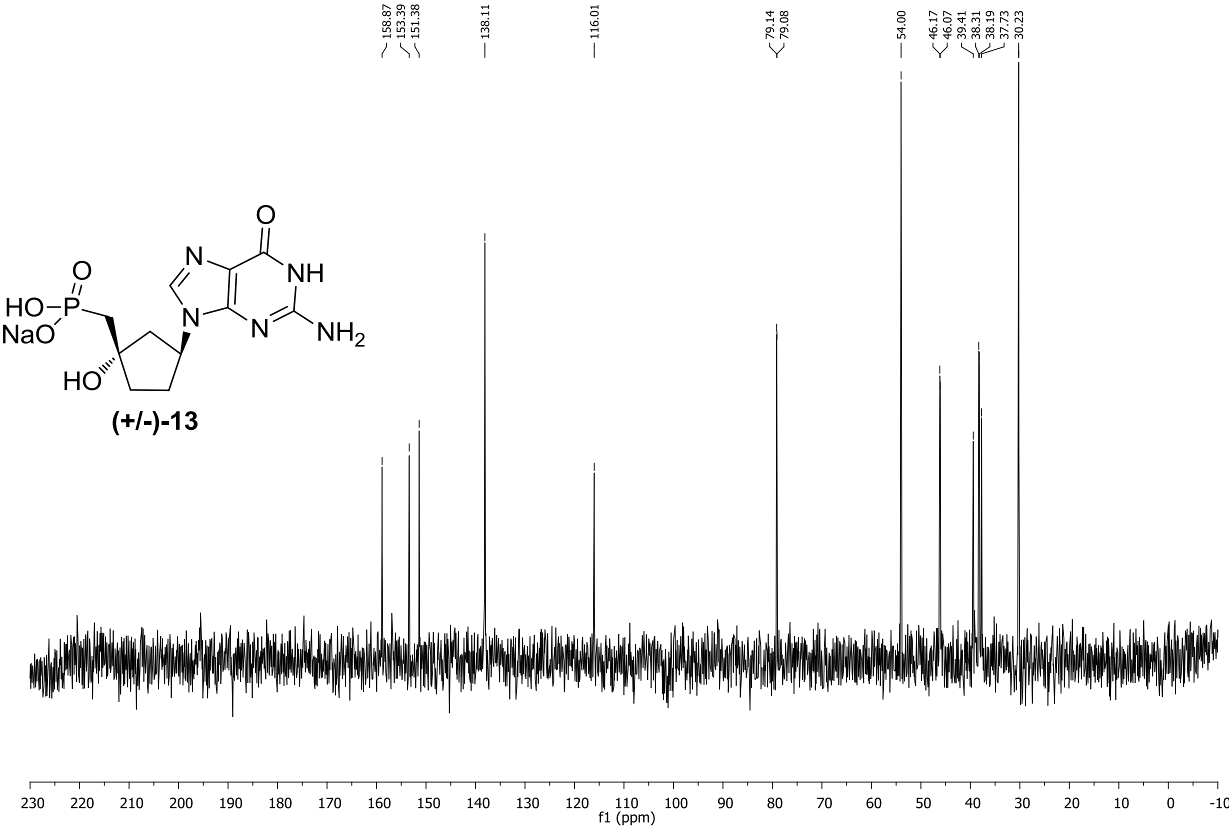
158.87
153.39
151.38
138.11
116.01
79.14
79.08
54.00
46.17
46.07
39.41
38.31
38.19
37.73
30.23
O
N
NH
HO−P
NaO
N
N
NH2
HO
(+/-)-13
230 220 210 200 190 180 170 160 150 140 130 120 110 100 90 80 70 60 50 40 30 20 10 0 -10
f1 (ppm)

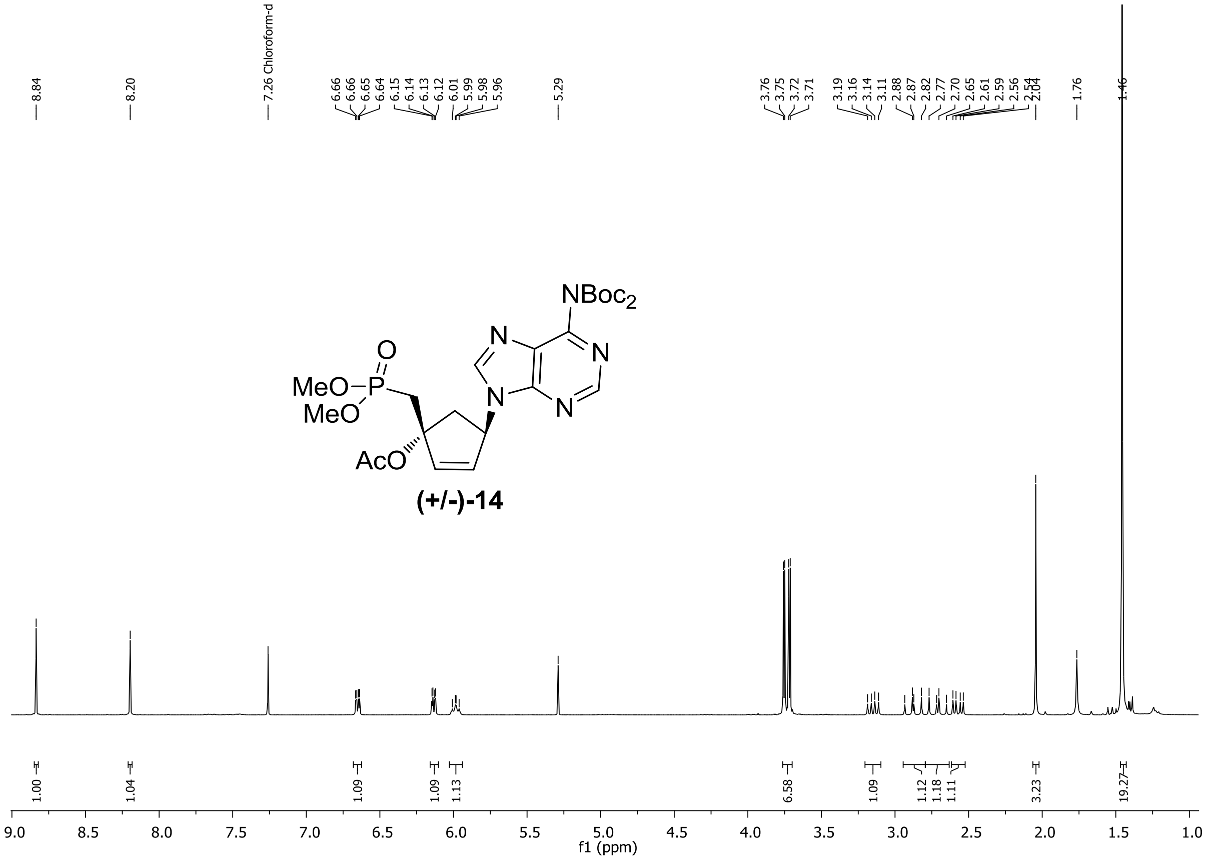
8.84
8.20
7.26 Chloroform-d
6.66
6.66
6.65
6.64
6.15
6.14
6.13
6.12
6.01
5.99
5.98
5.96
5.29
3.76
3.75
3.72
3.71
3.19
3.16
3.14
3.11
2.88
2.87
2.82
2.77
2.70
2.65
2.61
2.59
2.56
2.54
2.04
1.76
1.46
NBoc2
N
N
O
MeO−P
MeO
N
N
AcO
(+/-)-14
1.00
1.04
1.09
1.09
1.13
6.58
1.09
1.12
1.18
1.11
3.23
19.27
9.0 8.5 8.0 7.5 7.0 6.5 6.0 5.5 5.0 4.5 4.0 3.5 3.0 2.5 2.0 1.5 1.0
f1 (ppm)

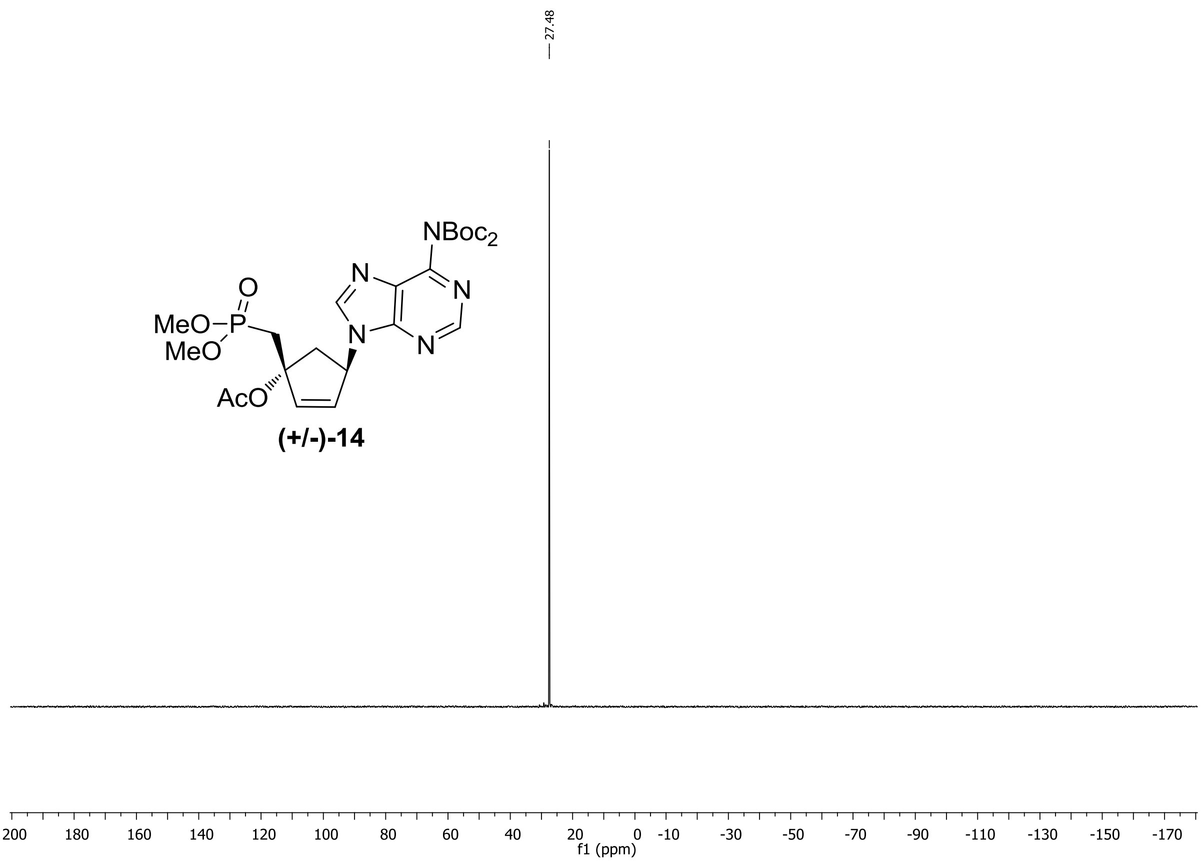
27.48
NBoc2
MeO
MeO
AcO
(+/-)-14
f1 (ppm)

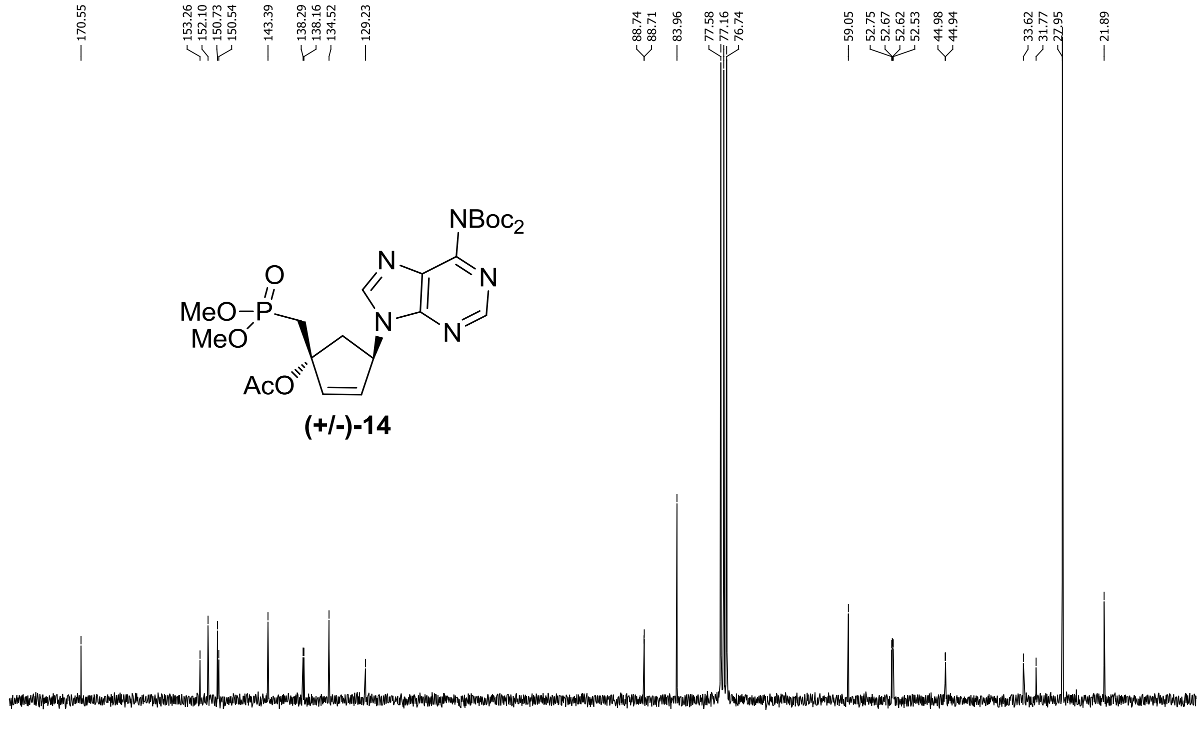
170.55
153.26
152.10
150.73
150.54
143.39
138.29
138.16
134.52
129.23
88.74
88.71
83.96
77.58
77.16
76.74
59.05
52.75
52.67
52.62
52.53
44.98
44.94
33.62
31.77
27.95
21.89
NBoc2
MeO
MeO
AcO
(+/-)-14
f1 (ppm)

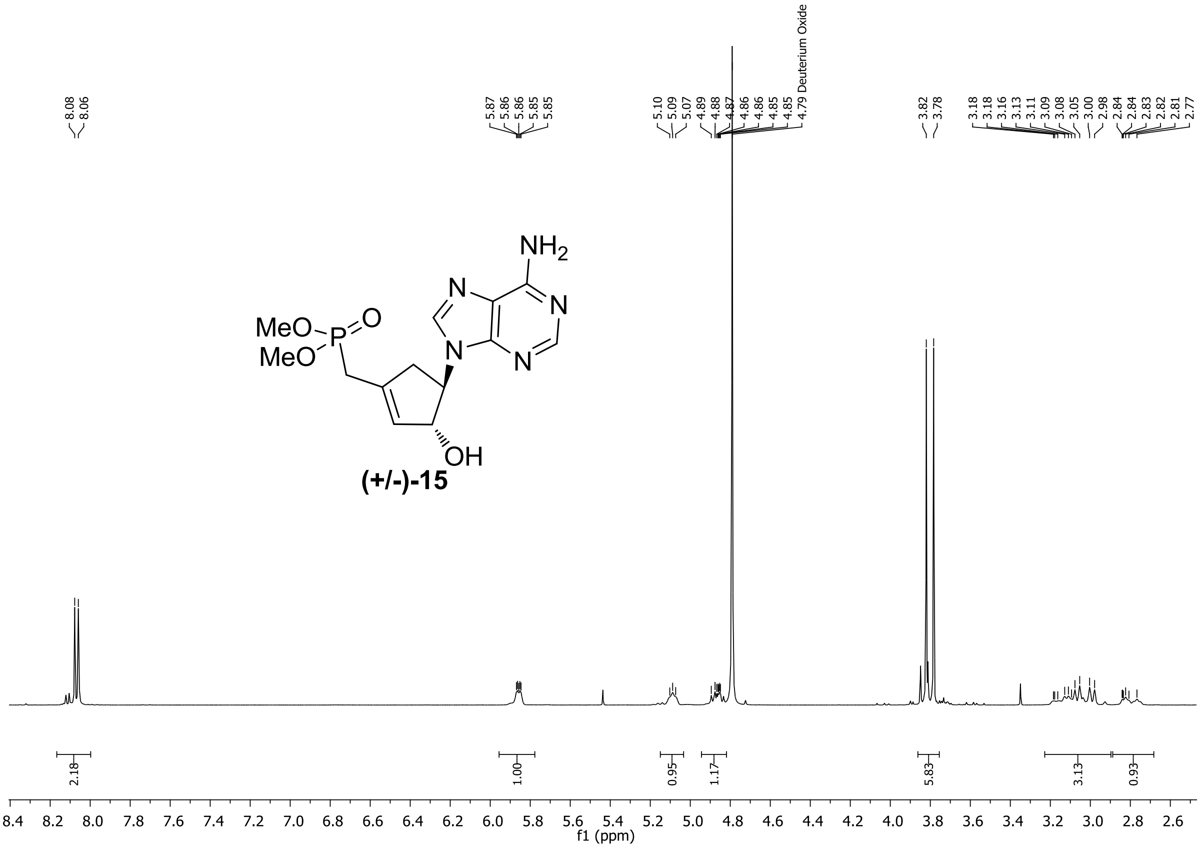
8.08
8.06
5.87
5.86
5.86
5.85
5.85
5.10
5.09
5.07
4.89
4.88
4.87
4.86
4.86
4.85
4.85
4.79 Deuterium Oxide
3.82
3.78
3.18
3.18
3.16
3.13
3.11
3.09
3.08
3.05
3.00
2.98
2.84
2.84
2.83
2.82
2.81
2.77
NH2
N
N
N
N
MeO
MeO
P
O
OH
(+/-)-15
2.18
1.00
0.95
1.17
5.83
3.13
0.93
8.4 8.2 8.0 7.8 7.6 7.4 7.2 7.0 6.8 6.6 6.4 6.2 6.0 5.8 5.6 5.4 5.2 5.0 4.8 4.6 4.4 4.2 4.0 3.8 3.6 3.4 3.2 3.0 2.8 2.6
f1 (ppm)

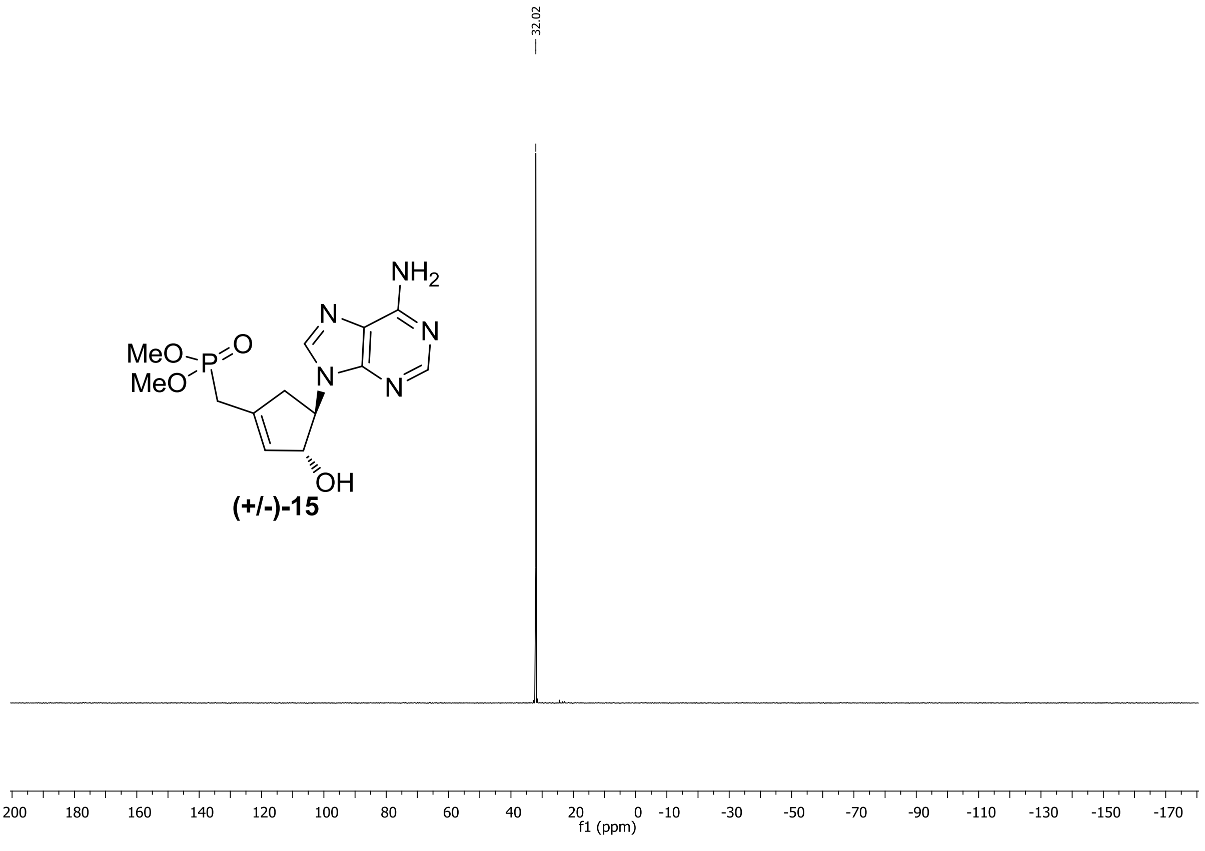
32.02
NH2
N
N
N
N
MeO
MeO
P
O
OH
(+/-)-15
200 180 160 140 120 100 80 60 40 20 0 -10 -30 -50 -70 -90 -110 -130 -150 -170
f1 (ppm)

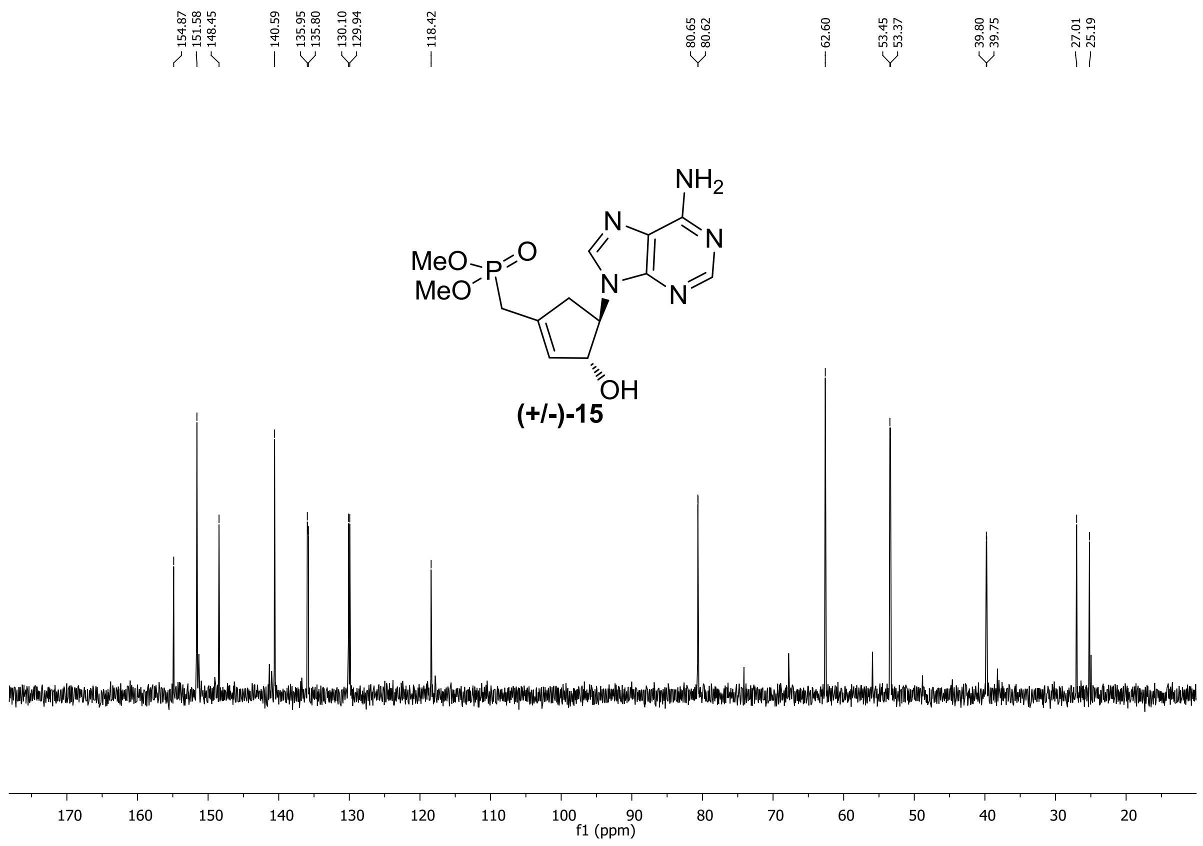
154.87
151.58
148.45
140.59
135.95
135.80
130.10
129.94
118.42
80.65
80.62
62.60
53.45
53.37
39.80
39.75
27.01
25.19
NH2
N
N
MeO
P
O
MeO
N
N
OH
(+/-)-15
170 160 150 140 130 120 110 100 90 80 70 60 50 40 30 20
f1 (ppm)

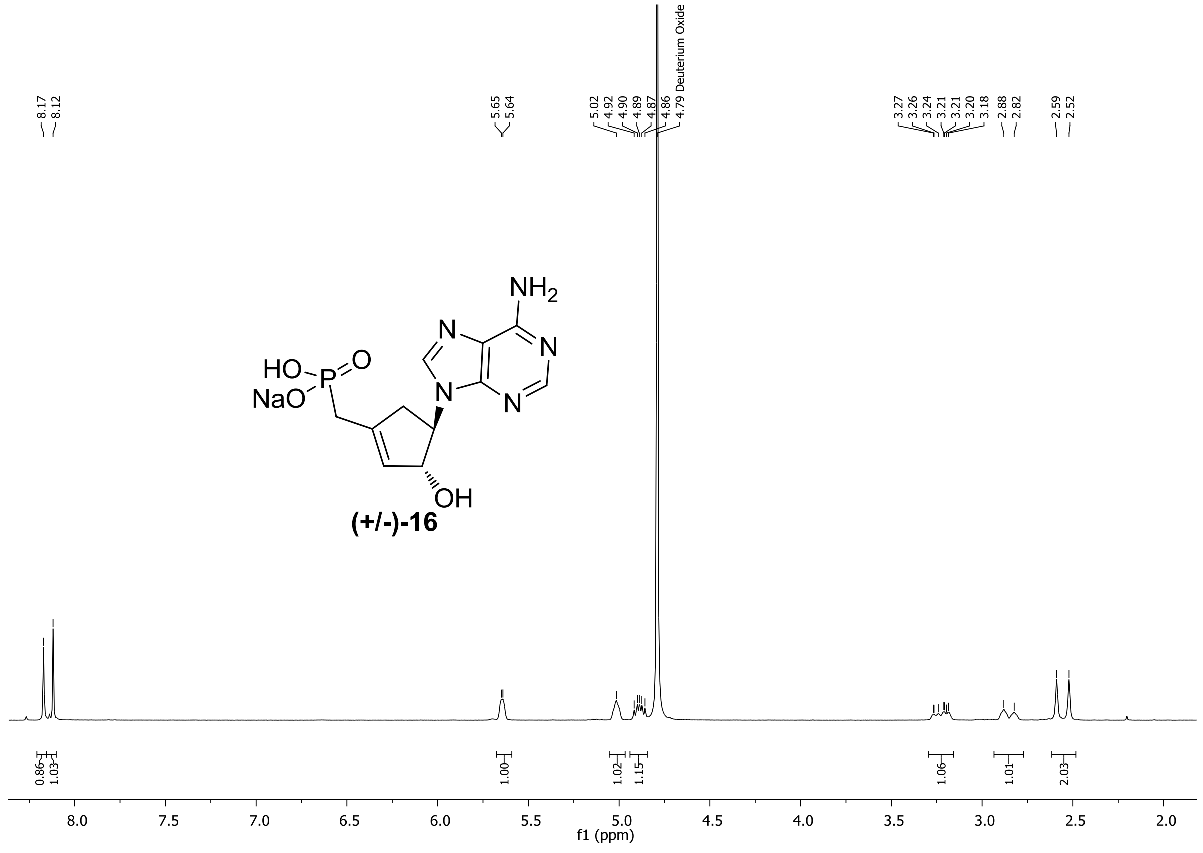
8.17
8.12
5.65
5.64
5.02
4.92
4.90
4.89
4.87
4.86
4.79 Deuterium Oxide
3.27
3.26
3.24
3.21
3.21
3.20
3.18
2.88
2.82
2.59
2.52
NH2
N
N
HO
P
O
NaO
N
N
OH
(+/-)-16
0.86
1.03
1.00
1.02
1.15
1.06
1.01
2.03
8.0 7.5 7.0 6.5 6.0 5.5 5.0 4.5 4.0 3.5 3.0 2.5 2.0
f1 (ppm)

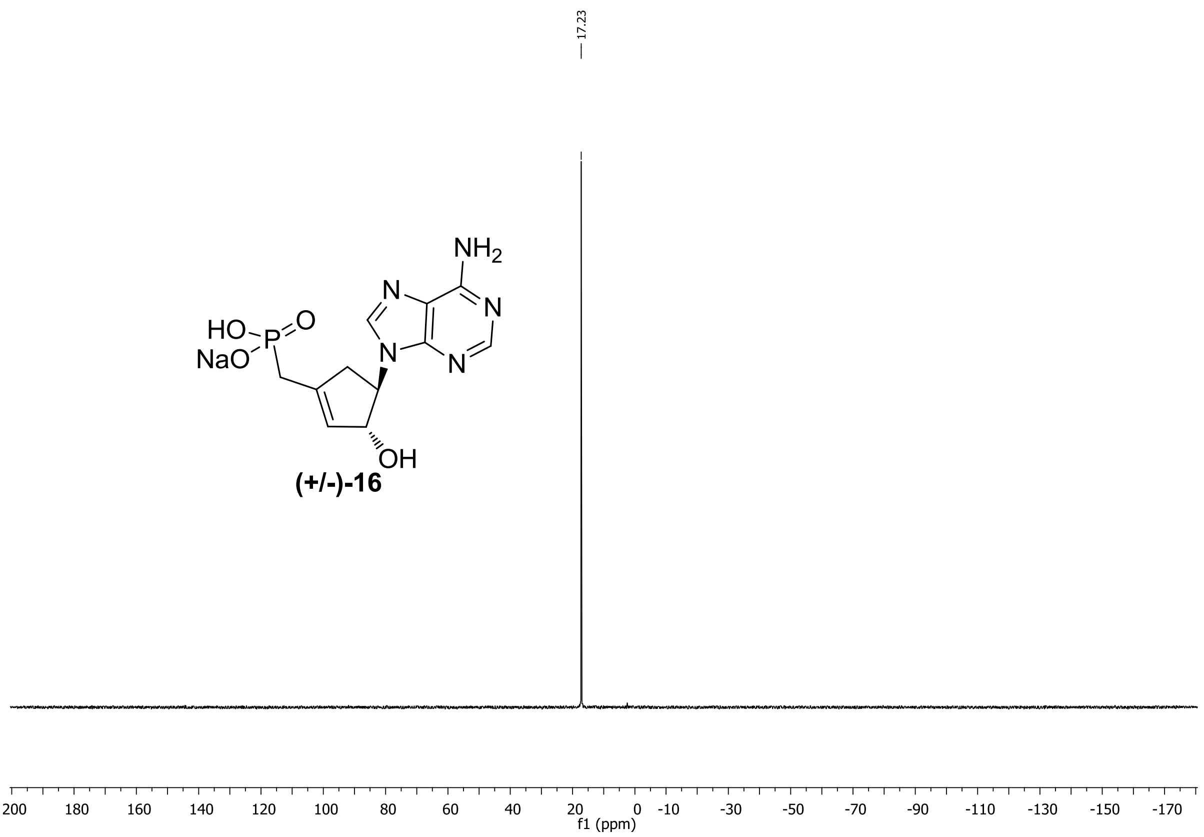
17.23
NH2
HO
P
O
NaO
N
OH
(+/-)-16
200 180 160 140 120 100 80 60 40 20 0 -10 -30 -50 -70 -90 -110 -130 -150 -170
f1 (ppm)

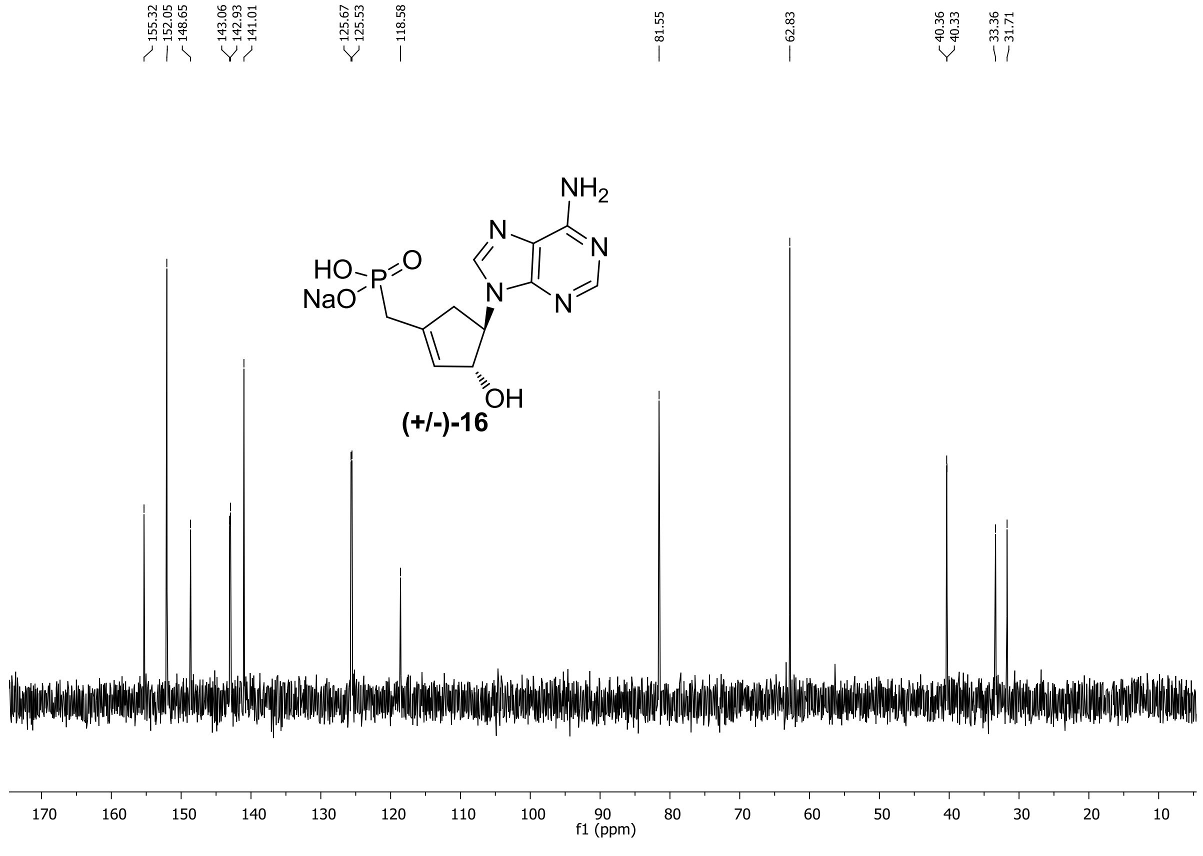
155.32
152.05
148.65
143.06
142.93
141.01
125.67
125.53
118.58
81.55
62.83
40.36
40.33
33.36
31.71
NH2
HO
P
O
NaO
N
OH
(+/-)-16
170 160 150 140 130 120 110 100 90 80 70 60 50 40 30 20 10
f1 (ppm)

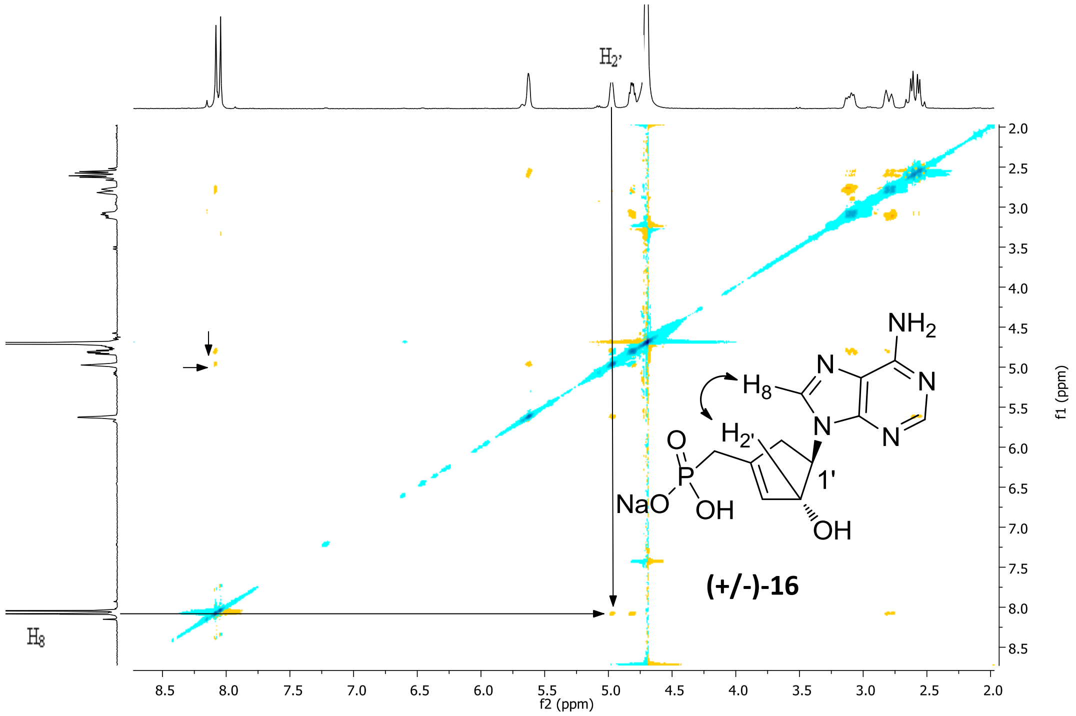
H2'
H8
NH2
N
N
N
N
O
P
NaO
OH
1'
OH
(+/-)-16
f2 (ppm)
f1 (ppm)
8.5
8.0
7.5
7.0
6.5
6.0
5.5
5.0
4.5
4.0
3.5
3.0
2.5
2.0

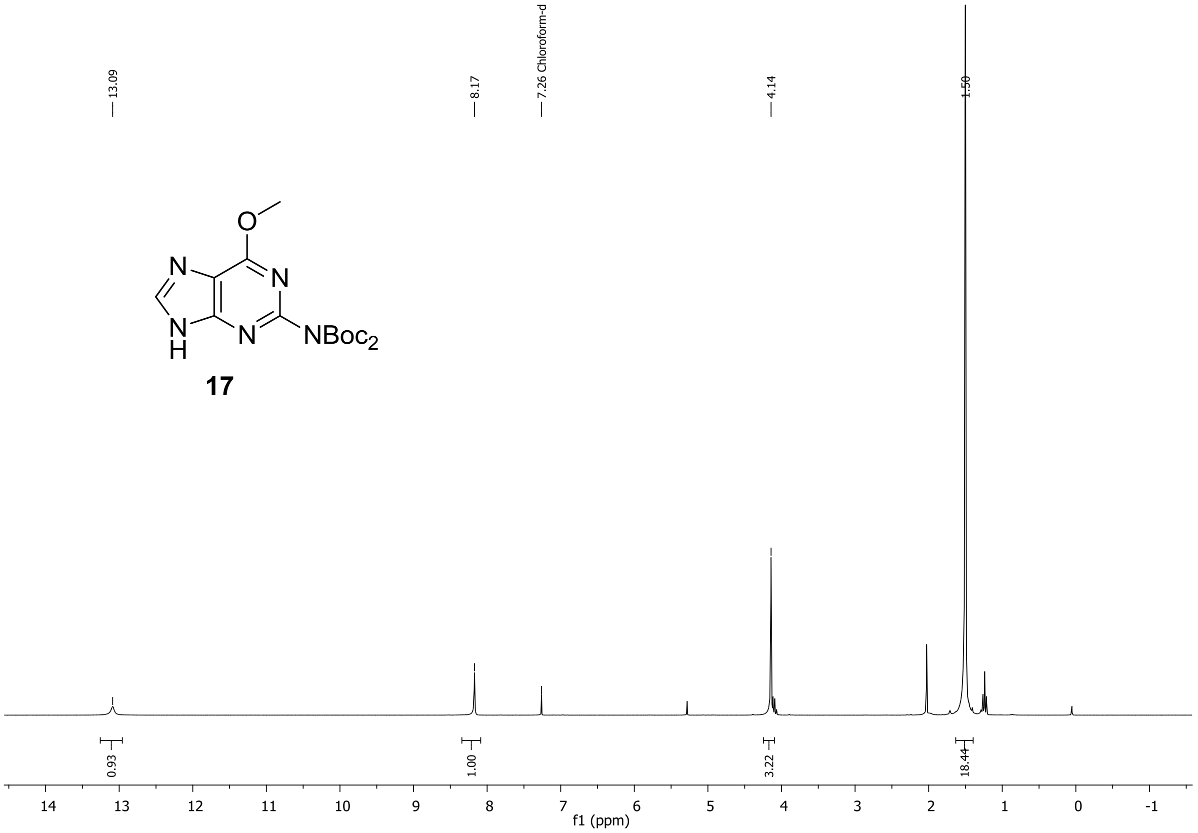
13.09
8.17
7.26 Chloroform-d
4.14
1.50
N
N
N
N
H
O
NBoc2
17
0.93
1.00
3.22
18.44
14 13 12 11 10 9 8 7 6 5 4 3 2 1 0 -1
f1 (ppm)

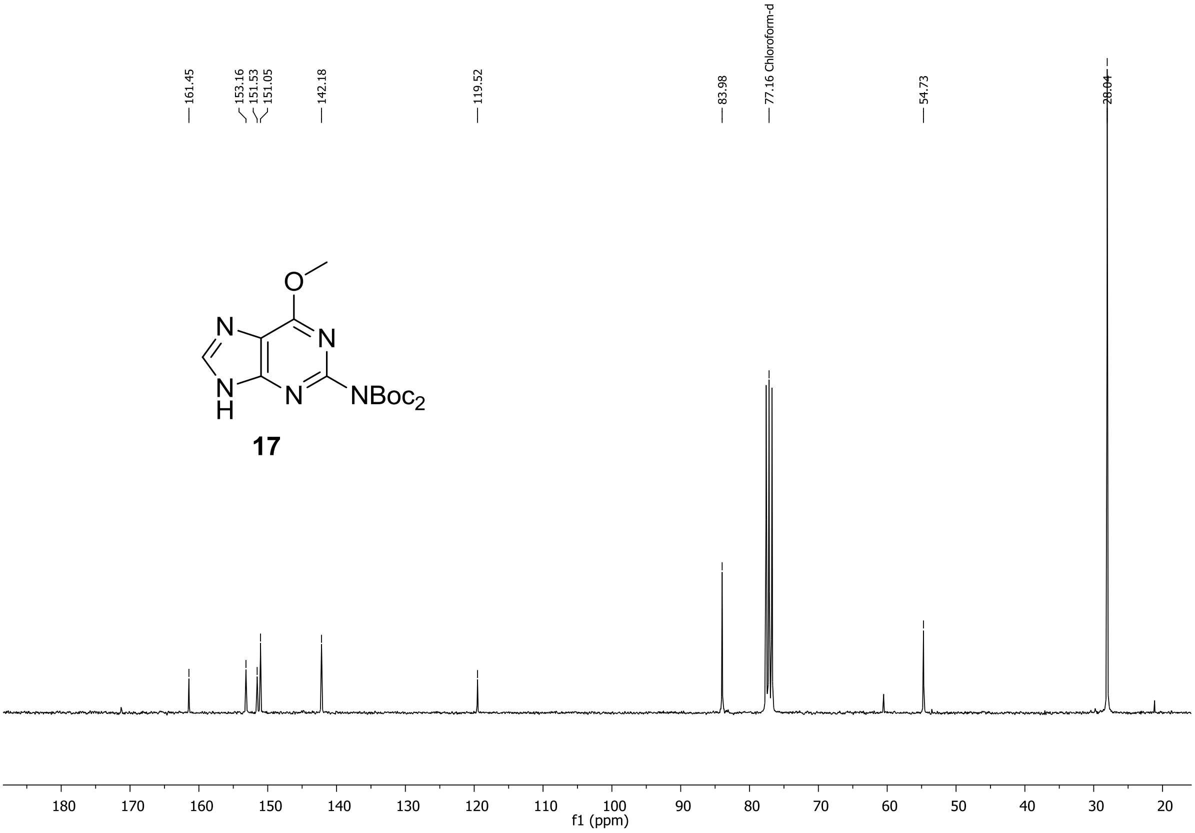
161.45
153.16
151.53
151.05
142.18
119.52
83.98
77.16 Chloroform-d
54.73
28.04
N
N
N
N
H
O
NBoc2
17
180 170 160 150 140 130 120 110 100 90 80 70 60 50 40 30 20
f1 (ppm)

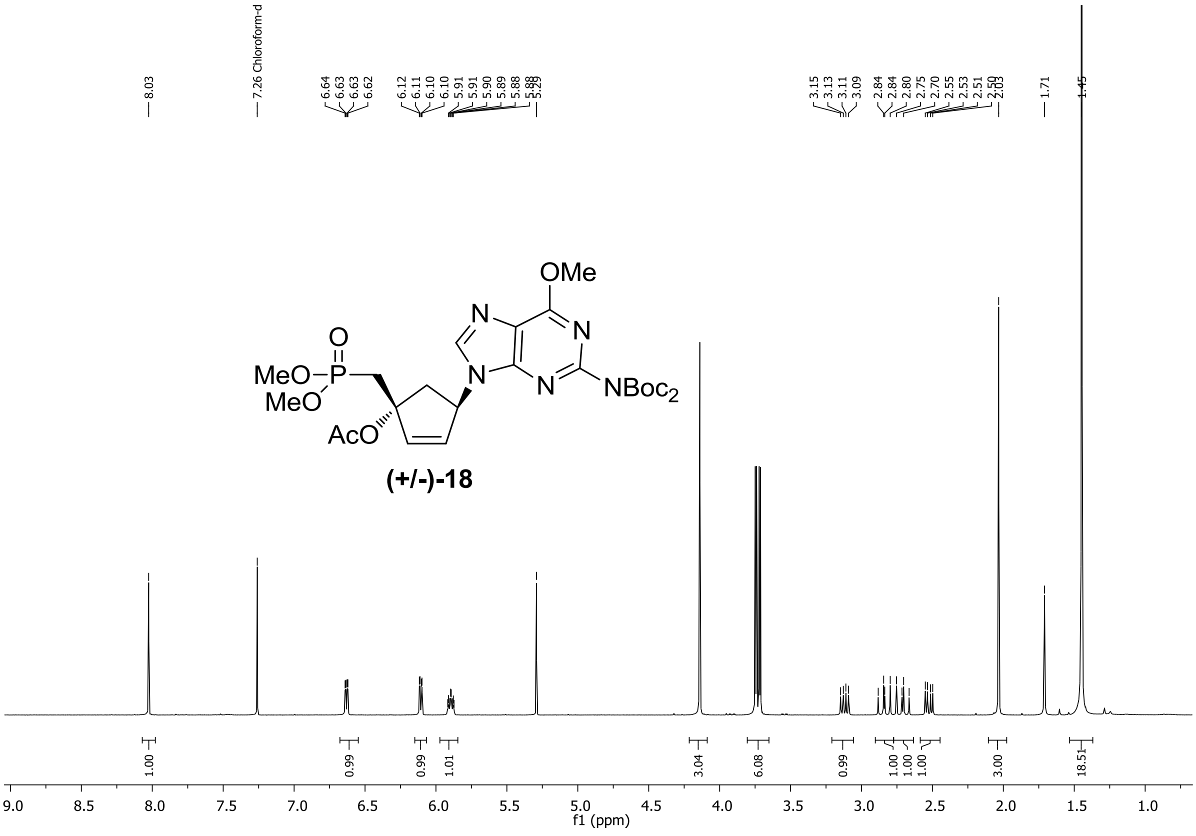
8.03
7.26 Chloroform-d
6.64
6.63
6.63
6.62
6.12
6.11
6.10
6.10
5.91
5.91
5.90
5.89
5.88
3.15
3.13
3.11
3.09
2.84
2.84
2.80
2.75
2.70
2.55
2.53
2.51
2.03
1.71
1.45
OMe
N
N
O
MeO
P
MeO
N
N
NBoc2
AcO
1.00
0.99
0.99
1.01
3.04
6.08
0.99
1.00
1.00
1.00
3.00
18.51
9.0
8.5
8.0
7.5
7.0
6.5
6.0
5.5
5.0
4.5
4.0
3.5
3.0
2.5
2.0
1.5
1.0
f1 (ppm)

**(+/-)-18**

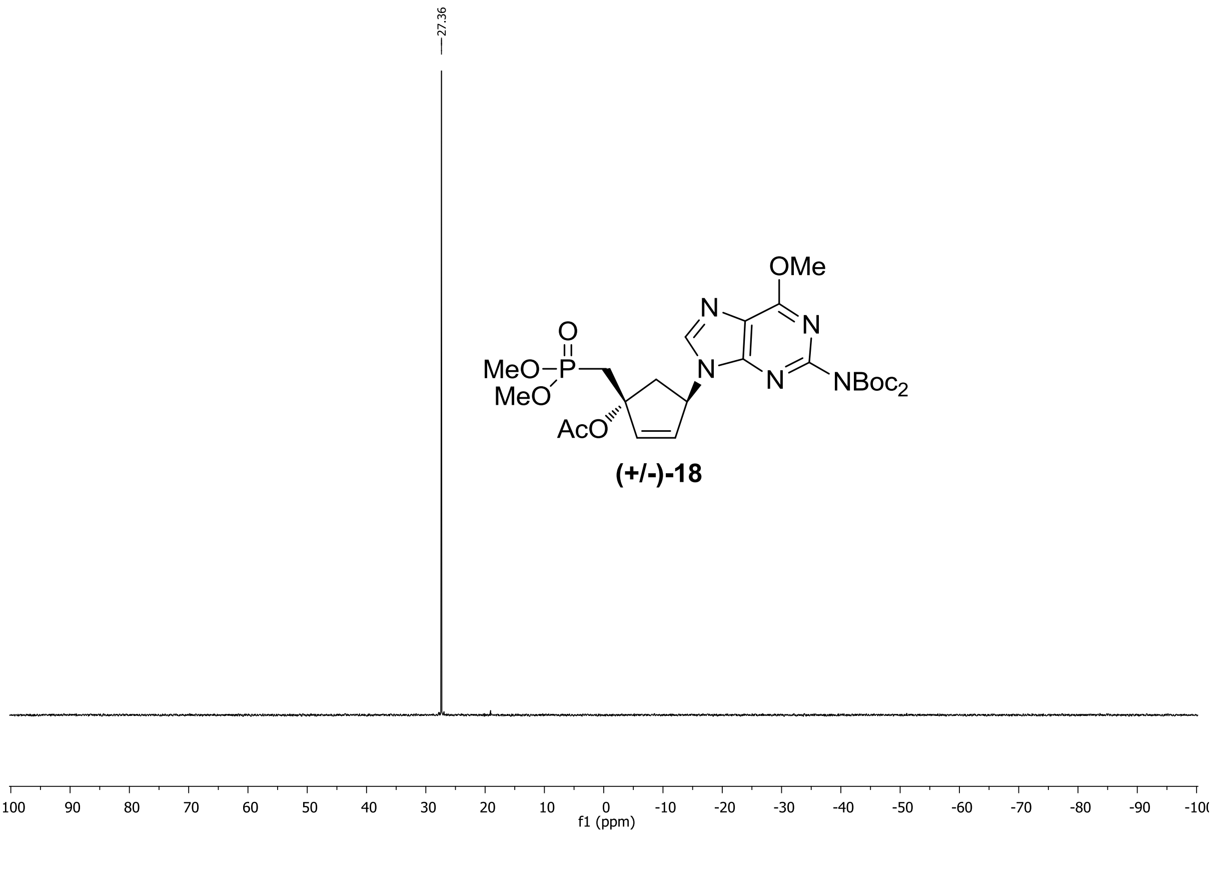
27.36
OMe
N
N
O
MeO
P
MeO
N
N
NBoc2
AcO
100
90
80
70
60
50
40
30
20
10
0
-10
-20
-30
-40
-50
-60
-70
-80
-90
f1 (ppm)

**(+/-)-18**

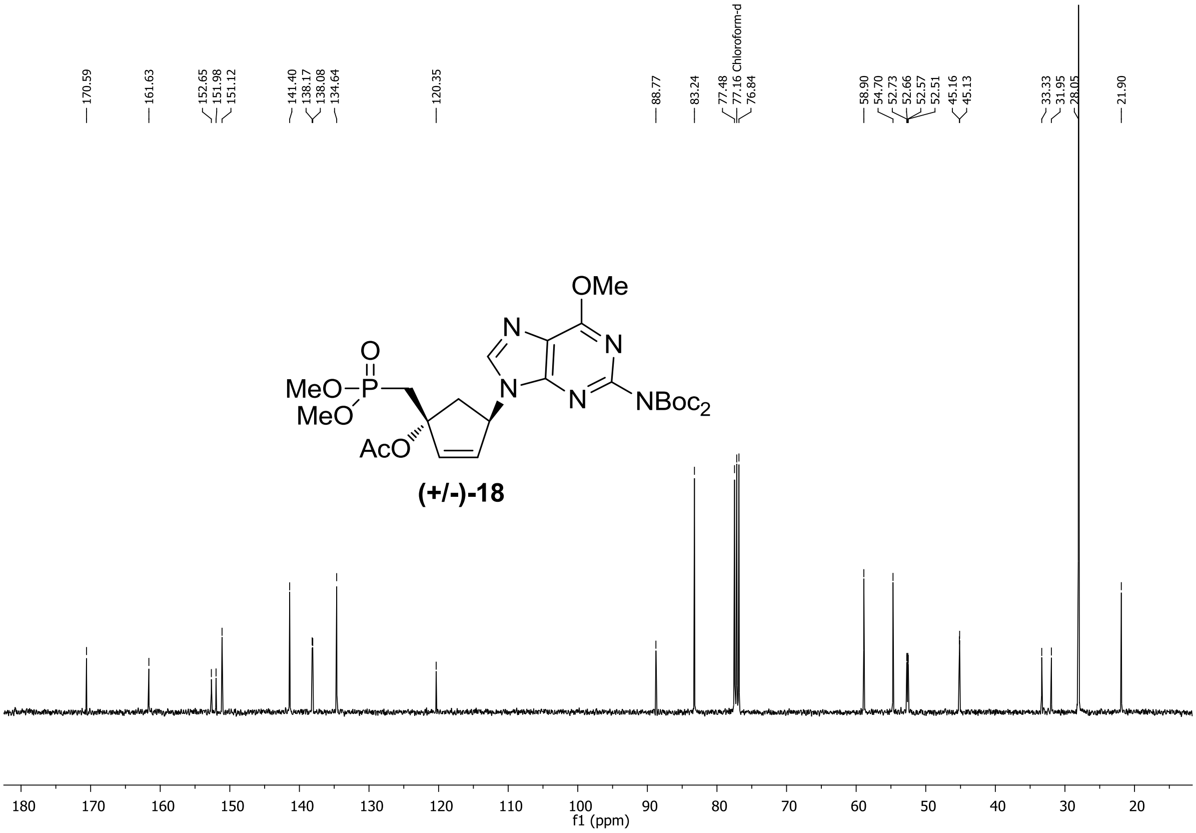
170.59
161.63
152.65
151.98
151.12
141.40
138.17
138.08
134.64
120.35
88.77
83.24
77.48
77.16 Chloroform-d
76.84
58.90
54.70
52.73
52.66
52.57
52.51
45.16
45.13
33.33
31.95
28.05
21.90
OMe
N
N
MeO–P
O
MeO
N
N
NBoc2
AcO
(+/-)-18
180 170 160 150 140 130 120 110 100 90 80 70 60 50 40 30 20
f1 (ppm)

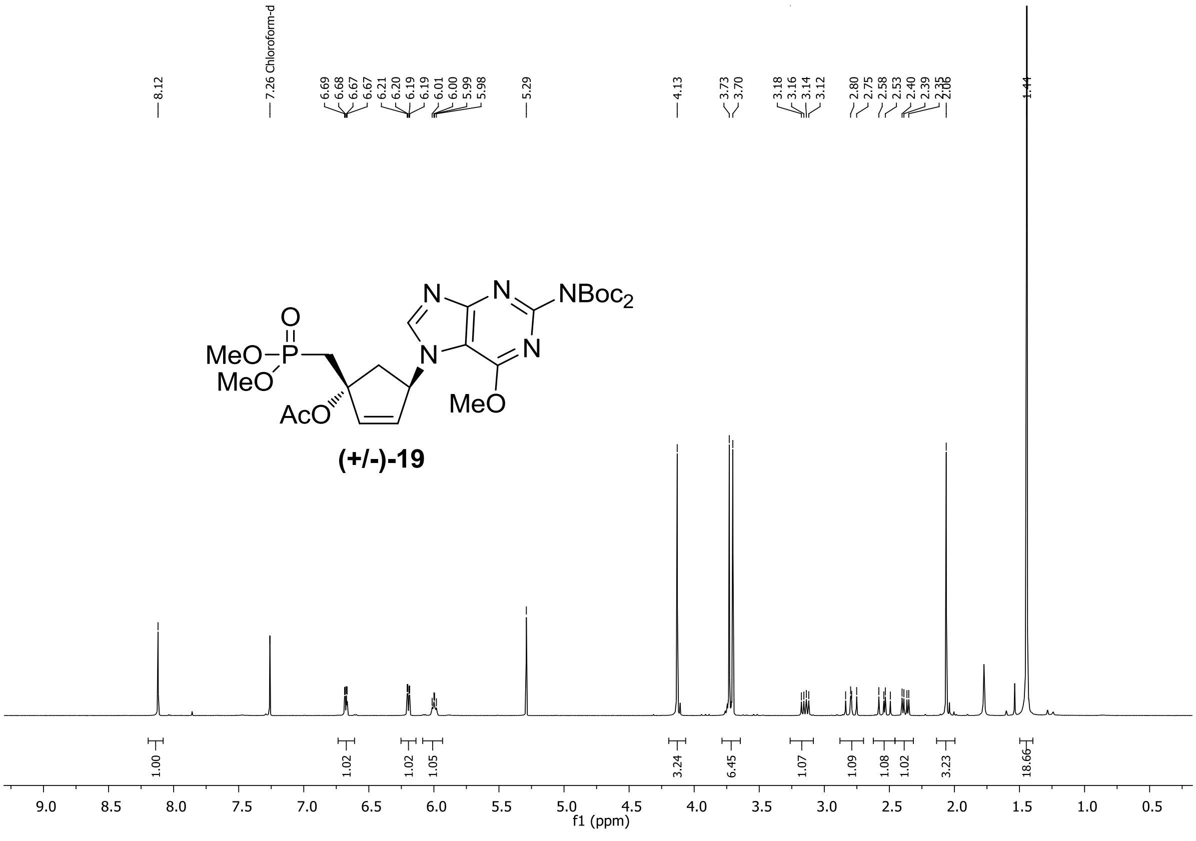
8.12
7.26 Chloroform-d
6.69
6.68
6.67
6.67
6.21
6.20
6.19
6.19
6.01
6.00
5.99
5.98
5.29
4.13
3.73
3.70
3.18
3.16
3.14
3.12
2.80
2.75
2.58
2.53
2.40
2.39
2.35
2.06
1.44
N
N
NBoc2
O
MeO–P
MeO
N
N
AcO
MeO
(+/-)-19
1.00
1.02
1.02
1.05
3.24
6.45
1.07
1.09
1.08
1.02
3.23
18.66
9.0 8.5 8.0 7.5 7.0 6.5 6.0 5.5 5.0 4.5 4.0 3.5 3.0 2.5 2.0 1.5 1.0 0.5
f1 (ppm)

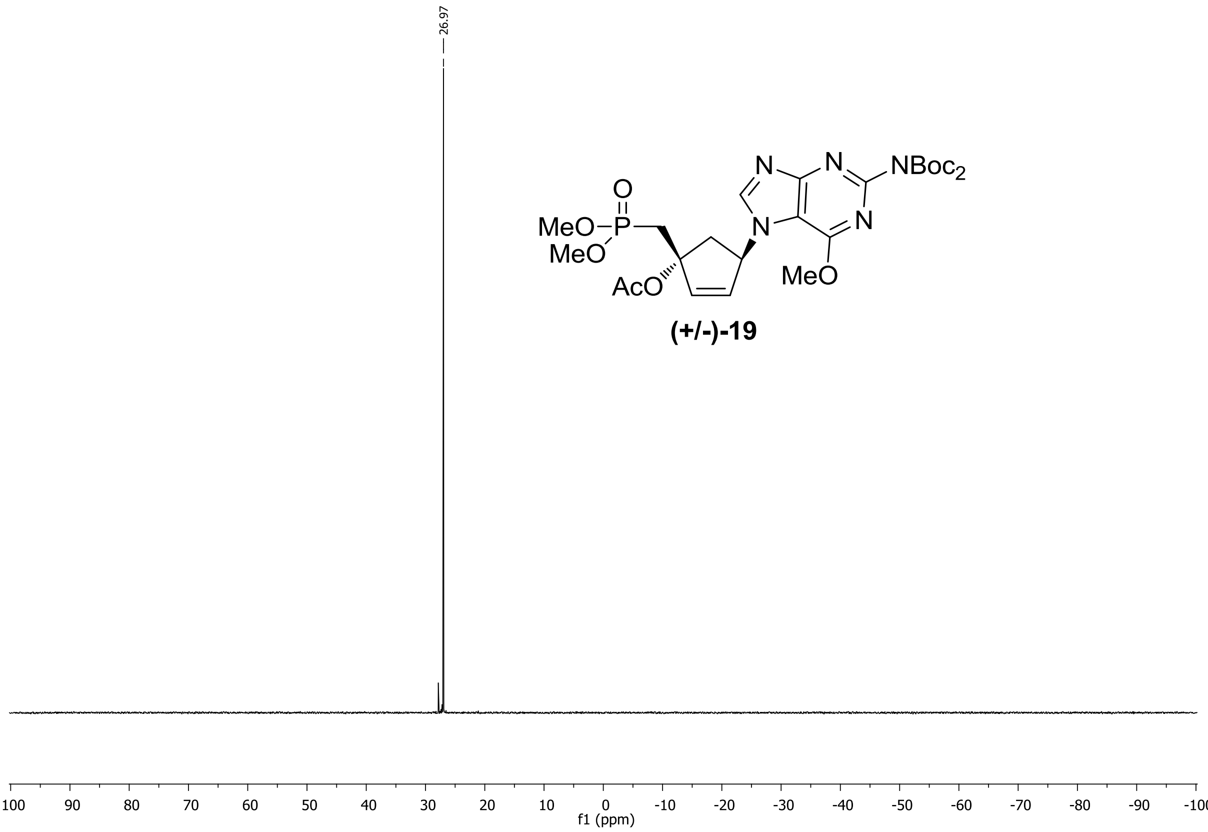

26.97
MeO
MeO
P
O
AcO
N
N
N
N
N
NBoc2
MeO
(+/-)-19
100 90 80 70 60 50 40 30 20 10 0 -10 -20 -30 -40 -50 -60 -70 -80 -90 -100
f1 (ppm)


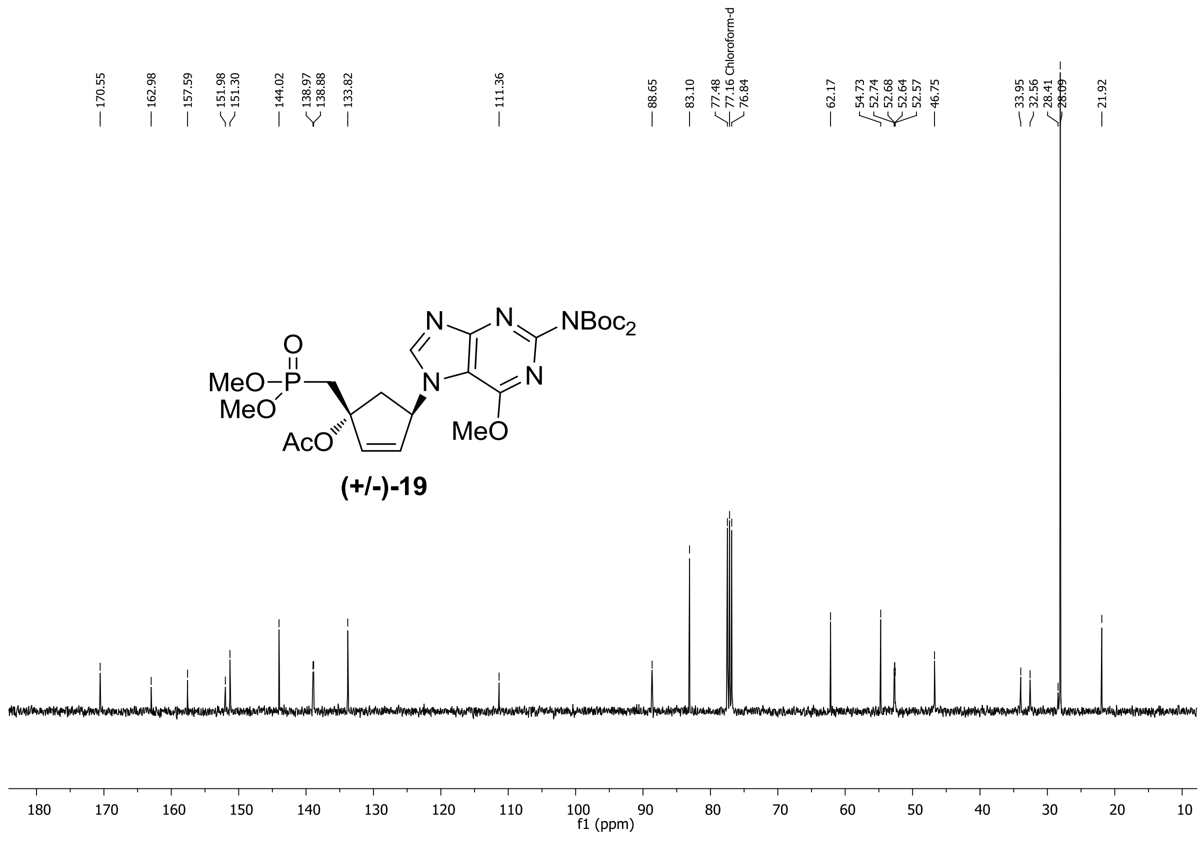

170.55
162.98
157.59
151.98
151.30
144.02
138.97
138.88
133.82
111.36
88.65
83.10
77.48
77.16 Chloroform-d
76.84
62.17
54.73
52.74
52.68
52.64
52.57
46.75
33.95
32.56
28.41
28.09
21.92
MeO
MeO
P
O
AcO
N
N
N
N
N
NBoc2
MeO
(+/-)-19
180 170 160 150 140 130 120 110 100 90 80 70 60 50 40 30 20 10
f1 (ppm)

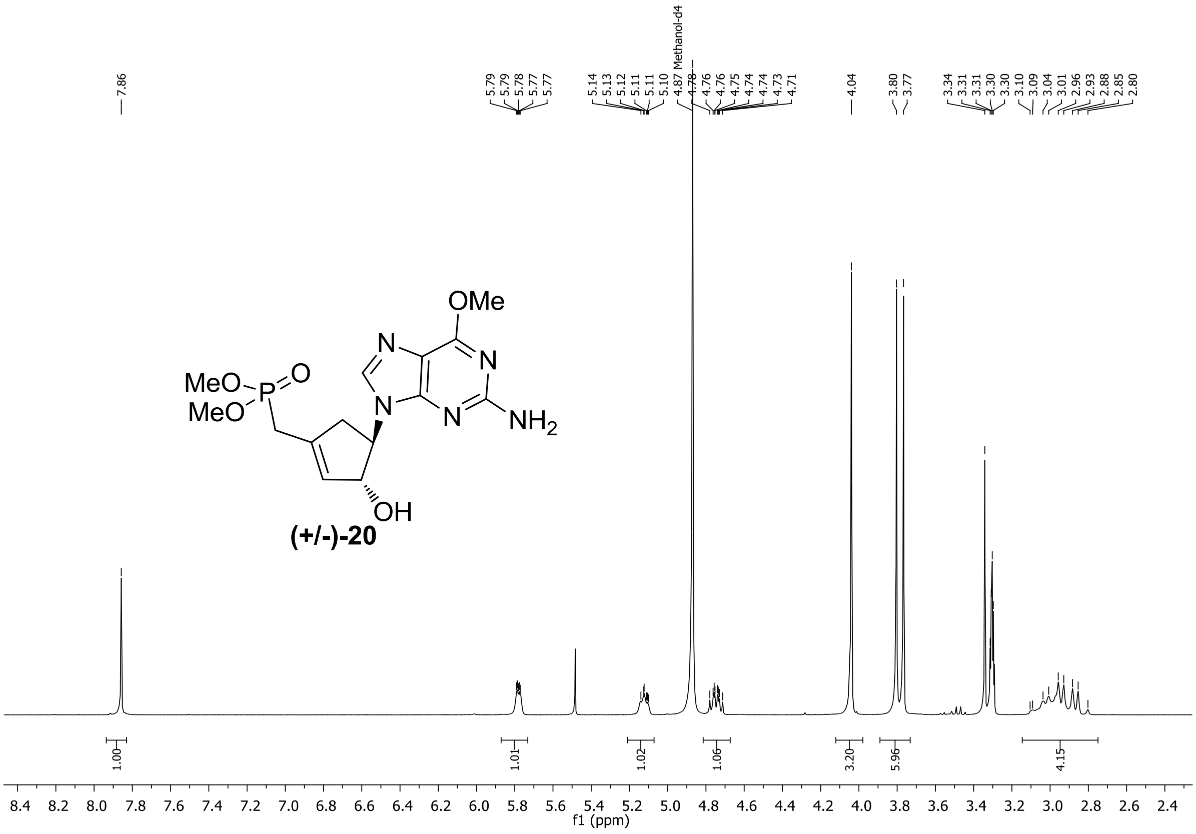
7.86
5.79 5.79 5.78 5.77 5.77
5.14 5.13 5.12 5.11 5.11 5.10
4.87 Methanol-d4
4.78 4.76 4.76 4.75 4.74 4.74 4.73 4.71
4.04
3.80 3.77
3.34 3.31 3.31 3.30 3.30 3.10 3.09 3.04 3.01 2.96 2.93 2.88 2.85 2.80
OMe
N
MeO
P
O
MeO
NH2
OH
(+/-)-20
1.00
1.01
1.02
1.06
3.20
5.96
4.15
f1 (ppm)

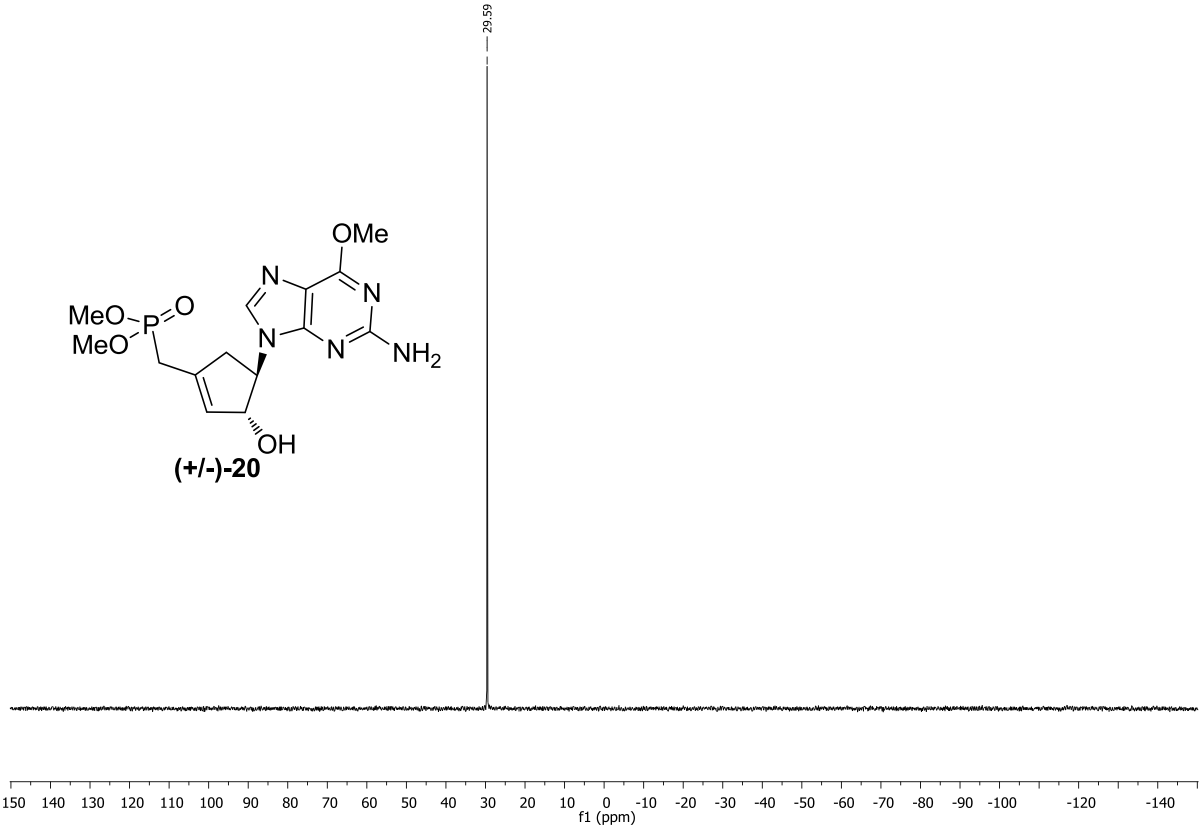
29.59
OMe
N
MeO
P
O
MeO
NH2
OH
(+/-)-20
f1 (ppm)

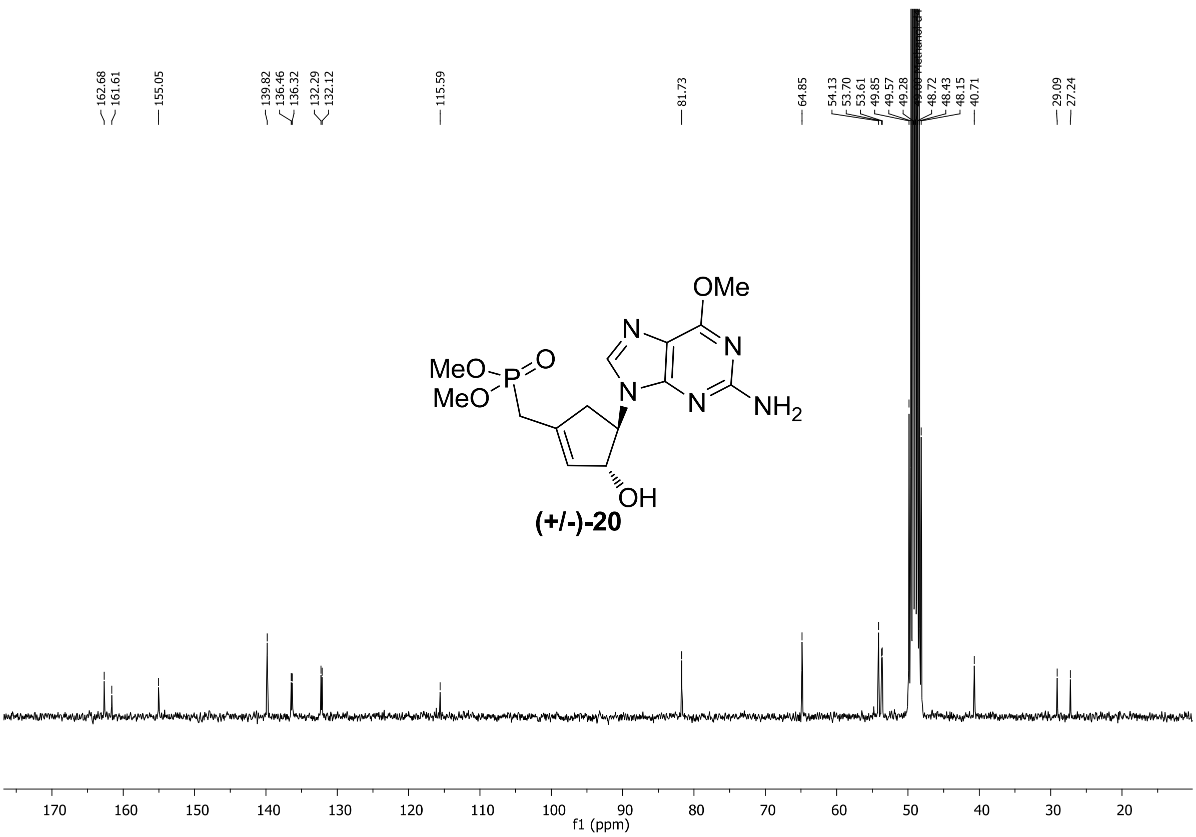
162.68
161.61
155.05
139.82
136.46
136.32
132.29
132.12
115.59
81.73
64.85
54.13
53.70
53.61
49.85
49.57
49.28
48.72
48.43
48.15
40.71
29.09
27.24
OMe
N
MeO
MeO
P
O
NH2
OH
(+/-)-20
f1 (ppm)

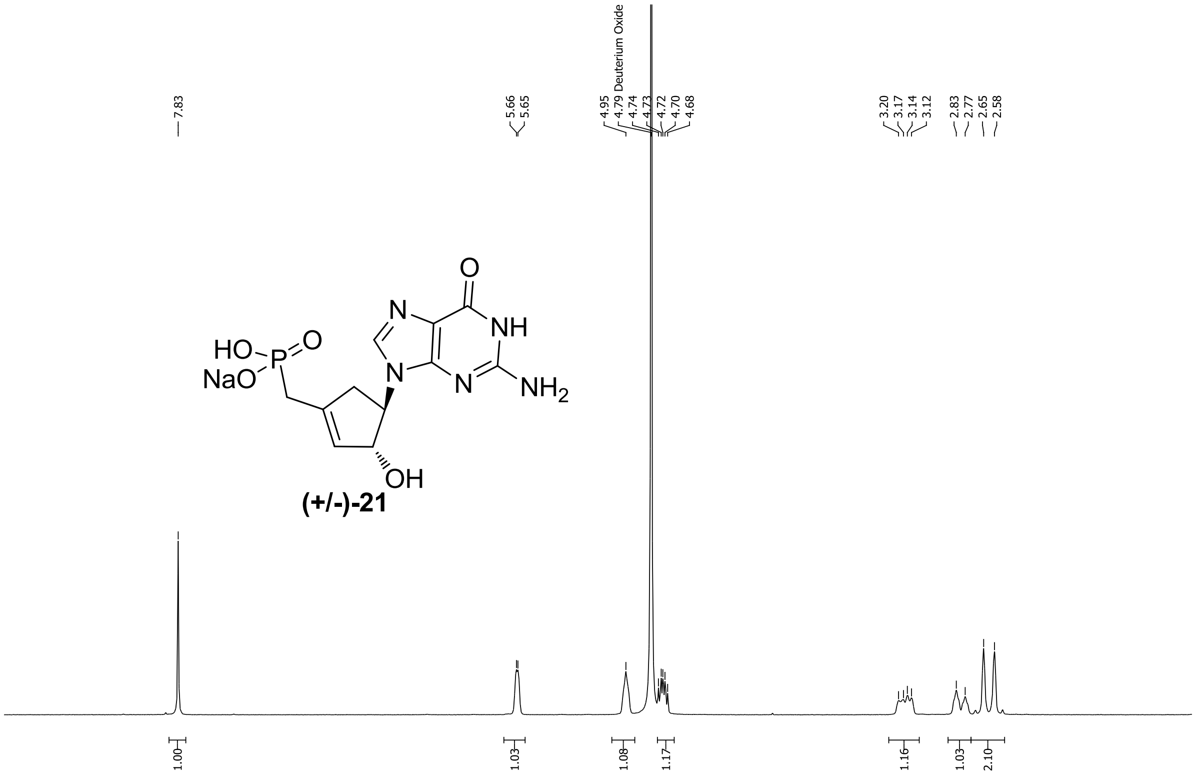
7.83
5.66
5.65
4.95
4.79 Deuterium Oxide
4.74
4.73
4.72
4.70
4.68
3.20
3.17
3.14
3.12
2.83
2.77
2.65
2.58
O
NH
N
HO
NaO
P
NH2
OH
(+/-)-21
1.00
1.03
1.08
1.17
1.16
1.03
2.10
f1 (ppm)

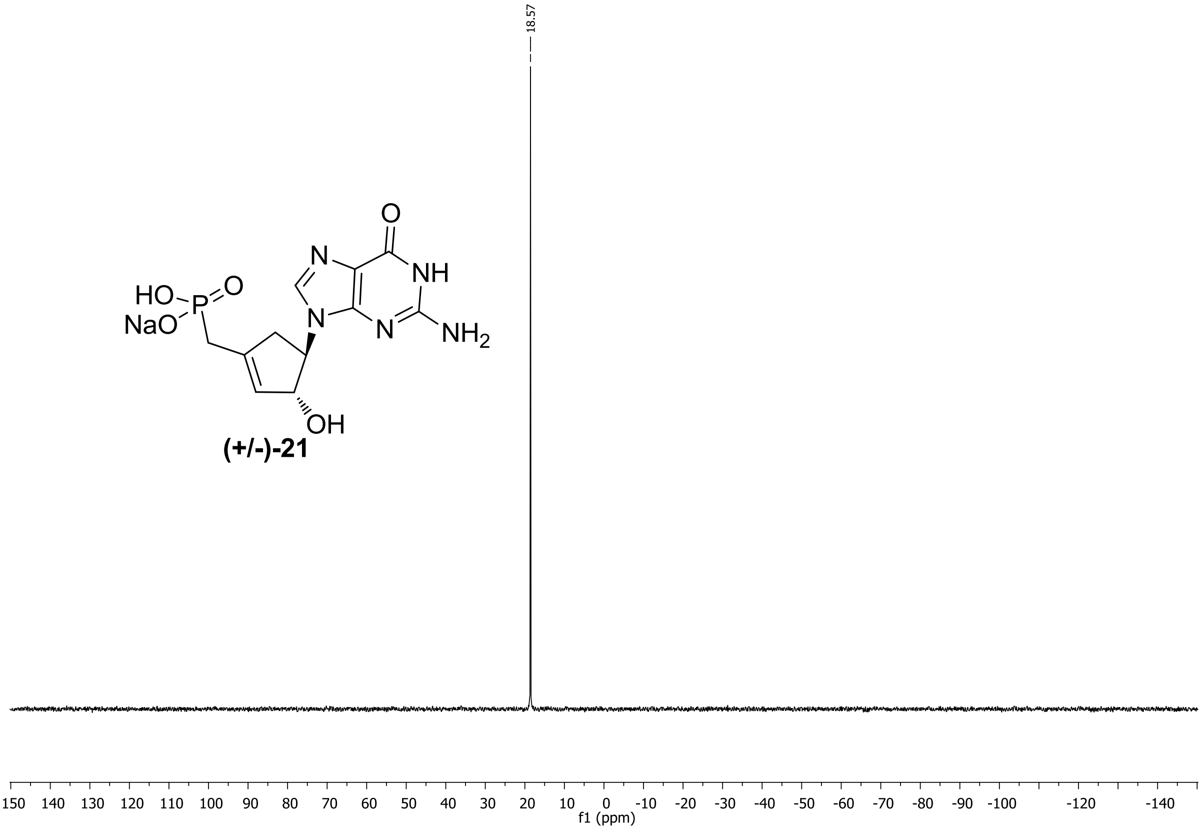
18.57
HO
NaO
P
O
N
NH
NH2
OH
(+/-)-21
150 140 130 120 110 100 90 80 70 60 50 40 30 20 10 0 -10 -20 -30 -40 -50 -60 -70 -80 -90 -100 -120 -140
f1 (ppm)

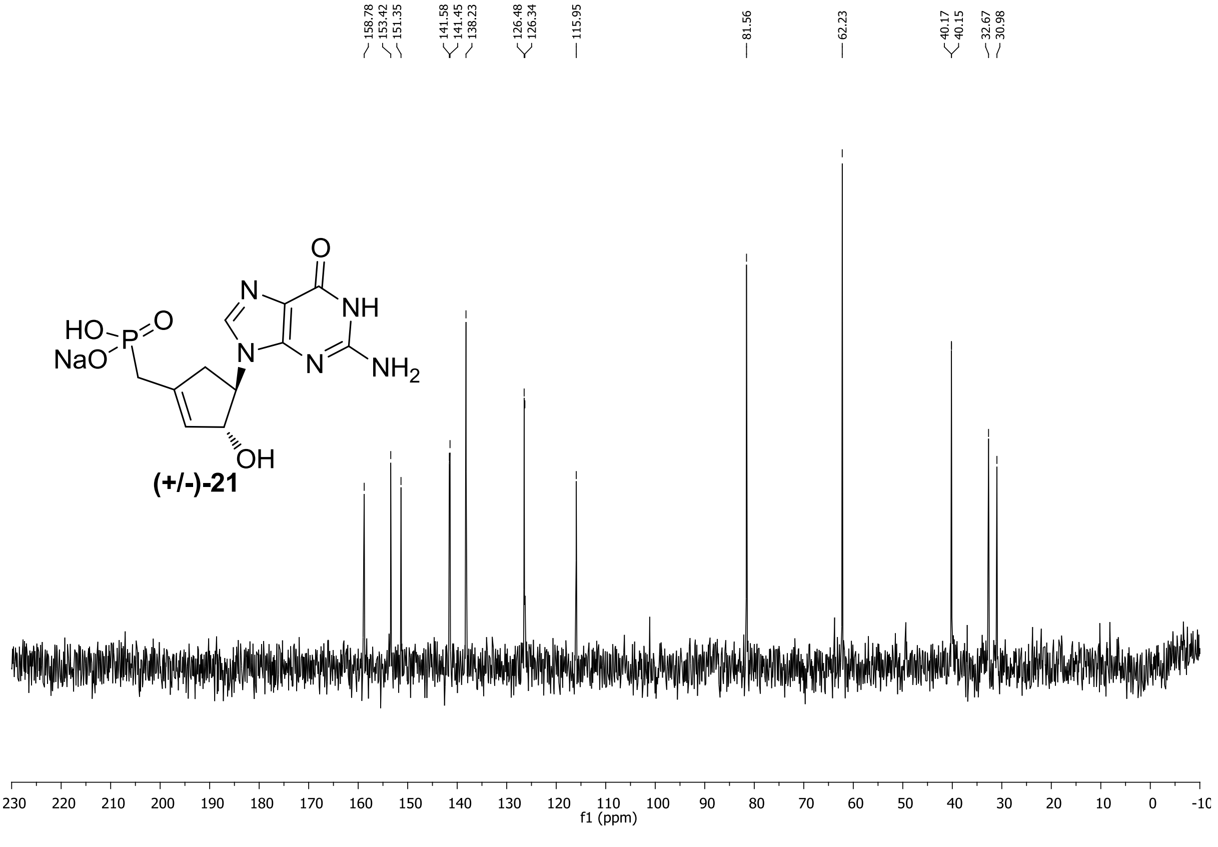
158.78
153.42
151.35
141.58
141.45
138.23
126.48
126.34
115.95
81.56
62.23
40.17
40.15
32.67
30.98
HO
NaO
P
O
N
NH
NH2
OH
(+/-)-21
230 220 210 200 190 180 170 160 150 140 130 120 110 100 90 80 70 60 50 40 30 20 10 0 -10
f1 (ppm)

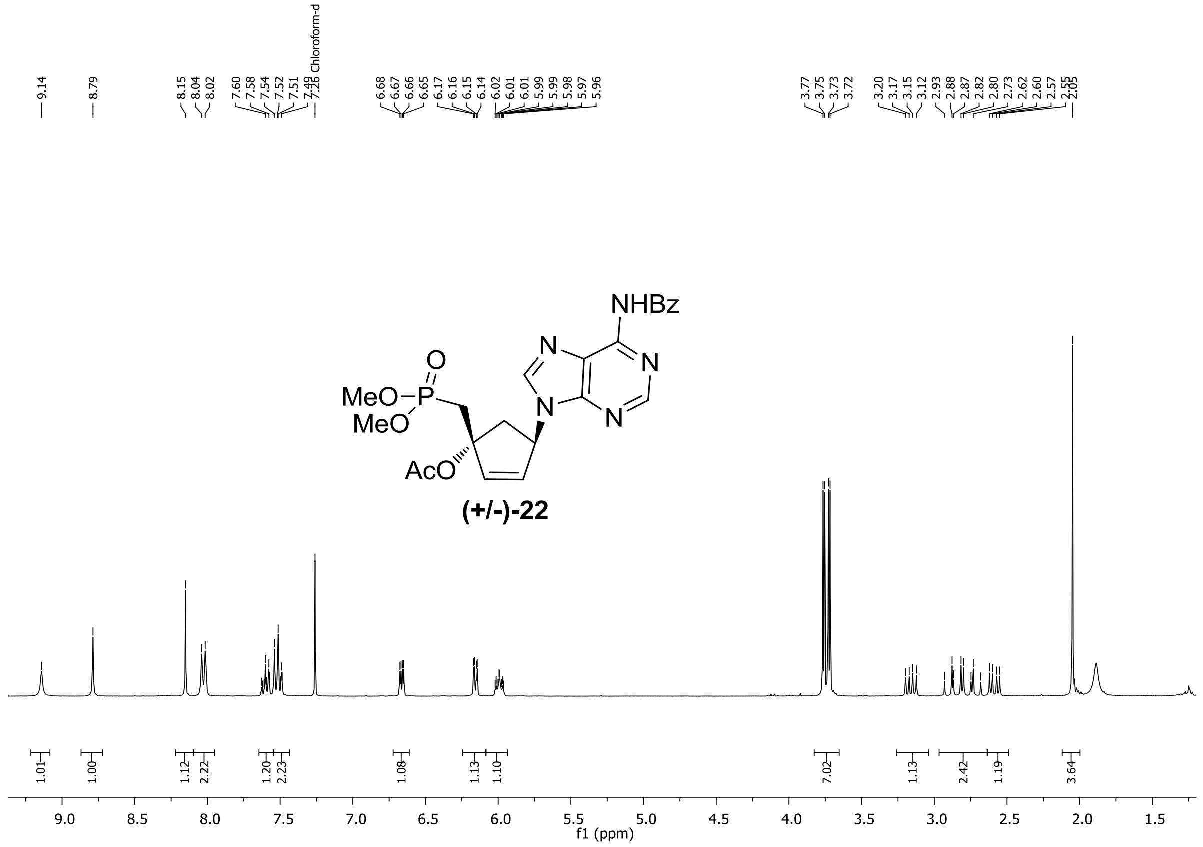
NHBz
MeO
MeO
AcO
(+/-)-22
Chloroform-d
f1 (ppm)

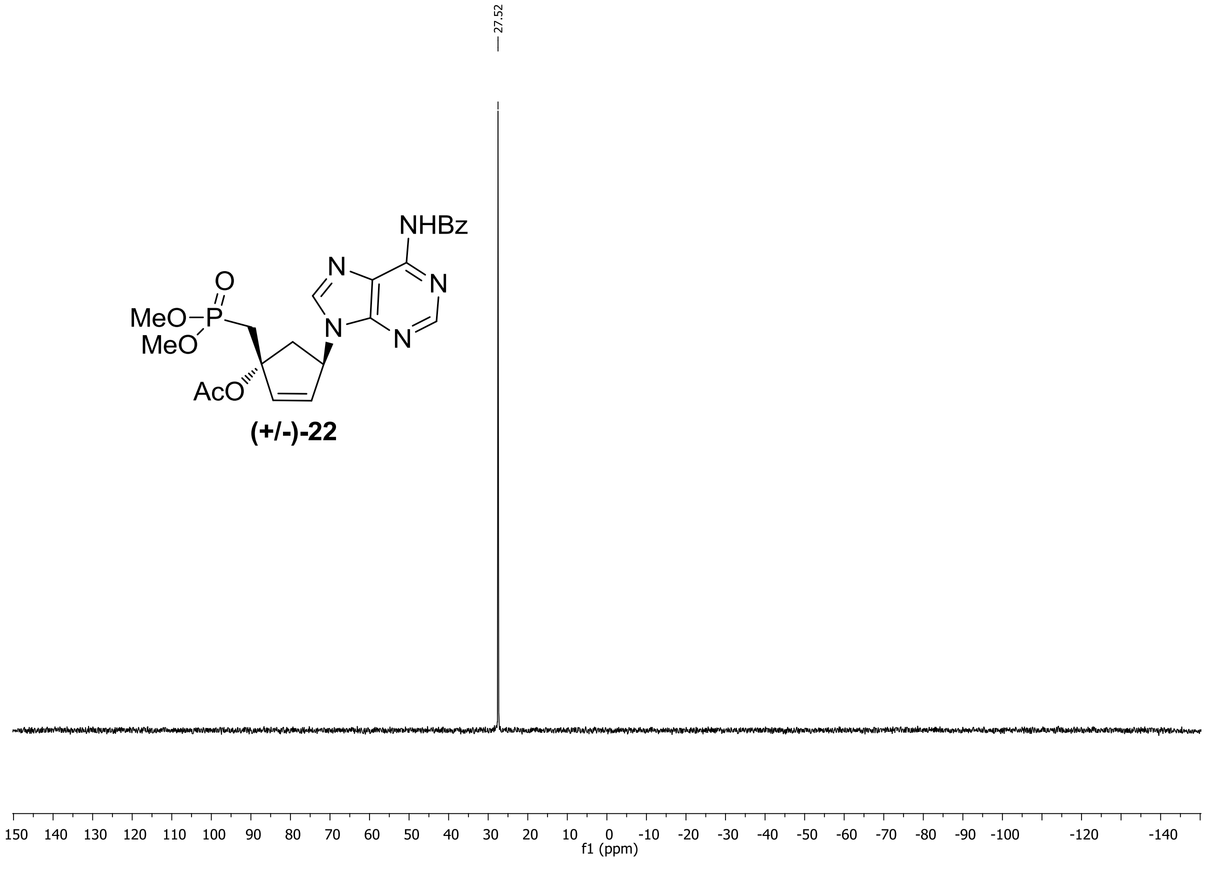
27.52
NHBz
MeO
MeO
AcO
(+/-)-22
f1 (ppm)

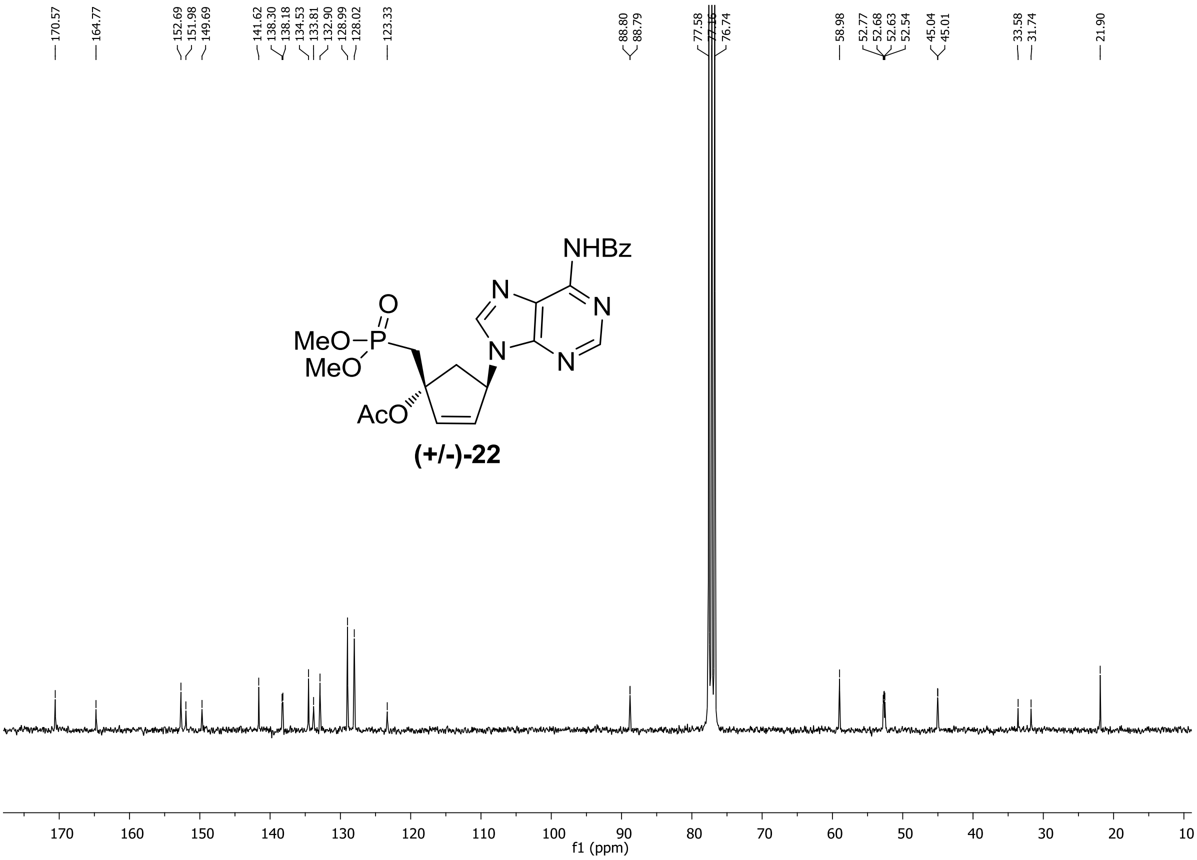
170.57
164.77
152.69
151.98
149.69
141.62
138.30
138.18
134.53
133.81
132.90
128.99
128.02
123.33
88.80
88.79
77.58
76.74
58.98
52.77
52.68
52.63
52.54
45.04
45.01
33.58
31.74
21.90
NHBz
N
N
N
N
O
MeO
P
MeO
AcO
(+/-)-22
170
160
150
140
130
120
110
100
90
80
70
60
50
40
30
20
10
f1 (ppm)

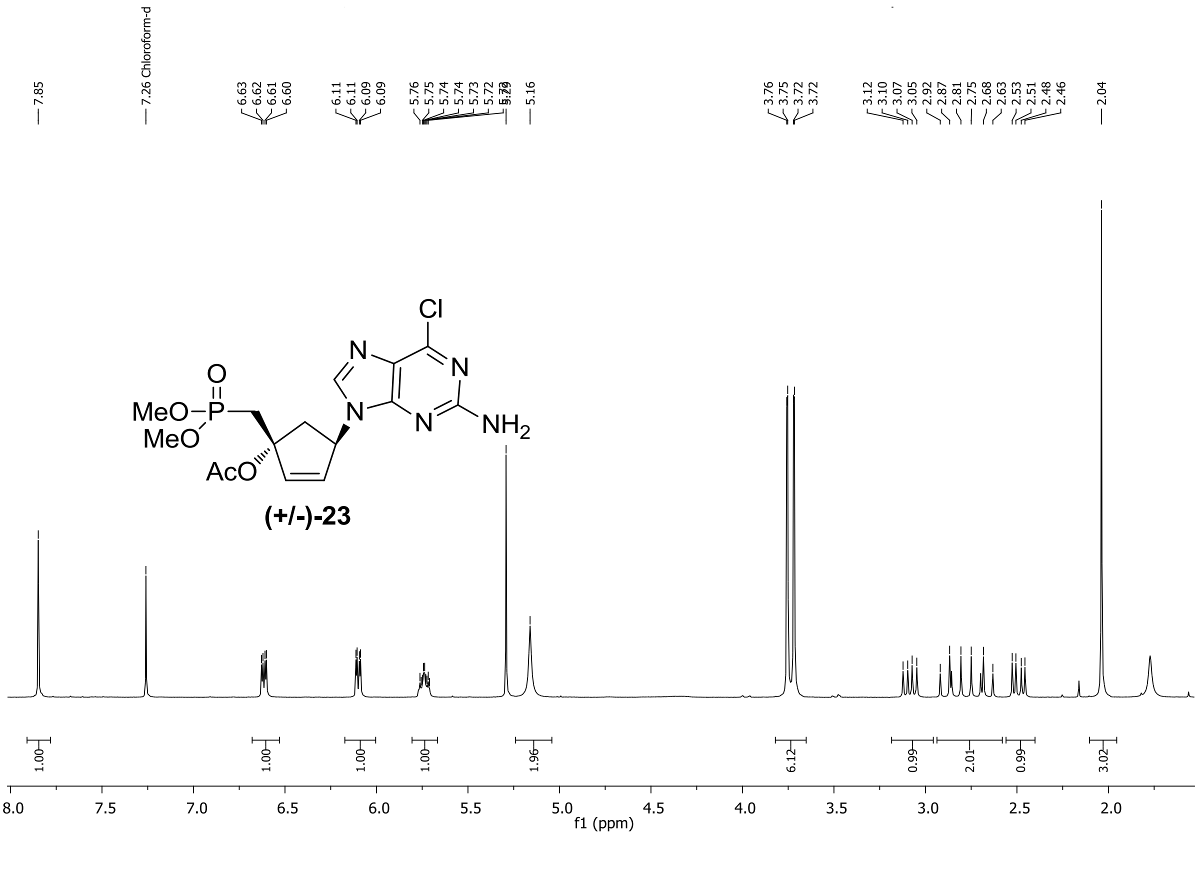
7.85
7.26 Chloroform-d
6.63
6.62
6.61
6.60
6.11
6.11
6.09
6.09
5.76
5.75
5.74
5.74
5.73
5.72
5.16
3.76
3.75
3.72
3.72
3.12
3.10
3.07
3.05
2.92
2.87
2.81
2.75
2.68
2.63
2.53
2.51
2.48
2.46
2.04
Cl
N
N
N
N
NH2
O
MeO
P
MeO
AcO
(+/-)-23
1.00
1.00
1.00
1.00
1.96
6.12
0.99
2.01
0.99
3.02
8.0
7.5
7.0
6.5
6.0
5.5
5.0
4.5
4.0
3.5
3.0
2.5
2.0
f1 (ppm)

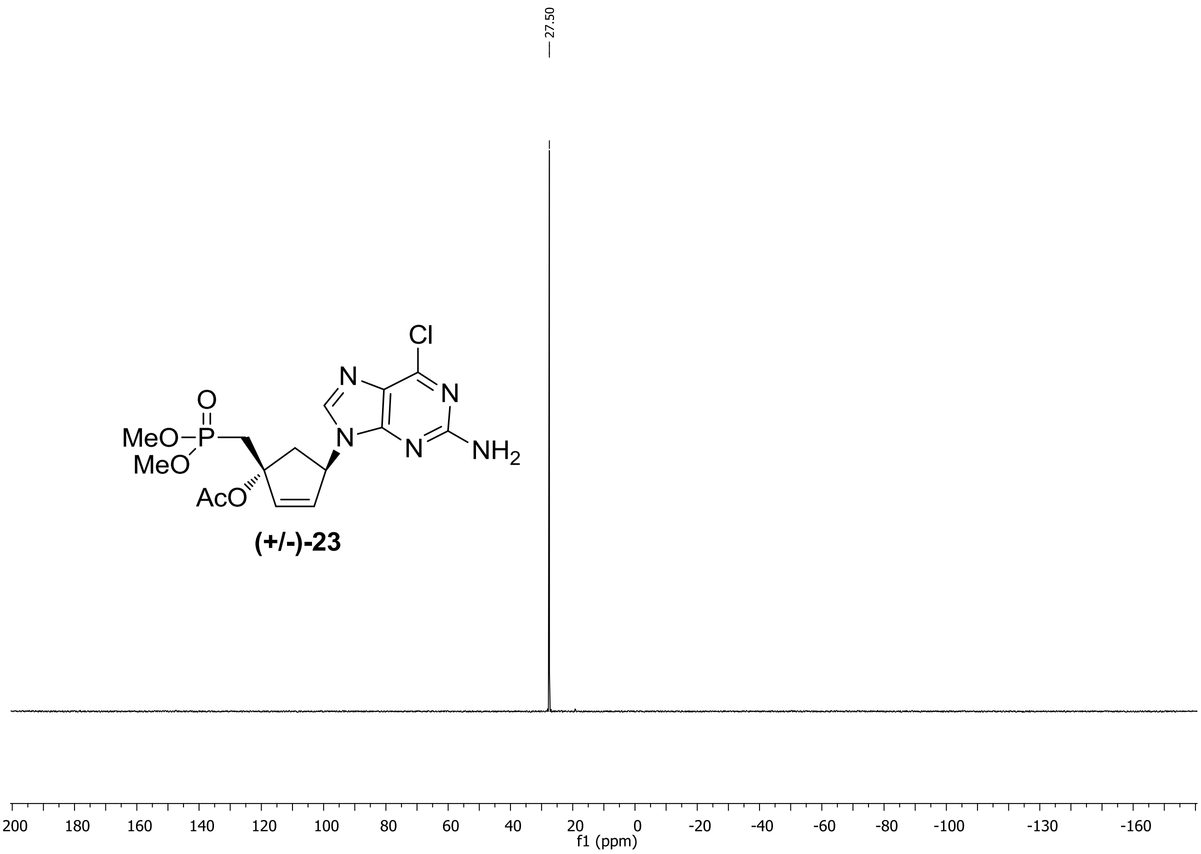
27.50
Cl
N
N
O
MeO–P
MeO
N
N
NH2
AcO
(+/-)-23
200 180 160 140 120 100 80 60 40 20 0 -20 -40 -60 -80 -100 -130 -160
f1 (ppm)

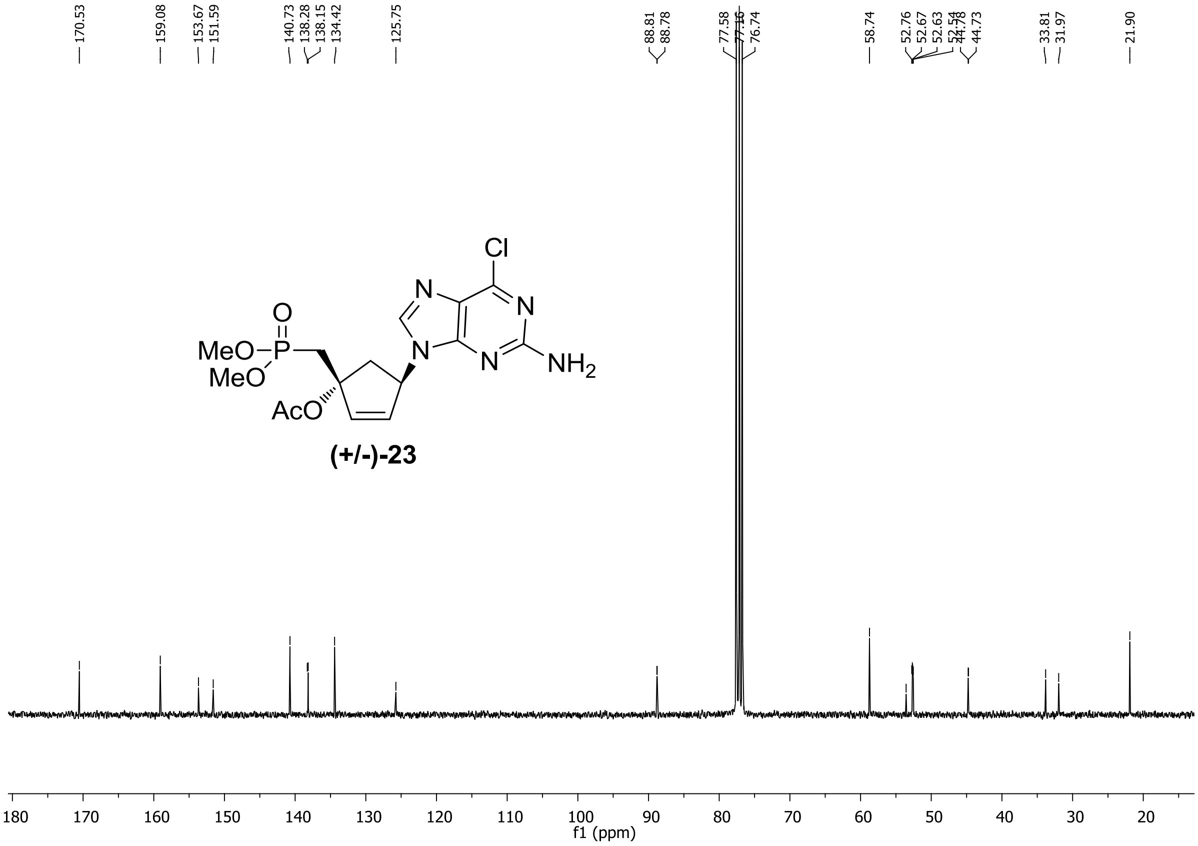
170.53
159.08
153.67
151.59
140.73
138.28
138.15
134.42
125.75
88.81
88.78
77.58
77.16
76.74
58.74
52.76
52.67
52.63
52.54
44.78
44.73
33.81
31.97
21.90
Cl
N
N
O
MeO–P
MeO
N
N
NH2
AcO
(+/-)-23
180 170 160 150 140 130 120 110 100 90 80 70 60 50 40 30 20
f1 (ppm)

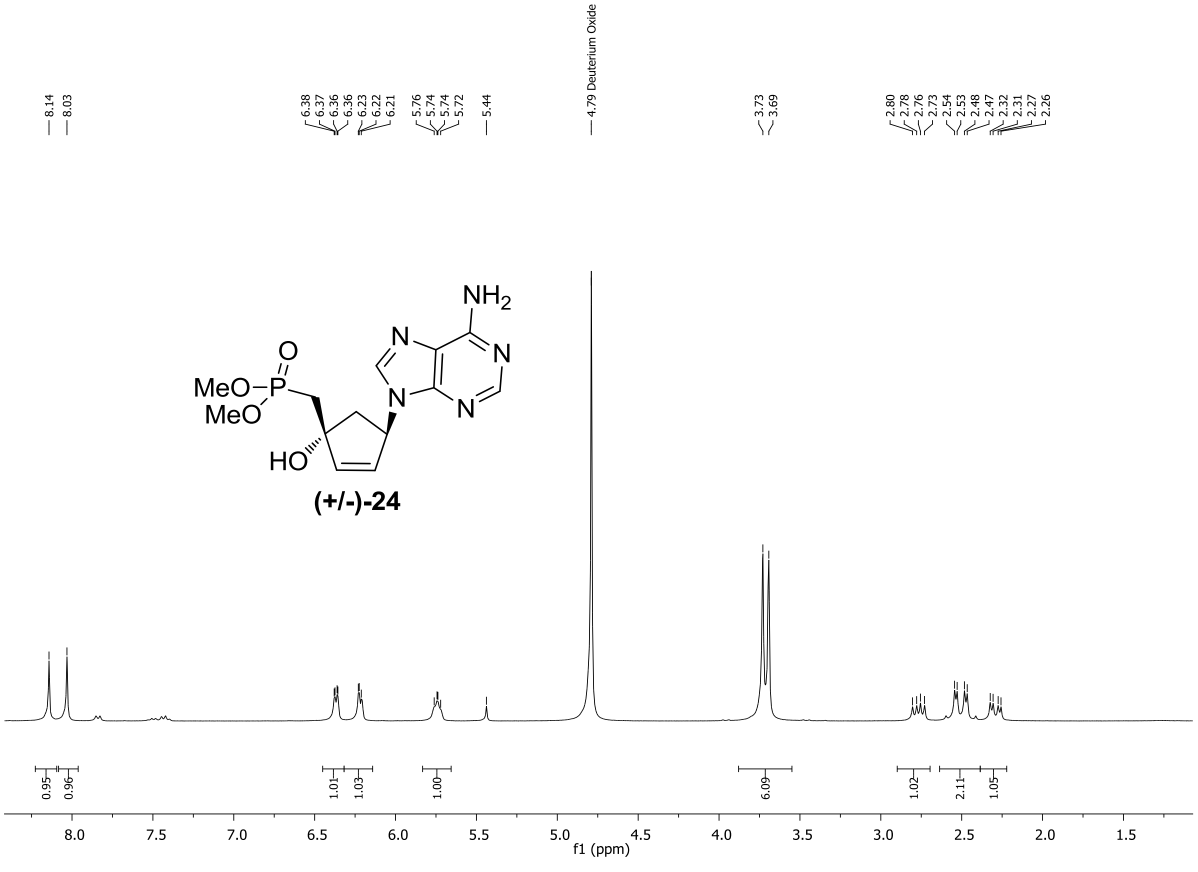
8.14
8.03
6.38
6.37
6.36
6.36
6.23
6.22
6.21
5.76
5.74
5.74
5.72
5.44
4.79 Deuterium Oxide
3.73
3.69
2.80
2.78
2.76
2.73
2.54
2.53
2.48
2.47
2.32
2.31
2.27
2.26
NH2
N
N
N
N
O
MeO–P
MeO
HO
(+/-)-24
0.95
0.96
1.01
1.03
1.00
6.09
1.02
2.11
1.05
8.0 7.5 7.0 6.5 6.0 5.5 5.0 4.5 4.0 3.5 3.0 2.5 2.0 1.5
f1 (ppm)

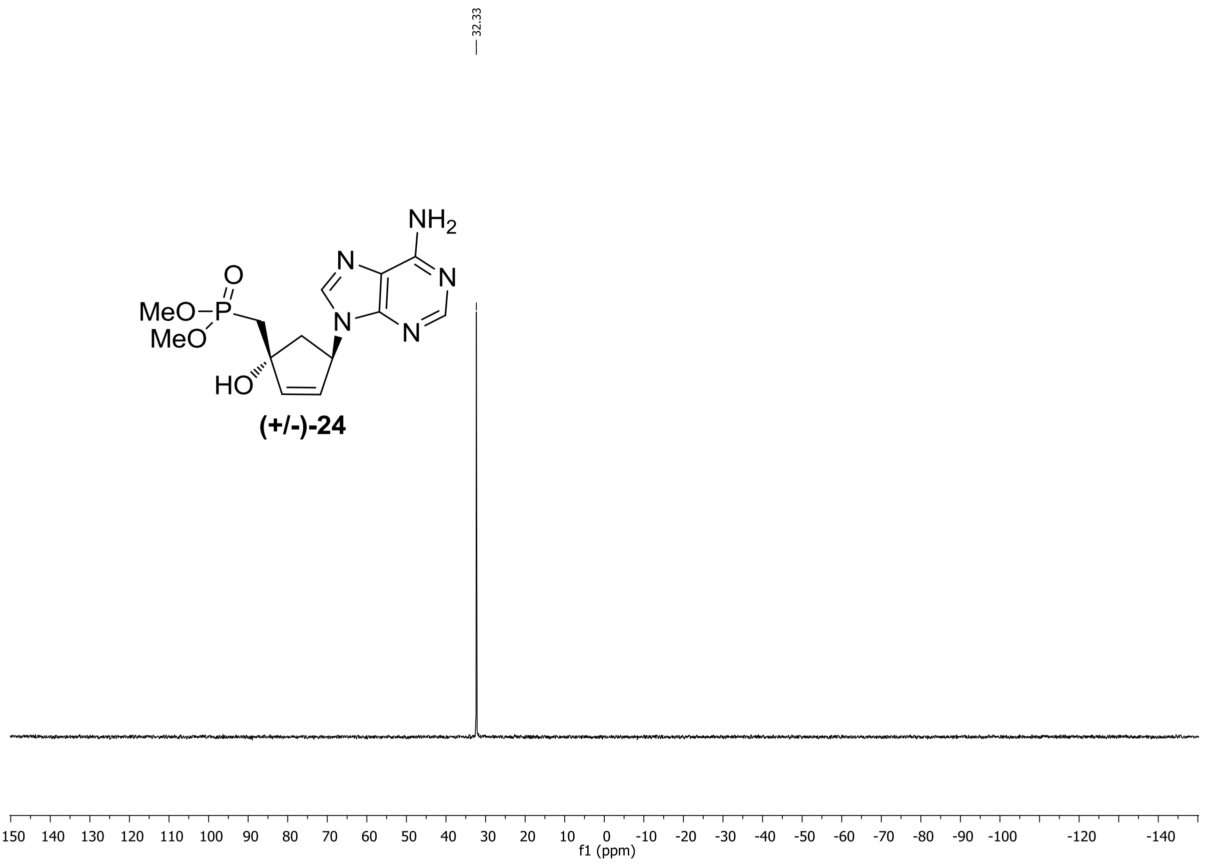
32.33
NH2
N
N
N
N
O
MeO–P
MeO
HO
(+/-)-24
150 140 130 120 110 100 90 80 70 60 50 40 30 20 10 0 -10 -20 -30 -40 -50 -60 -70 -80 -90 -100 -120 -140
f1 (ppm)

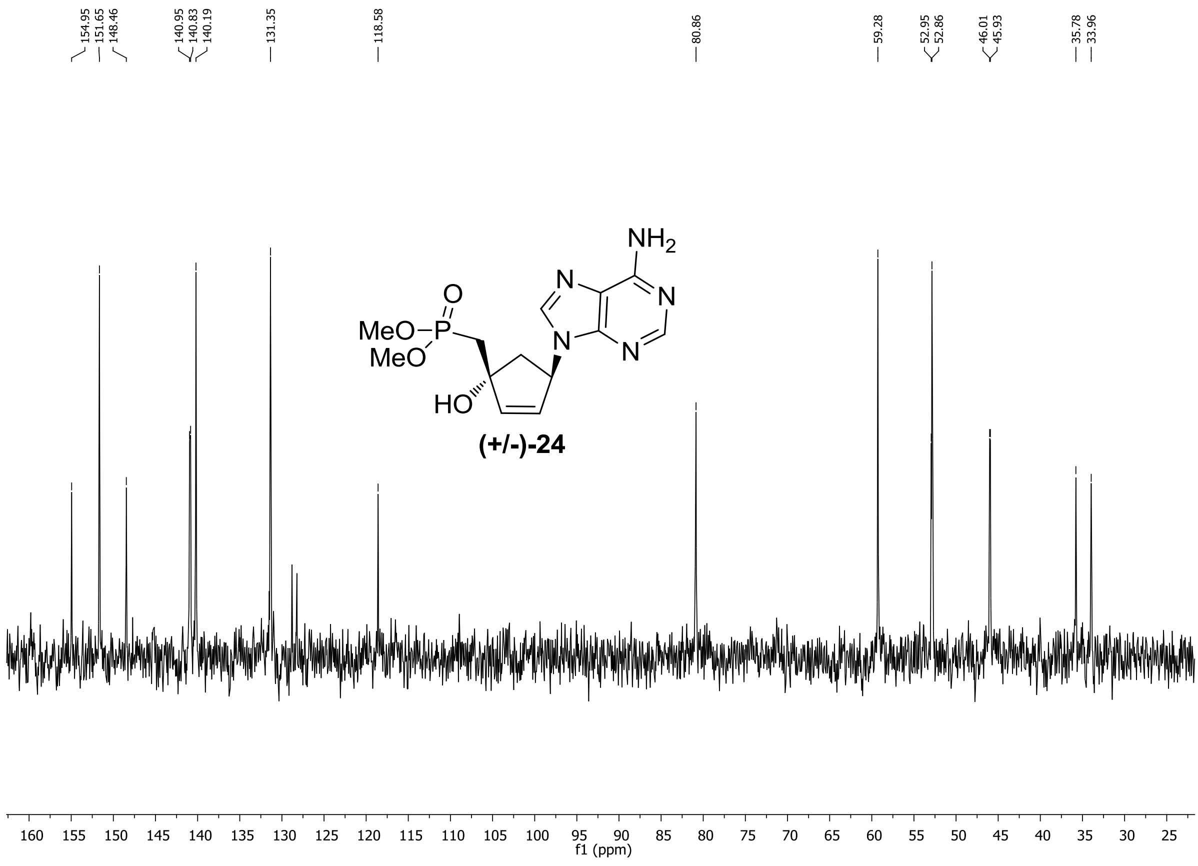
154.95
151.65
148.46
140.95
140.83
140.19
131.35
118.58
80.86
59.28
52.95
52.86
46.01
45.93
35.78
33.96
NH2
N
N
O
MeO–P
MeO
N
N
HO
(+/-)-24
160 155 150 145 140 135 130 125 120 115 110 105 100 95 90 85 80 75 70 65 60 55 50 45 40 35 30 25
f1 (ppm)

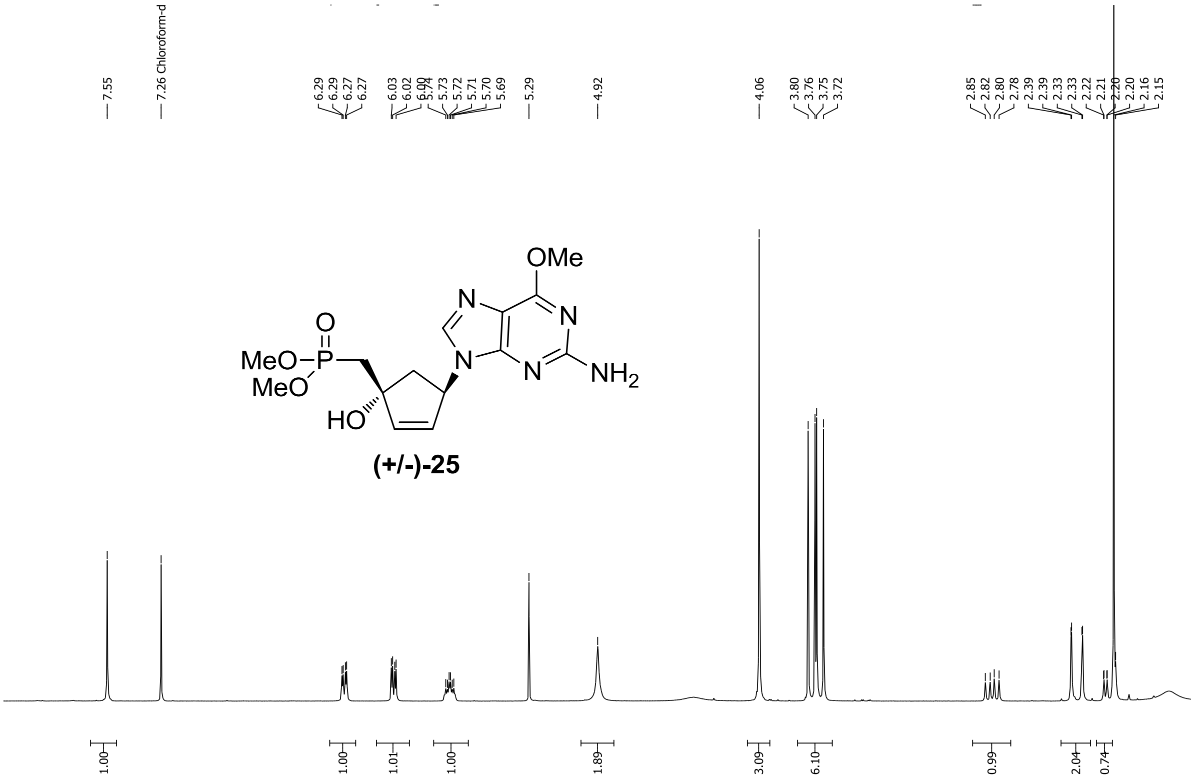
7.55
7.26 Chloroform-d
6.29
6.29
6.27
6.27
6.03
6.02
6.00
5.74
5.73
5.72
5.71
5.70
5.69
5.29
4.92
4.06
3.80
3.76
3.75
3.72
2.85
2.82
2.80
2.78
2.39
2.39
2.33
2.33
2.22
2.21
2.20
2.20
2.16
2.15
OMe
N
N
O
MeO–P
MeO
N
N
NH2
HO
(+/-)-25
1.00
1.00
1.01
1.00
1.89
3.09
6.10
0.99
2.04
0.74
8.0 7.8 7.6 7.4 7.2 7.0 6.8 6.6 6.4 6.2 6.0 5.8 5.6 5.4 5.2 5.0 4.8 4.6 4.4 4.2 4.0 3.8 3.6 3.4 3.2 3.0 2.8 2.6 2.4 2.2 2.0 1.8
f1 (ppm)

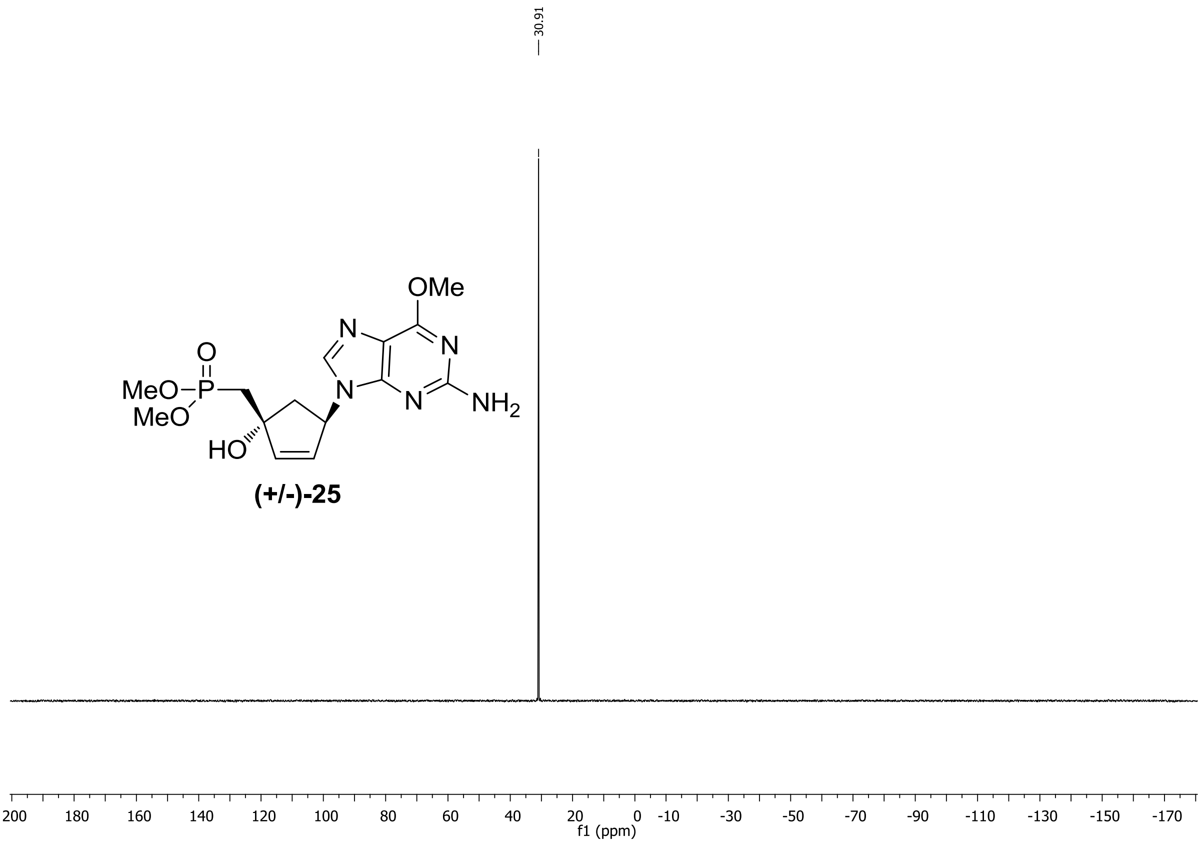
30.91
OMe
N
MeO–P
MeO
HO
NH2
(+/-)-25
200 180 160 140 120 100 80 60 40 20 0 -10 -30 -50 -70 -90 -110 -130 -150 -170
f1 (ppm)

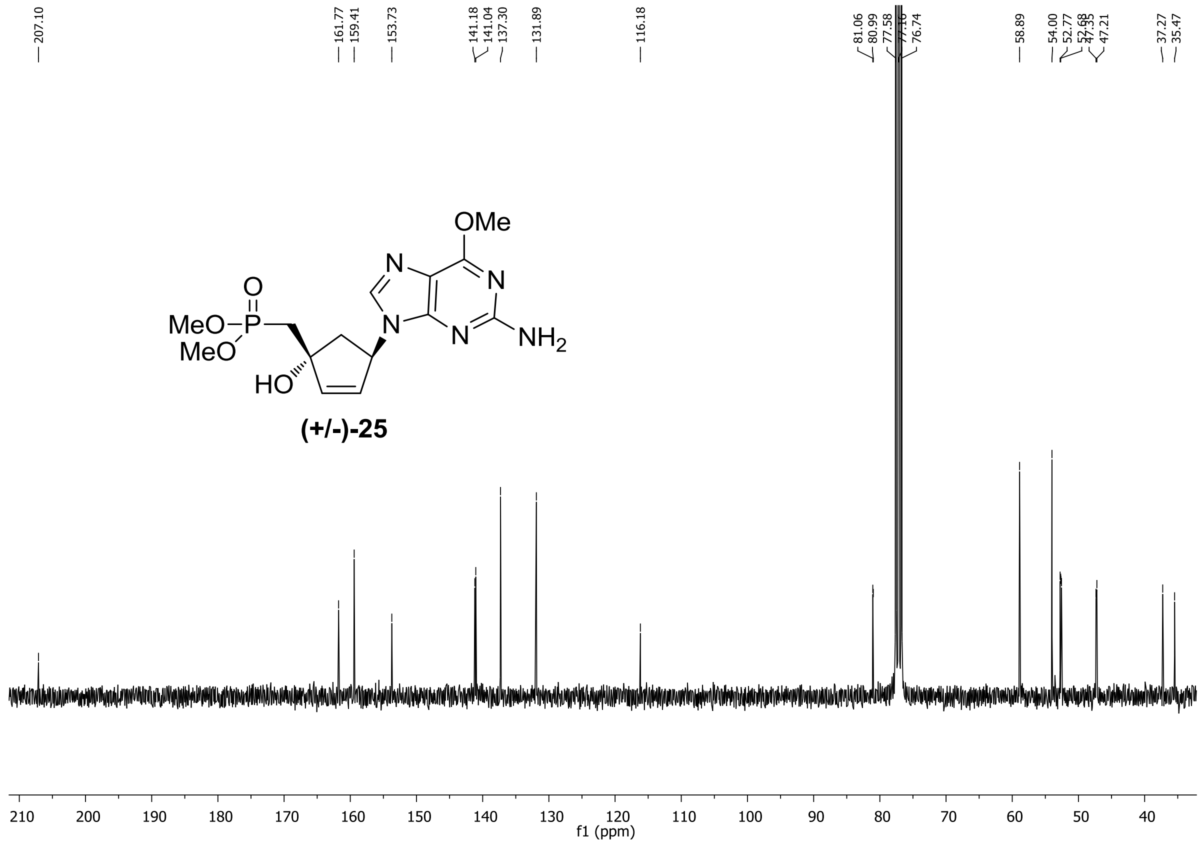
207.10
161.77
159.41
153.73
141.18
141.04
137.30
131.89
116.18
81.06
80.99
77.58
76.74
58.89
54.00
52.77
47.21
37.27
35.47
OMe
N
MeO–P
MeO
HO
NH2
(+/-)-25
210 200 190 180 170 160 150 140 130 120 110 100 90 80 70 60 50 40
f1 (ppm)

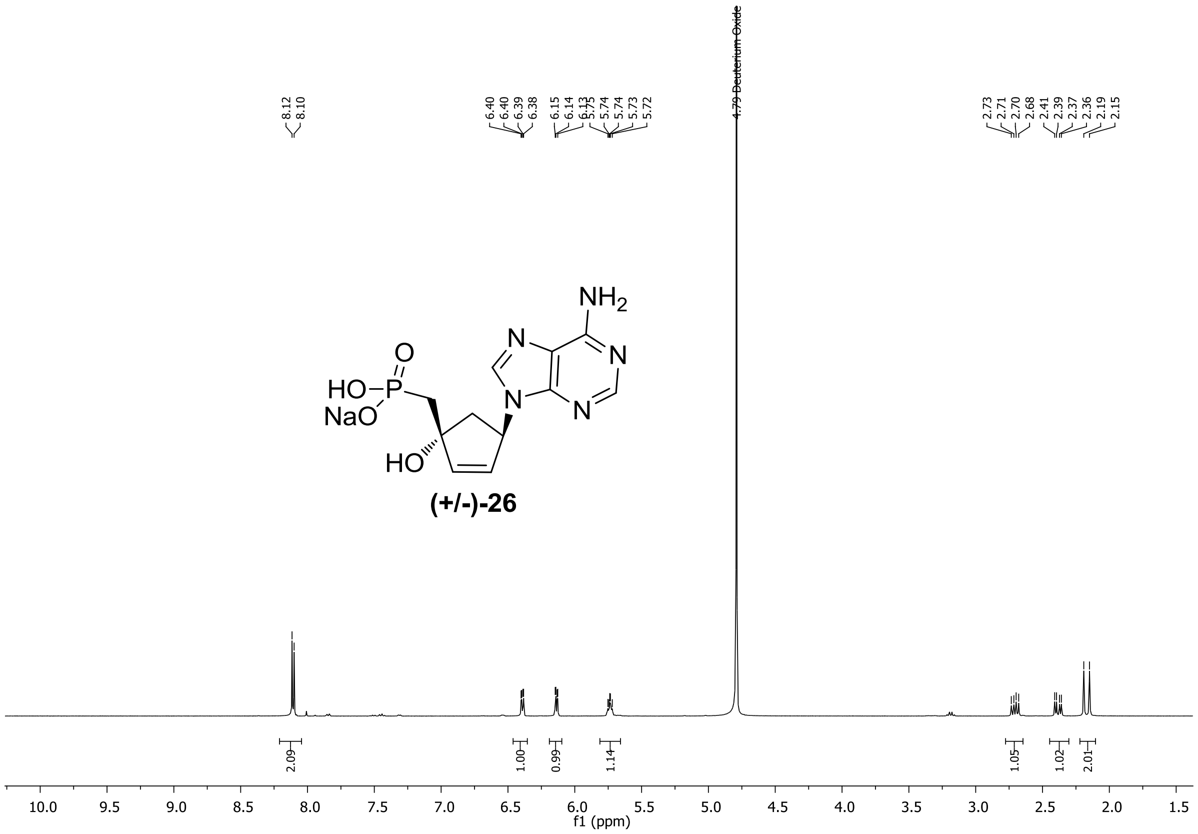
8.12
8.10
6.40
6.40
6.39
6.38
6.15
6.14
6.13
5.75
5.74
5.74
5.73
5.72
4.79 Deuterium Oxide
2.73
2.71
2.70
2.68
2.41
2.39
2.37
2.36
2.19
2.15
NH2
N
N
N
N
O
HO–P
NaO
HO
(+/-)-26
2.09
1.00
0.99
1.14
1.05
1.02
2.01
10.0 9.5 9.0 8.5 8.0 7.5 7.0 6.5 6.0 5.5 5.0 4.5 4.0 3.5 3.0 2.5 2.0 1.5
f1 (ppm)

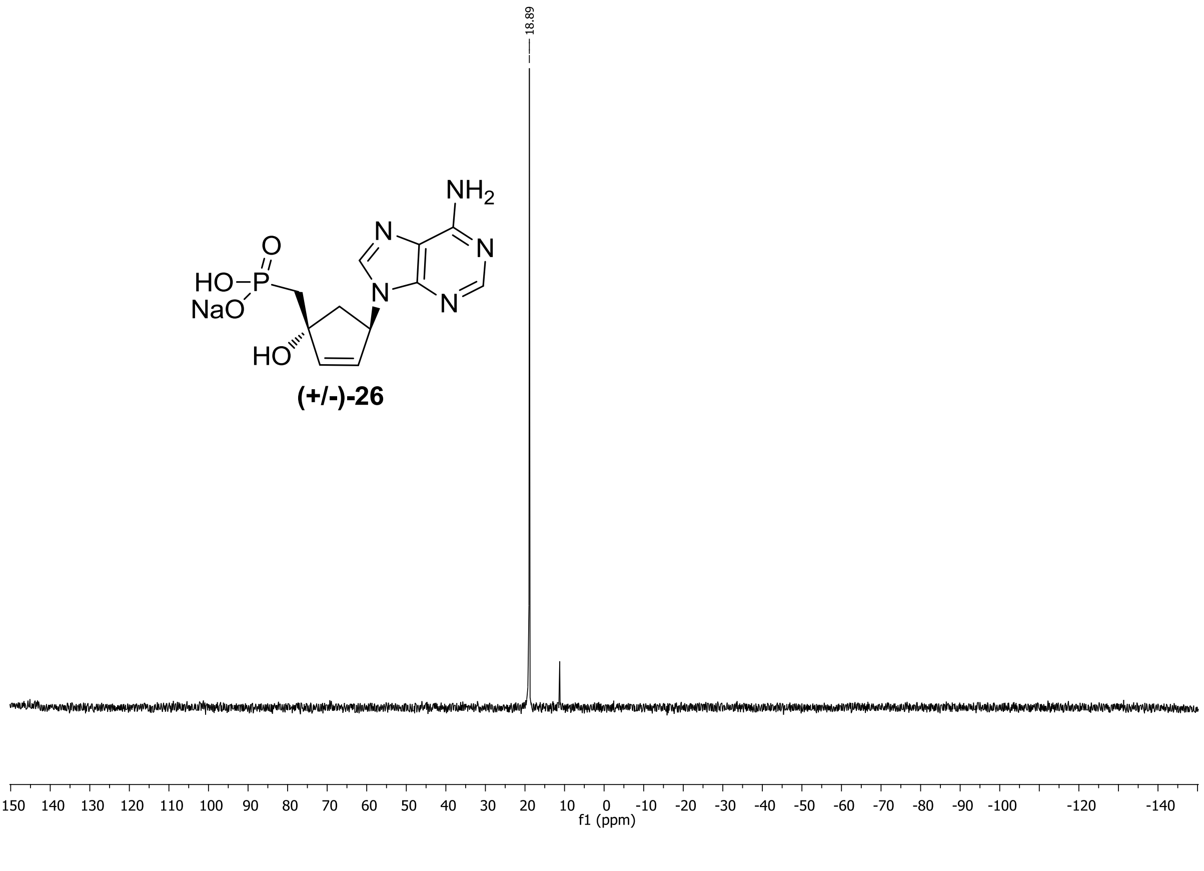
18.89
NH2
N
N
N
N
O
HO–P
NaO
HO
(+/-)-26
150 140 130 120 110 100 90 80 70 60 50 40 30 20 10 0 -10 -20 -30 -40 -50 -60 -70 -80 -90 -100 -120 -140
f1 (ppm)

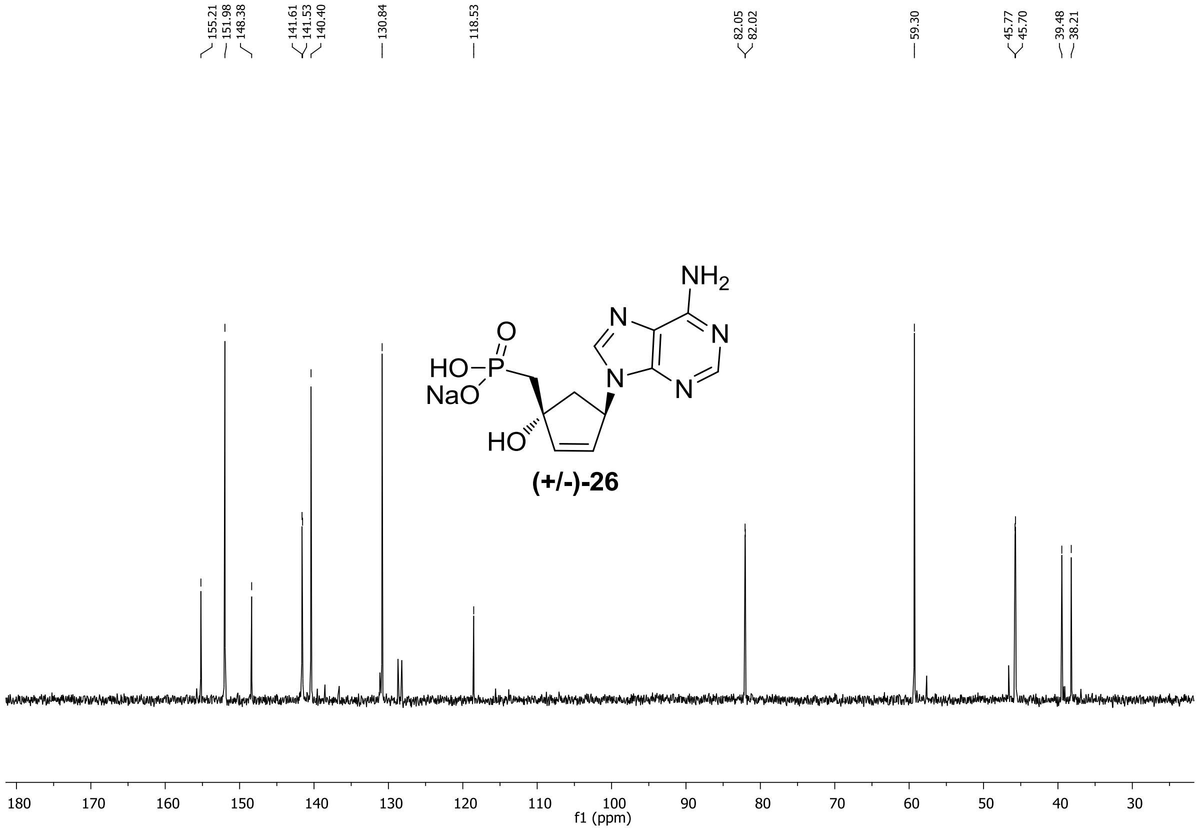
155.21
151.98
148.38
141.61
141.53
140.40
130.84
118.53
82.05
82.02
59.30
45.77
45.70
39.48
38.21
NH2
O
HO
P
NaO
N
N
N
N
N
HO
(+/-)-26
180 170 160 150 140 130 120 110 100 90 80 70 60 50 40 30
f1 (ppm)

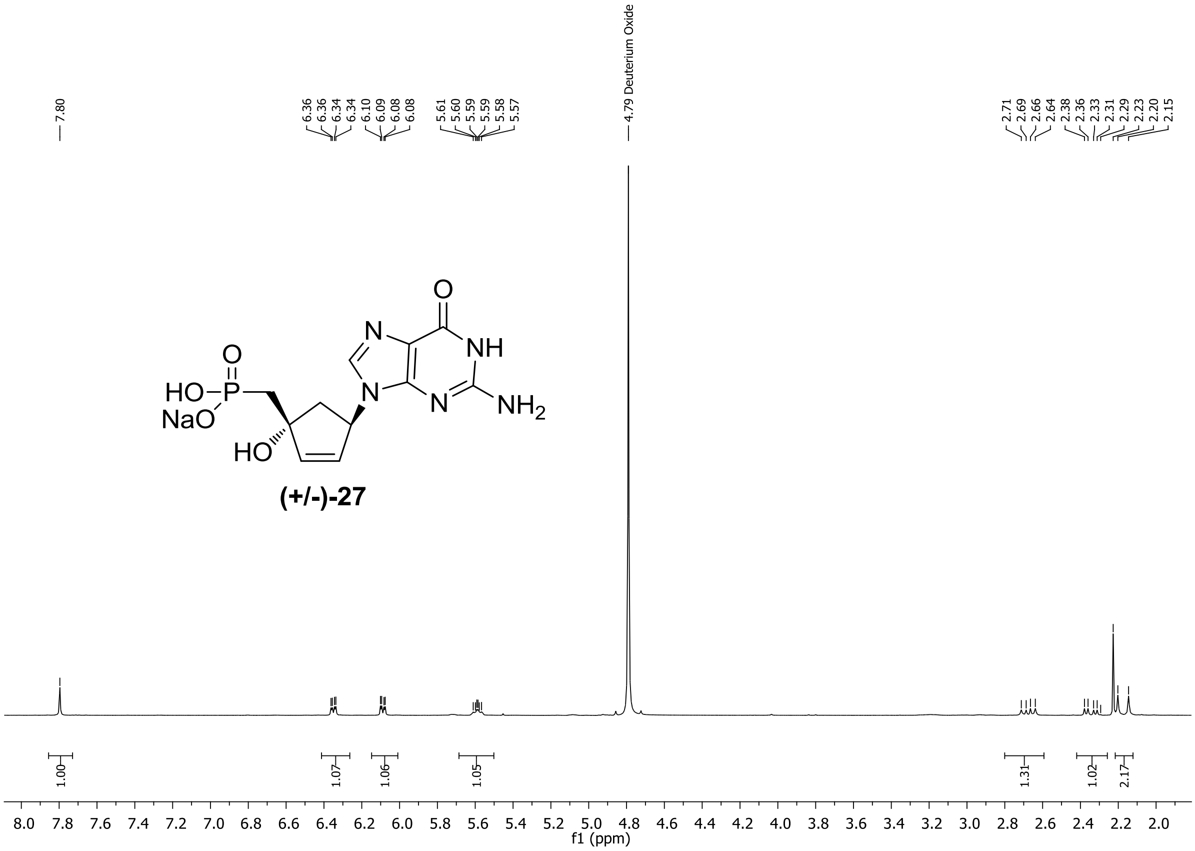
7.80
6.36
6.36
6.34
6.34
6.10
6.09
6.08
6.08
5.61
5.60
5.59
5.59
5.58
5.57
4.79 Deuterium Oxide
2.71
2.69
2.66
2.64
2.38
2.36
2.33
2.31
2.29
2.23
2.20
2.15
O
N
NH
HO
P
O
N
N
NH2
NaO
HO
(+/-)-27
1.00
1.07
1.06
1.05
1.31
1.02
2.17
8.0 7.8 7.6 7.4 7.2 7.0 6.8 6.6 6.4 6.2 6.0 5.8 5.6 5.4 5.2 5.0 4.8 4.6 4.4 4.2 4.0 3.8 3.6 3.4 3.2 3.0 2.8 2.6 2.4 2.2 2.0
f1 (ppm)

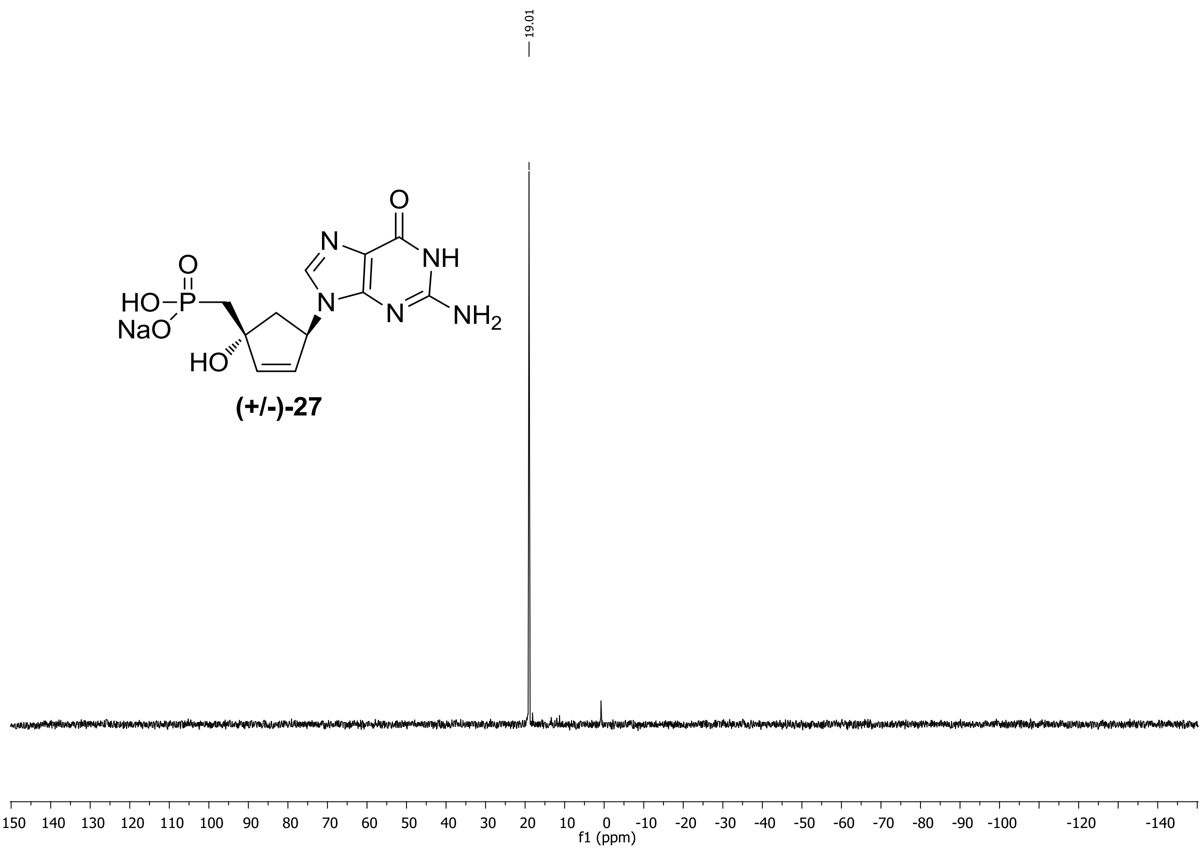
19.01
HO
NaO
O
P
HO
N
NH
O
N
N
NH2
(+/-)-27
150 140 130 120 110 100 90 80 70 60 50 40 30 20 10 0 -10 -20 -30 -40 -50 -60 -70 -80 -90 -100 -120 -140
f1 (ppm)

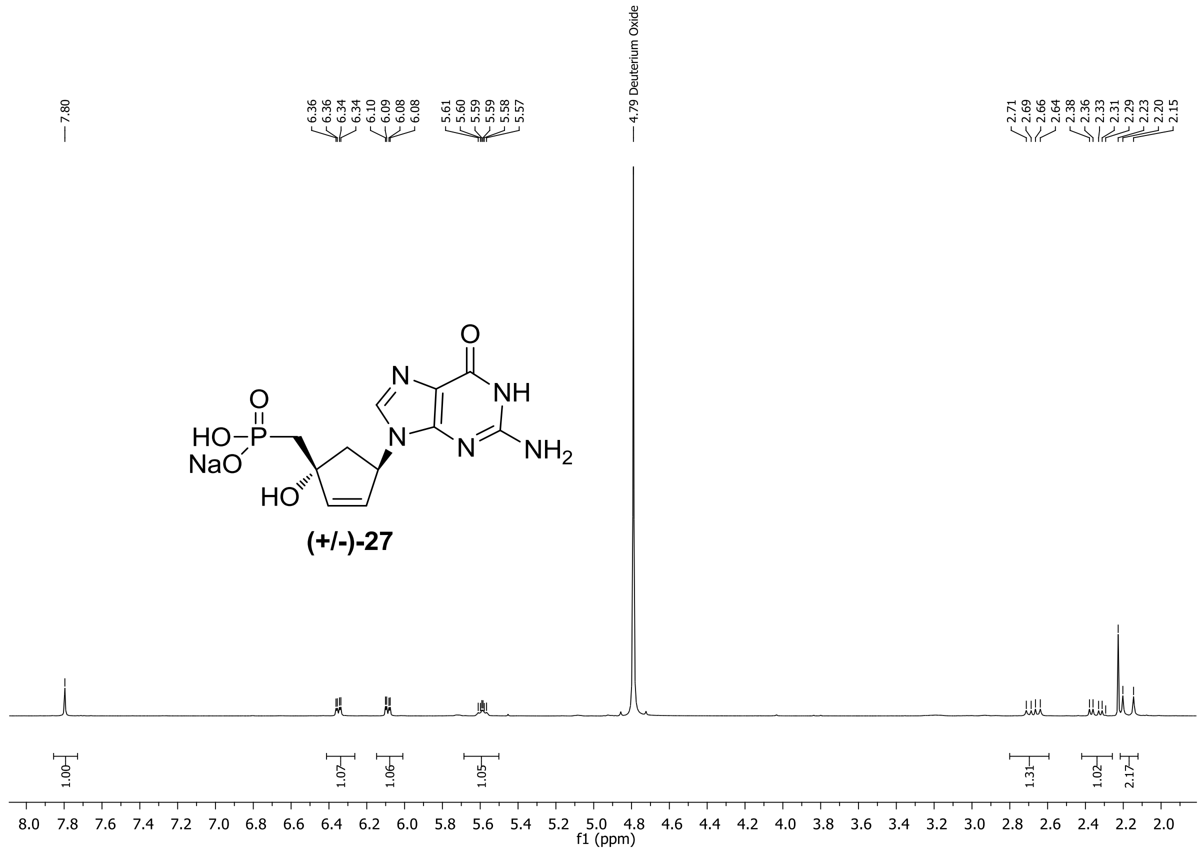
7.80
6.36
6.36
6.34
6.34
6.10
6.09
6.08
6.08
5.61
5.60
5.59
5.59
5.58
5.57
4.79 Deuterium Oxide
2.71
2.69
2.66
2.64
2.38
2.36
2.33
2.31
2.29
2.23
2.20
2.15
(+/-)-27
1.00
1.07
1.06
1.05
1.31
1.02
2.17
8.0 7.8 7.6 7.4 7.2 7.0 6.8 6.6 6.4 6.2 6.0 5.8 5.6 5.4 5.2 5.0 4.8 4.6 4.4 4.2 4.0 3.8 3.6 3.4 3.2 3.0 2.8 2.6 2.4 2.2 2.0
f1 (ppm)
